# Impact of wet-dry cycling on the phase behavior and compartmentalization properties of complex coacervates

**DOI:** 10.1038/s41467-020-19184-z

**Published:** 2020-10-27

**Authors:** Hadi M. Fares, Alexander E. Marras, Jeffrey M. Ting, Matthew V. Tirrell, Christine D. Keating

**Affiliations:** 1 Department of Chemistry, The Pennsylvania State University, University Park, PA 16802 USA; 2 NASA Postdoctoral Program, Universities Space Research Association, Columbia, MD 21046 USA; 3 Pritzker School of Molecular Engineering, University of Chicago, Chicago, IL 60637 USA; 4 Center for Molecular Engineering and Materials Science Division, Argonne National Laboratory, Lemont, IL 60439 USA; 5 Present Address: 3M Company, 3M Center, Saint Paul, MN 55144 USA

**Keywords:** Origin of life, Soft materials, Physical chemistry, Polymer chemistry

## Abstract

Wet-dry cycling on the early Earth is thought to have facilitated production of molecular building blocks of life, but its impact on self-assembly and compartmentalization remains largely unexplored. Here, we investigate dehydration/rehydration of complex coacervates, which are membraneless compartments formed by phase separation of polyelectrolyte solutions. Solution compositions are identified for which tenfold water loss results in maintenance, disappearance, or appearance of coacervate droplets. Systems maintaining coacervates throughout the dehydration process are further evaluated to understand how their compartmentalization properties change with drying. Although added total RNA concentrations increase tenfold, RNA concentration within coacervates remains steady. Exterior RNA concentrations rise, and exchange rates for encapsulated versus free RNAs increase with dehydration. We explain these results in light of the phase diagram, with dehydration-driven ionic strength increase being particularly important in determining coacervate properties. This work shows that wet-dry cycling can alter the phase behavior and protocell-relevant functions of complex coacervates.

## Abstract (web summary)

Wet-dry cycling is thought to have enabled the production of molecular building blocks of life. Here, the authors investigate the impact of dehydration/rehydration on RNA-containing complex coacervates, which are membraneless compartments formed by phase separation of polyelectrolyte solutions.

## Introduction

The potential of the wet-dry cycle to promote molecular complexity on the early Earth is increasingly supported by experiments^1–3^. Dehydration–rehydration periods are suggested to be driven not only by thermal evaporation but also by environmental phenomena, such as geyser activity, rainfall^4,5^, and salt deliquescence^6^. Even in the absence of catalysts, the wet-dry cycle has been shown to lower the thermodynamic barrier in the formation of polyacids^7^, esters^1,2^, oligopeptides^8–10^, nucleosides^3^, and polynucleotides^11,12^—molecular building blocks of life. Theoretical simulations have been proposed to explain the effects on reactivity^13,14^, while streamlined experimental setups are being explored for a better control of the process^9,15^. Despite the likelihood of prebiotic wet-dry cycling and its demonstrated benefits for polymerization reactions^11,12,16^, reports of its impact on molecular self-assembly and compartmentalization have thus far been restricted to lipid vesicles^17–19^.

Membraneless compartments, such as those produced by complex coacervation and other forms of liquid–liquid phase coexistence, are vigorously investigated for their potential to concentrate molecules and assist reactivity^20–25^. Complex coacervates result from associative charge-driven assembly of oppositely charged macromolecules^26^. The concentrated phase can form droplets when suspended in solution. With its crowded yet hydrated interior, it is a promising protocell candidate^24,27^ that can amass significant concentrations of (bio)molecules^22,24,28^ and host enzymatic reactions^20,21,23,29,30^. Extant cells benefit from related functions as coacervation is implicated in forming membraneless organelles^29,31,32^.

The impact of wet-dry cycling on complex coacervate compartments is yet to be explored. This process affects a key player in these systems, water, which has a central role in determining the structure and behavior of coacervates^33^. Beyond the hydrophilic or hydrophobic aspects of the constituents, which affect phase separation^34^, the coacervate salt content can also impact water retention^35–37^. The phase behavior of these systems can be described by diagrams such as ones that relate the polymer concentration in the mixture to that of the salt^38,39^. Choosing a composition is the same as selecting a location on the phase diagram. For specific applications, this choice is often empirical, usually done based on the desired outcome or function of the coacervate in the context of the study. However, these compartments are responsive to environmental variables, such as the ionic content of the solution^33^, pH^40^, temperature^41,42^, and different methods of preparation^23,33,43^, which alter inter and/or intramolecular interactions responsible for liquid–liquid phase separation. Using coacervate systems as models of protocells requires, therefore, taking into consideration the possible changes to the prebiotic environment around them. Beyond prebiotic research, understanding the impact of significant hydration fluctuations on coacervates is of interest to their different applications as adhesives^44^, in food systems^45^, and drug delivery^46^, as the properties of these materials are intimately related to their water content^33^.

Here, we examined the effects of wet-dry cycling on the composition, structure, and behavior of a model complex coacervate system. We chose to focus on understanding the impact on compartmentalization, one of the central functions of coacervates^24,47^. While previous studies have focused on how increases in either salt or polymer influence complex coacervates^38,42^, water loss during drying presents an unusual problem as it concentrates both salts and polymers simultaneously. We found that dehydrating a coacervate mixture decreases the potential of the polymer-rich phase to partition an RNA oligonucleotide, and enhances its diffusion. This was followed by experiments on mimics that had the same concentrations of the components during drying steps, which related the observations to the phase behavior of the complex coacervate.

## Results

### Drying and rehydration controls coacervate formation

**Fig. 1:**
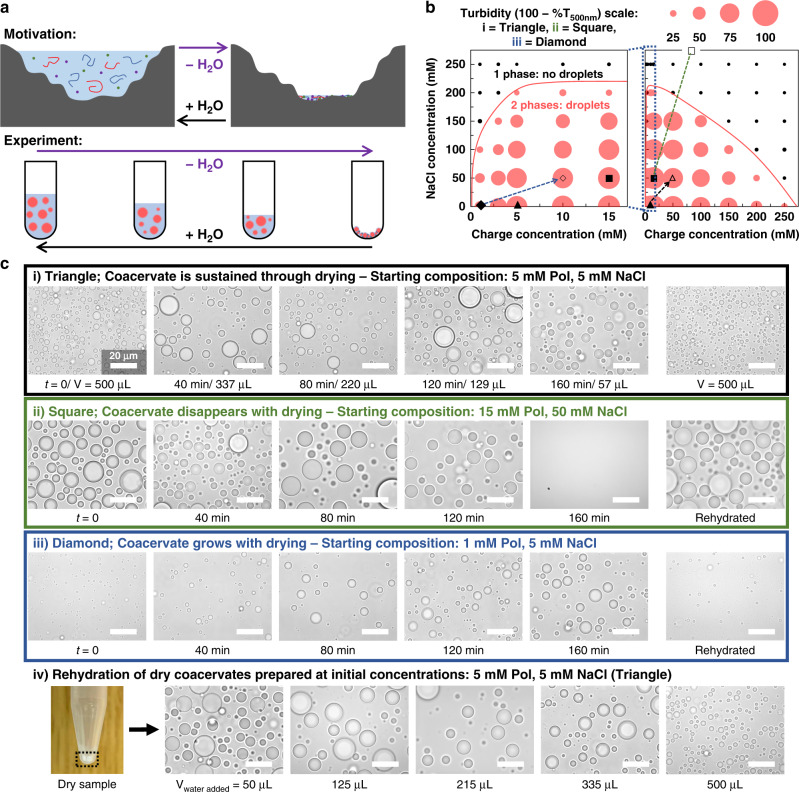
Drying and rehydrating controls the formation of PDADMA/PAA complex coacervates. **a** Scheme showing the motivation behind the wet-dry cycle experiment—an early Earth environmental scenario proposed for the emergence of chemical complexity. **b** Turbidity values for 1:1 PDADMA:PAA complex coacervates prepared at different charge and salt concentrations ([monomer charge] is calculated with respect to the concentration of the polymer repeat units). All samples contained 25 mM HEPES and 4.3 mM MgCl_2_. The plot on the left is a zoom-in on the low [charge] samples. Black markers represent turbidity below a cutoff of 20, which was chosen based on microscopy images (Supplementary Fig. 8). The width of all circles is proportional to the turbidity values of the overall samples (see legend above, T refers to transmittance). As a guide to the eye, a solid red line delimits the expected boundary between the two-phase and one-phase regions; the line is an approximate binodal curve. The charge concentration refers to one polymer type, not the total amount of both polymers. **c** Comparison between drying different 1:1 PDADMA:PAA coacervate compositions based on different starting locations on the phase diagram (i = filled triangle, ii = filled square, iii = filled diamond, with the open versions—open triangle, open square, open diamond—and dashed arrows showing the expected approximate 10× dehydration) in the time range of 0–160 min. Drying was performed in a heatblock at 95 °C; (i) shows the volume remaining at each time point (V), which is an average of 27 samples (see Fig. 2) and is applicable to (ii) and (iii) as they have the same drying rate. The dashed arrow in (ii) is expected to go up to 500 mM [NaCl] and 150 mM [polymer charge], and is thus not fully shown. (iv) Rehydration of (i) by adding a volume of water similar to the volume reached at each time point in reverse order of the top panel. All scale bars are 20 µm.

To investigate the wet-dry cycling of complex coacervates (Fig. 1a), we chose a widely studied polyelectrolyte pair, the poly(diallyldimethylammonium) PDADMA/poly(acrylic acid) PAA system with around 53 repeating units for the polycation and on average 25 for the polyanion. To the macromolecules, sodium chloride, magnesium chloride, and HEPES buffer were added (see Supplementary Note 1 for details on choosing the system and environment). While wet-dry cycling can occur in various locations, our inspiration was the fresh water of a terrestrial pond (Supplementary Note 1)^5^. Samples were dried using a heatblock at 95 °C, a temperature chosen for quicker evaporation. Supplementary Note 2 and Supplementary Figs. 1–4 detail the drying process and setup, the measurement of the evaporation rate, and the nonimpact of the temperature on the existence of the coacervate. To limit the focus to polymer and salt effects while drying, initial HEPES and magnesium concentrations were fixed at 2.5 and 0.43 mM, respectively. The starting values were chosen as to sustain the pH and not have an impact on phase separation (see Supplementary Note 3, Supplementary Figs. 5 and 6 for details).

We began by mapping the phase diagram at 1:1 PDADMA:PAA to delimit the approximate boundary between the two-phase and one-phase regimes using a combination of turbidity measurements and optical microscopy (Fig. 1b, Supplementary Figs. 7 and 8). Several initial compositions, noted on the diagram as i (filled triangle), ii (filled square), iii (filled diamond), were chosen for the investigation. Concurrent increases in all solute concentrations as water evaporates were expected to yield different diagonal shifts in compositions on the phase diagram depending on the starting composition (see dashed lines in Fig. 1b). Dehydration by ~10-fold impacted phase behavior differently for the three initial compositions.

The further the composition was on the left of the turbidity graph in Fig. 1b, a wider range of the two-phase region, where droplets exist, would be afforded to travel rightward and upward on the diagram as the concentrations of NaCl and polymers increase with water evaporation. Open markers (i (open triangle), ii (open square), iii (open diamond)) show the expected final locations of the dried samples. As seen in Fig. 1c–i, an appropriate composition which sustained droplet formation over the whole range was: 5 mM monomer charge, 5 mM NaCl, 2.5 mM HEPES, 0.43 mM MgCl_2_ (Supplementary Note 4). This composition was used for most of the following experiments, unless otherwise stated. Choosing a more concentrated composition eventually caused a dissolution of the droplets as the mixture extended beyond the two-phase region of the plot (Fig. 1c-ii). Numerous studies have shown that coacervates dissolve when conditions are pushed beyond the two-phase region. This was observed micro- and macroscopically^33,39^, and has been driven actively through enzymatic reactions^29,48^. A less concentrated point provided coacervates that would start with low turbidity (small dispersed droplets), which increased with drying as droplets grew in volume (Fig. 1c-iii, Supplementary Fig. 8). In each of the three cases, the initial composition/morphology appeared to be regained once the sample was rehydrated with a volume of water equivalent to the starting point (Fig. 1c–i–iii), or gradually (Fig. 1c-iv). Coacervate aging for 24 h, without drying, did not appear to affect its morphology when resuspended (Supplementary Fig. 9). Dry samples were also imaged in different conditions (Supplementary Note 4, Supplementary Fig. 10). We note that changes to the overall sample composition alter the relative amounts and compositions of its polymer-rich phase and dilute phase;^38^ a simple schematic for how these changes are anticipated to impact a generic coacervate system is provided in Supplementary Fig. 11. Further analysis on these changes and their repercussions is presented below.

Together these results showed that it is possible to travel into and out of the two-phase region during drying, underscoring the importance of knowing the coordinates of each system, and supporting the predictability of the wet-dry cycle’s impact on the phase behavior of coacervates—a topic that will be further explored here. Next, we chose to focus on an aspect of coacervates that is inherent to their protocell and membraneless organelles applications: compartmentalization.

### Compartmentalization behavior of an RNA oligonucleotide during the wet-dry cycle

**Fig. 2:**
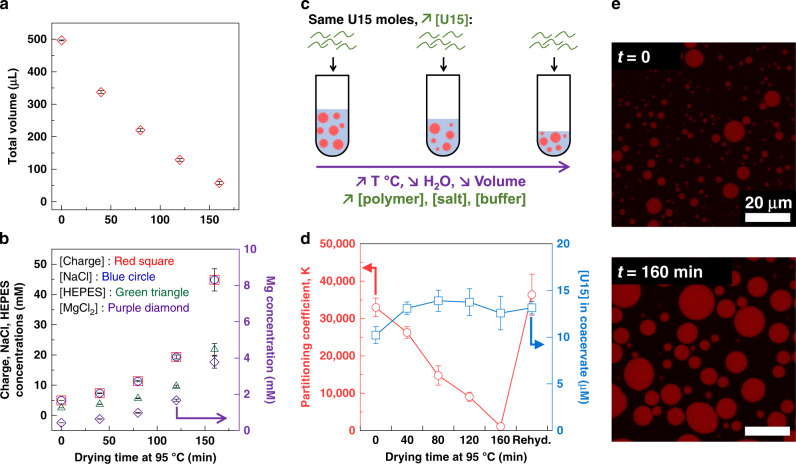
Partitioning of U15, a 15-mer oligouridylic acid, during PDADMA/PAA coacervate suspension drying. **a** Total volumes of PDADMA/PAA complex coacervate at each time point of drying. Averages and standard deviations are obtained from 9 independent trials (*N* = 27). The starting composition is the same for all samples: [polymer charge] = [NaCl] = 5 mM, [HEPES] = 2.5 mM, [MgCl_2_] = 0.43 mM. **b** Calculated increase in the concentrations of the solution components. Standard deviations are among the same trials used to calculate the volumes above. **c** To understand the partitioning of RNA within the coacervate while avoiding risk of hydrolysis during the temperature-induced drying, Alexa 647-tagged U15 was added after each drying step was complete and the solutions returned to room temperature. Fluorescent U15 was added such that its global concentration increased in the same way as all the other components in solution had, during the drying steps. Overall concentrations of U15 were as low as 0.02 µM and never increased above 0.2 µM, which is around 25,000x less than the lowest polymer concentrations, and is not believed to interfere with phase separation. **d** The partitioning coefficient, calculated as [U15]_coacervate phase_/[U15]_dilute phase_, and U15 concentrations within the coacervate after addition of the fluorescent RNA in equal numbers of moles. Means and standard deviations are obtained from three trials with analysis of 15 droplets over five images, per trial. **e** Confocal fluorescence microscopy images showing the partitioned U15 within the droplets. While the partitioning coefficients decrease, the internal concentrations do not change significantly as quantification in **d** and Supplementary Fig. 14 show. Individual data points are shown in Supplementary Fig. 14.

As the water evaporated and the total volume decreased during drying (Fig. 2a), the concentration of each component increased (Fig. 2b). We did not observe changes to the volume of coacervate formed, based on visual estimates (Supplementary Fig. 15b). To understand the changes to the coacervate as the water content decreased, partitioning of a short fluorescently-labeled RNA oligomer, a 15-mer oligouridylic acid U15, was performed, as RNA, with its versatility^20,21,49^, is thought to play a central role in prebiotic chemistry^50^. The labeled U15 partitioned strongly into the coacervate phase, with >30,000-fold greater local concentration inside than outside the droplets (Fig. 2c–d; with 0.02 µM U15 added globally before drying, 10 µM were measured inside the droplets and around 0.3 nM outside).

After each drying step, U15 was added in concentrations that matched the drying-induced increase in components concentration (Fig. 2c). As seen in Fig. 2d, the measured concentration of the fluorescent U15 in the coacervate phase stayed constant throughout the process, despite the increase in global U15 concentration during dehydration (see Fig. 2e, Supplementary Figs. 12 and 14). At the same time, U15 concentration in the dilute phase increased from less than 1 nM up to 12 nM (Supplementary Table 1). The partitioning coefficients, or partition ratio, K = U15_coacervate phase_/U15_dilute phase_, decreased from >30,000 to around 1000 (Fig. 2d). In another experiment, the oligomer was added at a constant concentration of U15 (0.02 µM). As seen in Supplementary Figs. 13 and 14, the partitioning coefficients were similar in the two experiments, regardless of the initial [U15] added (Supplementary Fig. 14e–f) and measured in the coacervate phase.

The correlation between K and the decreasing total volume followed a linear trend as the coacervate phase volume stayed constant (Supplementary Fig. 15). While the ratio of the dilute phase/coacervate phase volumes decreased, the preference of U15 to go into the polymer-rich phase decreased, even if more of the nucleic acid was available in the dilute phase (Supplementary Fig. 15, Supplementary Table 1). Zeta potential measurements ruled out the effect of the coacervate charge on partitioning (Supplementary Note 5, Supplementary Fig. 16). The observed decrease in partitioning suggests that the coacervate phase paradoxically could become more hydrated as the overall sample dehydrated, due to increasing polyelectrolyte and salt concentrations. This, along with an increase in charge screening would render the two phases more similar and reduce the strong preference of the U15 for the coacervate phase. Coacervate compartmentalization has been shown to exhibit a close relationship between partitioning and molecular mobility^20,47^. To further explore the RNA behavior, we investigated U15 diffusion within the coacervate.

**Fig. 3:**
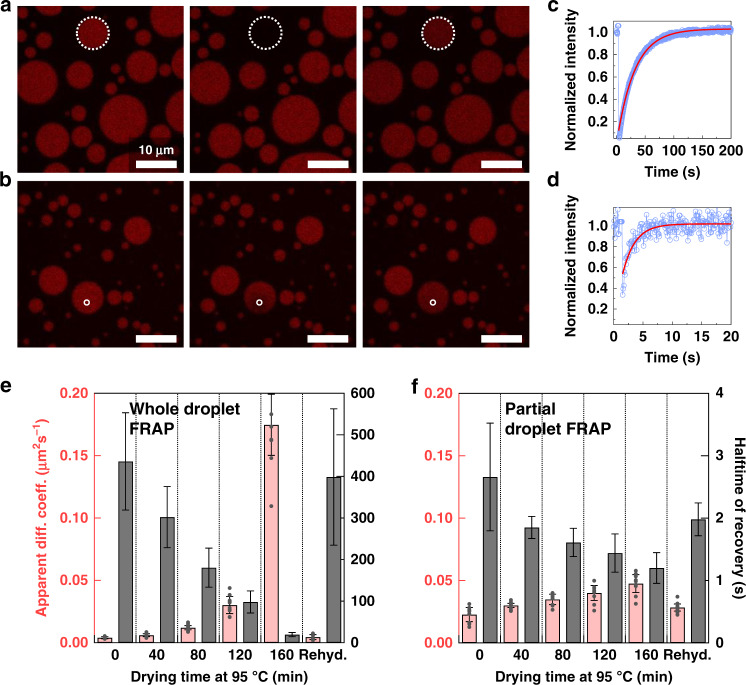
Dynamics of U15 in the complex coacervate during drying. **a** Fluorescence recovery after photobleaching, FRAP, over a whole 1:1 PDADMA:PAA coacervate droplet in solutions dried at 95 °C for 160 min and allowed to return to room temperature before partitioning Alexa 647-U15 within. **b** FRAP over part of the same coacervate in solutions dried at the same temperature for 80 min and allowed to return to room temperature before adding U15. **c** Recovery of the fluorescence after whole-droplet photobleaching of U15 partitioned within a coacervate in mixtures allowed to dry for 160 min. **d** Recovery of the fluorescence after partial-droplet photobleaching of U15 partitioned within a coacervate in mixtures allowed to dry for 80 min. Circle markers represent corrected fluorescence intensity in **c, d**. Lines are fitting results according to equations mentioned in the Methods section. **e** Apparent diffusion coefficients and recovery halftimes of U15 as it recovers after bleaching of whole coacervate droplets at each time point of the drying experiment. **f** Apparent diffusion coefficient and recovery halftimes of U15 as it recovers after bleaching of partial droplets at each time point. The bars are averages of five trials with at least two droplets each in **e**. In **f**, the bars are averages of three trials with at least three droplets analyzed in each. All individual data points are shown in Supplementary Tables 2 and 3, and as gray markers for the apparent diffusion coefficients in **e** and **f**. The error bars represent the standard deviations among all trials.

Fluorescence recovery after photobleaching (FRAP) experiments were performed to investigate the movement of U15 between the coacervate droplets and the surrounding dilute solution (whole-droplet FRAP) and within the droplet itself (partial-droplet FRAP). Figure 3a, b depicts representative samples of these experiments showing the whole-droplet FRAP of the 160-min sample and the partial-droplet FRAP of the 80-min sample, respectively. Figure 3c, d shows the fluorescence recovery profiles within these samples. The apparent diffusion coefficients, obtained from the recovery halftime values (*τ*_1/2_) (which are also shown) and the photobleached areas radii, are shown in Fig. 3e, f for the whole-droplet and intradroplet FRAP experiments, respectively. We note that these data are most useful for comparing between similar datasets, and not between the two experiment types, to understand different dehydration states rather than as a means of quantifying true molecular diffusion^51^. The slow diffusion in the initial samples can be correlated with the high partitioning observed in these samples in Fig. 2. The photobleached U15 was almost immobilized within the droplets in these samples as the exchange rate with the surrounding solution was low.

RNA recovery within the whole droplet showed an increasing trend as the water was lost from the coacervate mixture (Fig. 3e). While the upper bound of apparent diffusion coefficients after tenfold dehydration was close to 0.2 µm^2^ s^─1^, these values are considerably lower than their expected diffusion in buffer of around 150–300 µm^2^ s^─1^^20,52^. Values ranging between 0.005 and 1.9 µm^2^ s^─1^ were previously measured for various RNA 15-unit oligomers partitioned within a polyU/spermine coacervate^47^. The difference in the diffusion within the droplets was not as significant, merely doubling between the first and the last time point—spanning a decrease of 8 to 10-fold in sample volume (or similar increase in concentrations, Fig. 3f)—and rehydration recovered the initial values. This implied a ~2-fold decrease in the coacervate viscosity. The effect of slow whole-droplet recovery can be further seen in the example shown in Fig. 3b as, even though the photobleached region of the droplet recovered quickly from the supply of RNA in its immediate proximity, the whole-droplet fluorescence intensity slightly decreased as it took longer for this supply to be replenished from the dilute phase. This also emphasizes that the apparent diffusion coefficients are standardized to the size of the bleached area: even the highest whole-droplet diffusion coefficients correspond to a slower recovery than the slowest partial-droplet coefficients (whole droplet 160 min: 20 s, partial droplet 0 min: 2.7 s—gray bars in Fig. 3e, f). Repeating the exchange experiment with a constant concentration of U15, instead of increasing them like here, disproved the hypothesis that the increase in RNA mobility could be a concentration effect (see Supplementary Note 6, Supplementary Fig. 17, Supplementary Table 4).

### Mechanism of RNA partitioning and kinetics during drying

Combined, the partitioning and FRAP results lead to a better understanding of the observed nucleic acid compartmentalization behavior during dehydration. The strong accumulation of RNAs within the coacervate phase of related systems has been previously observed and attributed to electrostatic attraction, which can be coupled with displacement of polyanions within the network^20,47,53^. The partitioning values and the apparent diffusion coefficients are the consequence of the complex nucleic acid dynamics towards, outward from, and within the coacervate phase^20^. The dynamics, which are governed by charge screening, appear to be directly related to partitioning: the change in whole-droplet diffusion coefficient (~40-fold increase) was in the opposite direction, but similar magnitude, as the partitioning coefficient (~30-fold decrease). Hints of a strong correlation between fast whole-droplet recovery and low partitioning have been observed before^20,47^. Salt has been shown to decrease the entropy penalty for partitioning of charged polymers^54^. Elevated levels of charge screening cause the polymer to behave like an uncharged molecule which decreases its preference for one or the other phase^54,55^. Here, a general trend of this relationship was elucidated.

Furthermore, even though slower kinetics could be expected from an increase in polymer concentration, a simultaneous increase in hydration, which would cause a reverse effect, has been observed before with coacervates made of lysine and glutamic acid polymers^38^. This phenomenon, which has been ascribed to self-suppression of complex coacervates^56^, was implied for diagonal movements along the charge-equivalence line on the phase diagram^38^. While the repercussion of this phenomenon could be a slight decrease in coacervate volume, this can be counterbalanced by an increasing salt concentration, accompanied by an increase in water content^38^. Higher polymer concentrations have also been shown to promote compartmentalization in two-component systems with low salt concentrations^54^. This increases the mixing enthalpy and enhances partitioning, but also raises the water content of the coacervate phase^38^ which brings its hydration level somewhat closer to the dilute phase.

An expanded discussion on expected partitioning of polymers between the two phases and its impact on the observed behavior is included in the Supplementary Discussion 1 and Supplementary Fig. 18. As the polymer-rich phase becomes less concentrated in polymers, more hydrated, and more charge screened—in its approach to the critical point where the compositions of the two phases eventually become the same—the outcome is faster diffusion and lower preference of the guest molecule to one or the other phase. In general, the behavior of the U15 RNA used here can be interpreted as that of a polymer undergoing similar effects as the ones expected for other macromolecules. The decrease in the RNA partitioning is manifested by a constant concentration inside the polymer-rich phase accompanied by an increase in the dilute phase (see Supplementary Discussion 1). We next tested how the expected periodicity of wet-dry cycling would impact the compartmentalization behavior of the coacervates used here.

### Impact of repeating wet-dry cycles on coacervate compartmentalization

**Fig. 4:**
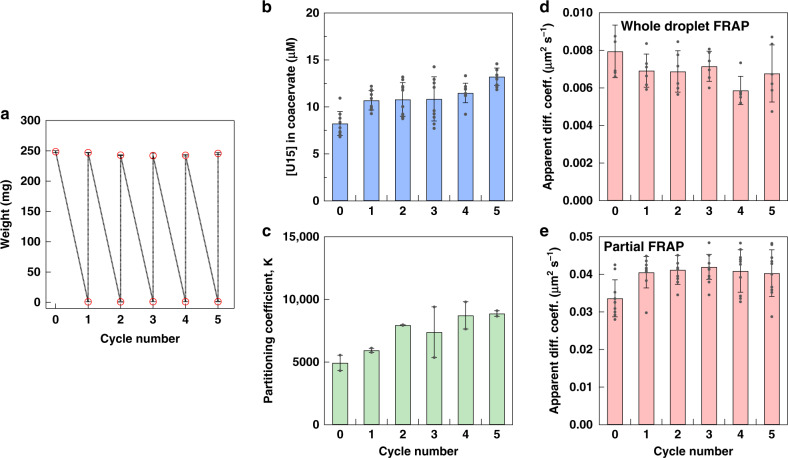
Impact of repeating wet-dry cycles on the complex coacervate. **a** PDADMA/PAA coacervates ([Charge] = [NaCl] = 10 mM, [HEPES] = 5 mM, [MgCl_2_] = 0.9 mM) were fully dried and rehydrated for five consecutive cycles. The plot shows the average weights after drying-rehydration. **b** The concentration of U15 in the same coacervate after rehydration of samples obtained by confocal microscopy using Alexa 647-U15 as a probe. **c** The partitioning coefficient, K, taken as [U15]_coacervate phase_/[U15]_dilute phase_, after each cycle. **d** Apparent diffusion coefficients obtained from halftime of fluorescence recovery during FRAP experiment where whole coacervate droplets, containing the Alexa 647-U15 probe, were bleached. **e** Apparent diffusion coefficients in FRAP experiments where a 1-µm diameter region within the coacervate phase was bleached. The data in **b**–**e** are averages and standard deviations of two trials with an analysis of 10 droplets total for **b**, with the same values used when dividing by the [U15] in the dilute phase in **c**, six droplets in **d**, and 10 intradroplet areas in **e**. Individual data points are shown as gray markers. Supplementary Tables 5 and 6 include all FRAP fitting parameters.

Multiple wet-dry cycles, which could last from few minutes to few hours, are usually employed to increase reaction yields and prove their robustness in extreme environmental variability^1,2,12^. We sought to expose the PDADMA/PAA coacervate compartments to multiple cycles to check whether they survive these conditions, and to understand the impact on their RNA partitioning. Repeatedly drying and rehydrating the coacervate was monitored by weighing the contents of the tubes (Fig. 4a). The solution pH remained the same during these steps, with a slight decrease observed after the first cycle (Supplementary Fig. 19). The concentration of U15, added at the same concentration after rehydrating all samples, was around 10 µM after five cycles (Fig. 4b, Supplementary Fig. 20). A slight increase was observed between the initial sample and the rehydrated ones, similar to the outcome of the single-cycle experiment in Fig. 2. While vortexing, then pipetting, was employed to resuspend the dry coacervate, this increase in [U15] within the coacervate could be related to some coacervate not being resuspended completely. This could also explain the slight drop in pH values after the first cycle as some HEPES would be stuck with the unsuspended coacervate. Supplementary Note 7 details the statistical analysis showing this difference; while the last cycle is also shown to be slightly different, the discrepancy is less significant. The partitioning coefficient did not vary significantly (Supplementary Note 7), indicating that the U15 amount in the supernatant was similar, around 1–2 nM, among samples (Fig. 4c, Supplementary Table 7). The FRAP experiments in Fig. 4d, e showed that the dynamics of U15 remained unchanged after cycling the samples between dry and wet states which demonstrated that the coacervate integrity and viscosity were regained after each rehydration step. It is worth noting that the apparent diffusion coefficients were close to the second time point in Fig. 2, in which the same concentrations of components as the starting ones in this experiment were reached. Overall, these experiments revealed the minimal impact of repeated cycles on the compartmentalization potential of complex coacervates.

### Comparing the dried samples with composition mimics

**Fig. 5:**
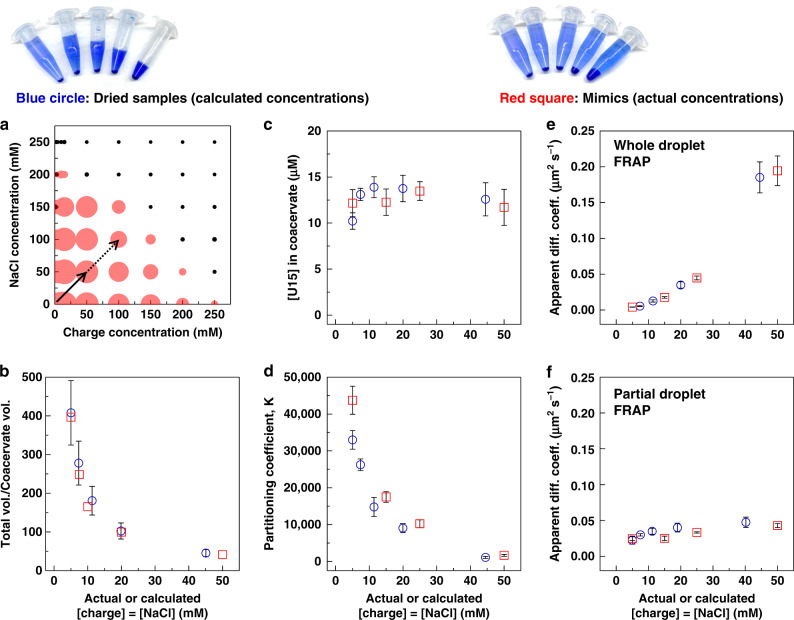
Comparison between dried coacervates and composition mimics samples. **a** The solid line on the phase diagram, obtained by turbidity measurements, shows the range of the composition mimics. The dotted line shows the extension where the SAXS measurements were performed (Fig. 6). **b** Total volume of solution to coacervate phase volume ratio. The error bars in **b** represent a constant standard deviation of ±0.25 µL, a difference in volume that was difficult to discern with the naked eye (Supplementary Fig. 22). **c** U15 concentrations measured within the coacervate. **d** Partitioning coefficient of U15 ([U15]_coacervate phase_/[U15]_dilute phase_). **e** Whole-droplet FRAP, and **f** partial-droplet FRAP in dried PDADMA/PAA coacervate samples and mimics with compositions on the same range obtained during drying. Error bars in **c–f** represent standard deviations among different trials and individual data points are shown in the SI (Supplementary Fig. 24, Supplementary Tables 8 and 9). In **c**, mimics data represent means and standard deviations of three trials with 15 droplets analyzed over three images, per trial. FRAP mimics data are averages and standard deviations obtained from three trials with at least two bleached droplets per trial for whole-droplet experiments, and three bleached areas per sample for partial-droplet measurements. Photos of bromophenol blue-dyed dried and mimics samples are included above for clarification.

Thus far, we have discussed the effects of drying and rehydration on complex coacervates. While this process, and its impact on compartmentalization, were expected to be grounded in movements on the phase diagram (dashed lines in Fig. 1), we sought to validate whether these movements were indeed dictating the resulting behavior. Most experiments above follow a movement that has been theoretically described as traversing a charge-equivalence line^38^. If the implication is correct, we hypothesized that it should be possible to recreate the drying process by making samples with increasing concentrations of all components—composition mimics—with the same total volume. This experiment would also help us verify whether the cycling process affects the linear movement, for example by damaging reactants or through failure of reincorporating large amounts of the dry materials into the rehydrated coacervates. Mimics that contained the same polymer, salt, and buffer components were prepared with starting concentrations that would fall on the same expected line as the drying process (Fig. 5a).

These composition mimics samples were prepared at the same total volume as the initial sample in all the above experiments (500 µL, Fig. 5a). Figure 5 and Supplementary Fig. 22a show samples that were dyed with bromophenol blue and centrifuged to show the difference between dried coacervates and mimics. A visual calibration curve was prepared to estimate the volumes of the concentrated phase (Supplementary Fig. 22b). The total volume was obtained by conversion from net weights (Supplementary Fig. 23). The first sign that the coacervate mimics and the dried ones, while having different total volumes, were similar was the almost identical ratio of V_coacervate_/V_total_ (Fig. 5b). To check whether this similarity translated into the same compartmentalization behavior of coacervates, U15 partitioning and FRAP experiments were performed. Figure 5c–f shows that these partitioning and FRAP results were very similar between the dried samples and the mimics. While the starting values in Fig. 5d are slightly different, the trend in percentage decrease is almost identical (Supplementary Note 7, Supplementary Fig. 21). The pH of the mimics also stayed constant, following a similar trend to the dried samples (Supplementary Fig. 25). These comparisons emphasize the importance of the volume ratio of the coacervate to the total volume, rather than the volume itself, in determining its properties and behavior. We note that these volume fractions reflect changes in the composition of the coacervate and dilute phases, which define the observed effects on the RNA partitioning and diffusion (see Supplementary Discussion 1). Such an outcome gives confidence in results of experiments that are performed with minimal volumes when the materials are limited. The mimics experiment also demonstrated the predictability of the drying behavior from samples prepared from scratch.

To understand the impact of the observed movement through the phase diagram on the internal structure of the coacervate and its possible relationship to the compartmentalization behavior, we turned to small-angle X-ray scattering (SAXS). SAXS enabled us to investigate the conformation of polymers in solution over multiple length scales simultaneously. This technique has been applied to similar systems to understand the effects of increasing the concentration of one component—polymer or salt alone^35,57^. In general, as salt is added and electrostatic interactions are screened, polyelectrolyte chains decrease in effective charge density and more closely resemble neutral polymers in solution by SAXS^35,37^. In our case, we increase both polymer and salt concentrations, in addition to buffer and magnesium amounts, to mimic the impact of drying on the structural level (Fig. 5a). The aim of this experiment was to explore the molecular-level effects of this realistic environmental change. Samples with lower polymer and salt concentrations (below 50 mM for both) were omitted from the analysis as they did not scatter well because of the small volume of coacervate material. Samples with 50–100 mM concentrations scattered better and were qualitatively analyzed to obtain an idea of the coacervate network behavior along the same charge-equivalence line on the phase diagram (sample details are in Supplementary Table 10, dotted line in Fig. 5a).

**Fig. 6:**
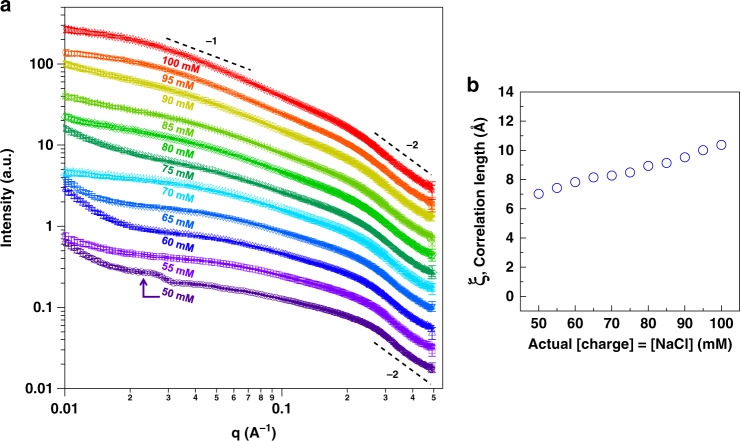
SAXS data of complex coacervate mimics samples. **a** Small-angle X-ray scattering plots of PDADMA/PAA complex coacervate samples prepared at 50–100 mM polymer charge and NaCl, with HEPES ranging between 25 and 50 mM, and MgCl_2_ between 4.3 and 8.6 mM for the same samples. The data were shifted vertically for clarity (Supplementary Fig. 26a shows the data without vertical shift). Error bars represent noise in the scattering measurements. **b** Correlation length, ξ, obtained from the Unified fit in the high-q region of the SAXS plots. The individual fits are shown in Supplementary Fig. 26b. Error bars obtained from fits were smaller than the markers and were not included. Their values can be found in Supplementary Table 11.

All scattering profiles of analyzed samples had a slope of–2 at high-*q* (i.e., at the smallest length scale) which showed the behavior of a Gaussian chain exhibiting a random walk—an observation that has been reported for many other polymer coacervate systems in the literature^35,37,57^ (Fig. 6a, Supplementary Fig. 26a). At larger length scales (mid-*q*), the slope was shallow at the lower concentrations but showed an *I(q)* ~ *q*^*−1*^ dependency at higher concentrations. The latter indicates rod-like structures which, along with the increase in scattering seen at a length scale of 10–40 nm (obtained using an estimated direct conversion of 2*π*/*q*), could be attributed to the dense tubule diameters that have been observed in cryo-transmission electron microscopy measurements^58,59^. We also note that a small peak around 0.025 Å^−1^ in the 50 mM sample could be related to long-range electrostatic repulsion, as expected from a highly charged sample (see Supplementary Fig. 16). The upturn in the scattering at low-*q* could point to larger aggregates as all concentrations increase and along with them, the hydration level. Future investigations could probe this larger length scale further.

In the high-*q* region, a broad low-intensity peak around 0.3 Å^−1^ in the lower concentration samples appeared to shift to lower-*q* in the higher concentrations. Such a peak is widely reported in the literature to originate from a distinct polyelectrolyte correlation length, ξ, which is related to the interactions between the polymeric chains^37,55,60^. The intensity of the peak is sharp in concentrated polyelectrolyte solutions; in general this is highly dependent on the polymer concentration which, if increased, causes a shift to higher-*q* values (corresponding to smaller lengths or mesh sizes)^55^. However, its intensity drastically decreases in the presence of salt, which weakens the electrostatic repulsions between chains, or when the polyelectrolyte associates as part of a complex mixture, such as in complex coacervates^35,37^. With the help of the Unified fit, applied to one structural level^61,62^, the approximate size of this length was obtained (Fig. 6b, Supplementary Fig. 26b and Supplementary Table 11).

The increase in the size of the correlation length, from around 7 to around 10.5 Å, pointed to the noteworthy influence of salt screening on the structure of this complex coacervate during the simultaneous increase of both polymer and salt concentrations. The observation along this line in the phase diagram supports the partitioning and diffusion behavior of the U15 seen above, as salt screening appeared to be the key player in these results. Also, a less polymer-dense network deduced from the mid-*q* region, and the increase in ξ, implied a more hydrated coacervate phase.

### Changing the directions of compositional change

**Fig. 7:**
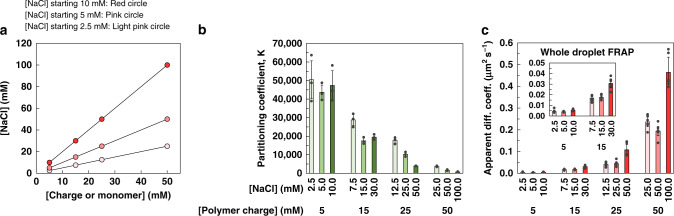
Changing the directionality of movements on the phase diagram. **a** Plot showing the change in salt concentrations in experiments that represent different compositions on the PDADMA/PAA phase diagram. HEPES and MgCl_2_ were added at 2.5 and 0.43 mM initially and increased in all samples with the same factor of increase of NaCl on the *y*-axis. **b** Partitioning coefficient, taken as [U15]_coacervate phase_/[U15]_dilute phase_, with different concentrations of salt for the same polymer concentrations. **c** Whole-droplet FRAP experiments of the same range of samples. The inset corresponds to a zoom-in on the low concentration samples. Supplementary Table 12 includes all the fitting parameters. Partitioning data are averages and standard deviations obtained from three trials. Means and standard deviations in FRAP are obtained from three samples with two bleached droplets per sample. Individual data points are shown as grey markers.

While changes in starting compositions caused a predictable control over the formation and disassembly of the coacervate (Fig. 1), we next explored the effects of more systematic variations over the guest RNA, using more mimics. As the mimics experiments above focused on moving along a charge-equivalence line, we examined the impact of traversing other linear directions within the phase diagram on the coacervate compartmentalization capacity, by changing the starting concentration of one component. Figure 5 showed that the drying behavior can be predicted from such samples. Therefore, to mimic drying here, samples were prepared with various salt concentrations for a fixed range of polymer concentrations (Fig. 7a, Supplementary Fig. 27). The opposite—changing the polymer concentrations with fixed [salt]—was also investigated (Supplementary Fig. 28). The initial U15 added in these experiments was increased with the polymer concentration, to mirror the drying effect. In Fig. 7, it increased from 0.02 to 0.2 µM in the variable salt concentrations mimics.

Varying the salt concentrations around a starting value of 5 mM with a fixed range of polymer concentrations starting at 5 mM did not affect the concentration of U15 partitioned inside the coacervate, consistent with what was observed in the drying experiment (Supplementary Fig. 27b). Salt was varied in a linear fashion, similar to drying samples, with values ranging between 2.5 and 25 mM in the lower concentrated samples and between 10 and 100 mM in the higher concentrations. In the same samples, the partitioning coefficient decreased with increasing salt concentrations towards the upper section of the range (Fig. 7b), which directly validated the impact of salt screening on the partitioning behavior. Similar experiments performed with varying polymer concentrations in a fixed salt range had an impact on the U15 concentrations inside the coacervate phase which decreased, but the trend in partitioning was again similar to, but slightly shallower than, the salt experiment (Supplementary Fig. 28). The mechanism of partitioning above and SAXS results pointed to an increase in hydration with more polymers, accompanied by a changing volume fraction, which would explain the effect on the U15 concentration inside the coacervate phase.

The whole-droplet FRAP response in the experiments where the concentration of salt was varied showed an increasing apparent diffusion coefficient, similar to the drying experiment. Among the samples that had the same polymer concentrations, the highest concentration of salt exhibited the fastest kinetics (Fig. 7c). The difference in the lower salt concentration samples (2.5–25 mM NaCl) was not as distinct. The effect of salt screening on polyelectrolyte diffusion has been shown to be nonlinear^63^. Interestingly, the samples that had different polymer concentrations for the same salt concentrations also showed increasing whole-droplet diffusion trends (Supplementary Fig. 28, Supplementary Table 14). The universal correlation between faster kinetics and lower partitioning in these experiments mirrored drying experiments. As for partial-droplet FRAP, the increase in kinetics was again less pronounced (Supplementary Figs. 27c and 28e, Supplementary Tables 13, 15), revealing how it was harder to affect the internal viscosity of the coacervate by changing the concentrations in this range. This pointed, again, to the importance of whole-droplet exchange, over internal viscosity, in determining exchange kinetics with the surroundings.

## Discussion

The experiments in this paper point to an intriguing richness in phase behavior and physical properties of PDADMA/PAA complex coacervates as they dry and rehydrate, and to how these changes can be understood using the phase diagram. The experiments with mimics successfully linked the wet-dry cycling process to movements on the phase diagram. They also provided structural insights on the expanding polymeric network as this distinctive linear compositional change occurred, and predicted the effects of varying the direction of these movements. Based on the generality of the ionic strength and (de)hydration effects underlying these behaviors, we anticipate that similar wet-dry cycling response may be possible for other complex coacervate systems.

Repeated wet-dry cycles did not seem to impact the integrity and dynamic response of the coacervate. During a single cycle, the transition between different hydration states did not significantly affect the local concentration of RNA inside the coacervate droplets over a 10-fold decrease in overall sample volume and concomitant increase in total concentrations of RNA and other components. Hence, the coacervate phase essentially buffers the local RNA concentration during large environmental changes, even while it changes substantially outside the droplets. From a coacervate perspective, our analysis emphasizes that changes in the overall system do not necessarily correlate with proportional changes in the composition of each phase, and can be understood through the phase diagram. From a protocell perspective, this very primitive form of proto-homeostasis might be beneficial for early cellular function. At the same time, changes in RNA exchange between the coacervate droplets and the exterior continuous phase, and in the relative amounts of nucleic acid inside versus outside the compartment, were substantial. As has been discussed by Jia et al., high exchange rates could hinder evolution of RNA as its segregation within individual compartments is short-lived^64^. However, RNA exchange rates between coacervate and continuous phases, and ultimately between coacervate droplets, can be controlled by manipulating the interaction strength of the compartment’s charged components^53^ or preparing coacervates with microfluidics^65^. The present work suggests that considering the trajectories of such systems through composition space during hydration cycling, afforded by an environmental process, can provide an additional mode of control in which populations of membraneless compartments cycle between states with higher and lower inter-droplet RNA exchange.

Furthermore, controlling the drying rate would allow a balance over the duration where high partitioning is obtained versus that during which fast kinetics are predominant, depending on the requirements of the experiment. It is thought-provoking to consider that the composition (as well as macromolecules and salt types) and drying method of various ponds on an early Earth could have led to a large number of dehydration processes, providing access to compositionally and functionally different coacervate populations, even if the available components were limited. Considering several recent reports that discuss the potential of coacervates to modulate the reactivity of ribozymes and other enzymes^20,21,53^, the changes observed here are expected to result in a wide array of reactivity profiles within the concentrated phase. As such, it is important to keep in mind that an optimal composition for a certain function of complex coacervates could be ideal for a limited range of concentrations (of both polymer and buffer molecules). For better or worse, this range is easily changeable in a transient natural system, as we show here. Outside the scope of origins of life research, the conditions imposed on coacervates by wet-dry cycling could be of interest to their use in other fields. For example, this study demonstrates that their cargo-carrying and -release capacities could be predictable depending on the level of hydration, a feature that would be of interest in the area of drug design and in vivo delivery, particularly for gene therapy and gene editing applications.

Finally, we note that the similarity between the dehydrated samples and their composition mimics encourages future work not only via the obvious convenience of using mimics in place of dehydrated samples, but also in the conceptual differences between them. While the parameters examined in the experiments above collapsed to one behavior, it is fascinating to consider the directionality of these two sets of samples: the dried samples are moving towards a state of complete dehydration; the mimics are traversing the phase diagram towards a single-phase highly hydrated system. Nonetheless, they behave similarly: while the mixture dries, the coacervate phase acts like a more hydrated entity. The wet sample, depending on the starting composition, could start with a polymer-rich phase in a kinetically trapped state, e.g., with encapsulated RNAs unable to diffuse freely. As the solution dries, the coacervate phase becomes more liquid-like, allowing the RNA molecules to diffuse more freely before further drying traps it again in a solid form. Such progression has been observed before by groups exploring the different morphologies of polyelectrolyte complex coacervates^33,66^, and requires further analysis. The implications on the protocell-behavior of coacervate are intriguing. On one hand they show the striking role of the coacervate-to-total-volume ratio in determining the composition and phase behavior of the compartment. On the other hand, they raise questions as to whether there exists a divergence point in the behaviors among the two types of samples and the reliance of such a point on the composition and hydration state of each component.

## Methods

### Materials

Poly(diallyldimethylammonium chloride), PDADMAC, was obtained from Polysciences, Inc. (molecular weight, MW ~8500 g mol^−1^), and poly(acrylic acid), PAA, was obtained from Sigma–Aldrich (average MW ~1800 g mol^−1^). Sodium chloride (NaCl, ≥99.5%), 4-(2-hydroxyethyl)piperazine-1-ethanesulfonic acid sodium salt (HEPES, ≥99.5%), and magnesium chloride hexahydrate (MgCl_2_.6H_2_O) were all purchased from Sigma–Aldrich. Polymer stock solutions were prepared in water with a final weight percent of around 10–11% for PDADMA and around 5% for PAA. The pH of the stock solutions was adjusted to ~7.5 using sodium hydroxide (NaOH, EMD, ≥97.0%). The oligonucleic acid U15, a uridylic acid 15-mer, was obtained from Integrated DNA Technologies and was labeled on the 5’-end with Alexa Fluor 647. The U15 oligomer was dissolved in nuclease-free water (Ambion), aliquoted, and stored at −20 °C. Micro cover glasses (no. 1.5, 24 × 30 mm, VWR) were used to image samples. Silicon spacers (Electron Microscopy Sciences), with 9 mm internal diameter and 2 mm depth, were sandwiched between two cover glasses for imaging. All polymer solutions and coacervates were prepared using HPLC grade water (Ricca Chemical Company). Unless otherwise stated, coacervate solutions were prepared, dried, and rehydrated in polypropylene microcentrifuge tubes (VWR).

To improve the imaging of coacervate droplets by preventing their coalescence on the surface, the slides were functionalized with oligoethylene glycol moieties to reduce interactions with coacervate components. This was done by incubating them in a 0.3% solution of N-(triethoxysilylpropyl)-o-polyethylene oxide urethane (95%, Gelest, Inc.) in anhydrous toluene (99.8%, EMD Millipore Corporation) for 4 h. The treatment was preceded with a cleaning step that consisted of soaking the slides in a saturated solution of potassium hydroxide (≥85%, Sigma–Aldrich) in isopropanol (90%, Ricca Chemical Company) for 30 min followed by water rinsing and drying at 70 °C. After the oligoethylene glycol functionalization step, the slides were thoroughly washed with toluene, ethanol (190 proof, Koptec), and water, respectively, with a final drying step at 70 °C overnight.

### Coacervate preparation, drying, and addition of U15

Coacervates were prepared by mixing specific volumes of HPLC grade water with the appropriate volumes of MgCl_2_ (0.216 M), HEPES (0.942 M), and NaCl (1.0 M) stock solutions, followed by adding PAA and PDADMA, respectively, to reach the desired final polymer concentrations. All components concentrations are mentioned in the Results section and figures as they were adjusted to suit the goal of each experiment. The concentrations of polyelectrolyte stock solutions were around 0.6–0.8 M (all with respect to the monomer or single charge) depending on the trial, and 1.6–1.8 M in certain turbidity phase diagram experiments that required higher polymer concentrations. The charge ratio was kept at 1:1 by equalizing the final concentrations with respect to the monomers. The addition of the polycation (last added component) caused the solution to become visibly turbid, a sign of coacervation. Solutions were mixed by pipetting gently with a 1000 or 200 µL pipette, depending on the total volume. The tubes were vortexed before and after the addition of the polyanion, then quickly spun (3 s) to collect any liquid on the walls or the cap. After the addition of the polycation, the tubes and their contents were weighed again to obtain the initial weight of the coacervate solution. Samples were then quickly pipette-mixed to uniformly disperse the coacervate, spun down for few seconds, and placed in a preheated heatblock for drying.

Drying of coacervate solutions was performed using a Digital Heatblock (VWR). The microcentrifuge tubes containing the coacervates were placed in the heatblock after preheating it to the chosen temperature (95 °C). The internal temperatures of the block (and tubes) surfaces were verified using a thermometer (Traceable, VWR) equipped with a type K thermocouple. These measurements showed that the temperature at the bottom of the tubes reached the surrounding value of 95 °C after around 2 min and 30 s. All samples were prepared in triplicates and different samples were prepared for each time point (0, 40, 80, 120, 160 min, and a sample that was fully dried for a total time of 4 h 40 min to be used for rehydration)—and all heated simultaneously. The t = 0 samples were prepared at the same time as the others but not heated. The tubes were placed in the block with the lid open to allow water evaporation.

At each time point, a set of triplicates (positioned at different locations of the block to avoid errors from temperature variability) was capped, removed, and allowed to cool down to room temperature before weighing. All empty tubes were weighed initially and after sample preparation, before heating and after heating, using a digital balance (Mettler Toledo) to ensure uniformity of treatment among experiments. The ambient room temperature and humidity were recorded every 5 min during the drying experiments using a Bluetooth Datalogging Temperature and Humidity Meter (TraceableGO, VWR), with no noticeable effect of both parameters on the outcome of drying. As the coacervate would have settled at the end of each drying period (or after leaving the samples for a day or two for triplicate analysis), a 200-µL pipette was used to resuspend it before the addition of the U15 oligomer for confocal microscopy experiments. Measuring the pH of the turbid coacervates or the supernatant solutions was done using a pH meter (SevenExcellence, Mettler Toledo) equipped with an Ultra-Micro-ISM pH electrode (Mettler Toledo) for small-volume samples.

The U15 aliquot was heat-shocked at 95 °C for around 60–90 s, allowed to return to room temperature, and vortexed before the addition of the adequate amount to the resuspended coacervate samples, followed by pipetting to allow even dispersion. The heat shock is performed to disrupt any aggregates and misfolds due to cold storage of the RNA aliquot (at −20 °C). While such artifacts are not expected in simple homo-oligomers, we wanted to avoid any potential interference with the compartmentalization behavior. This order allowed to check the new state of the coacervate and prevent RNA hydrolysis. As the diffusion of the RNA was shown to be slow in some samples (low [polymer], [salt]), the addition of the fluorescently tagged U15 to the coacervate was followed by incubating the solutions in the dark for around 4 h to allow equilibration, and avoid light damage to the fluorophore. This was followed by mixing the samples again with a pipette and placing the desired amount of coacervate (usually 10 µL) on oligoethylene glycol-functionalized slides. After covering the samples with a nontreated slide sandwiching a silicon spacer, they were allowed to sit for at least one hour while other, faster diffusing samples, were imaged. It was apparent that some slow-diffusing droplets had higher U15 concentrations at the beginning which could be caused by the RNA loading the coacervate phase that it encountered first more. Because of the relatively faster diffusion of U15 within all these droplets (see intradroplet FRAP results), no local inhomogeneity (or aggregation) was observed. The incubation steps led to uniform fluorescence in slow-diffusing samples (which otherwise exhibited differing levels of fluorescence among droplets) and were not necessary for samples which showed faster dynamics.

### Turbidity measurements

To establish their phase diagram, the turbidity of coacervate solutions was measured with an Infinite M1000 Pro instrument (Tecan) using the i-control microplate reader software (version 1.11). PDADMA/PAA coacervates were prepared at 1:1 charge ratio with different concentrations of polymers (with respect to monomer concentration) and NaCl. HEPES and MgCl_2_ concentrations were kept at 25 mM and 4.3 mM for all these samples. All solutions were prepared in a 96-well plate (Flat bottom, Nunclon Delta transparent, ThermoFisher) with a total volume of 250 µL each. Multiple reads of absorbance per well were performed and the average was calculated for each sample. After correcting for background absorbance, the percent transmittance was calculated, as (10^Absorbance^ at 500 nm) × 100, followed by the turbidity, as 100 − %Transmittance.

Temperature-dependent turbidity was measured using an OLIS 8453 diode-array UV-visible spectrometer equipped with a Peltier temperature-controlled cell holder where up to six cuvettes containing the solutions could be placed. A SpectralWorks software was used to set up the measurements and temperature. While it was possible to scan the absorbance as the temperature was varied automatically, a decrease in turbidity was observed and ascribed to some evaporation of samples during the long measurement. For that reason, fresh 1:1 PDADMA:PAA coacervate samples were prepared in duplicates for each time point, and allowed to sit at the specific temperature for 10–12 min before the measurement. Coacervate samples prepared in water alone and in buffer (50 mM NaCl, 25 mM HEPES, 4.3 mM MgCl_2_) were measured this way at temperatures that ranged between 5 and 95 °C.

### Zeta potential measurements

The zeta potential of coacervate samples was measured using a Malvern Zetasizer Nano Series (Nano ZS, Malvern Panalytical). Coacervates were prepared with a total volume of 1 mL in the addition order described above, and transferred to a folded capillary zeta cell (Malvern) for the measurement. Data recording and analysis was done on the Zetasizer Software 7.10 from Malvern. A Leica Auto Abbe refractometer was used to obtain the refractive index of the buffer solutions.

### Imaging samples without fluorescence labeling

Transmitted light imaging of samples to support the turbidity analysis of the phase diagram and the initial drying experiments was performed using a Nikon Eclipse TE200 inverted microscope equipped with an Image-Pro Plus 7.0 software. Samples were sandwiched between an oligoethylene glycol-functionalized slide at the bottom and a normal microscope slide on top with a silicon spacer, which were placed on top of a Nikon Plan APO ×100/1.40 oil objective. Images were analyzed using the ImageJ software.

### Partitioning and fluorescence recovery after photobleaching (FRAP)

Imaging of coacervate samples containing fluorescent probes was performed on a Leica TCS SP5 laser scanning confocal inverted microscope. All images were acquired with an HCX PL APO ×63.0/1.40–0.60 oil CS UV objective on a LAS AF software (both from Leica), and data analysis was performed on a LAS AF Lite software (ver. 2.6). While images were acquired at different zoom factors, a zoom of 3.0 was used to measure the fluorescent emission from droplets for partitioning experiments as well as to establish a calibration curve for the Alexa 647-tagged U15. For all imaging experiments, including the calibration curve, the excitation was at 633 nm, the excitation beam splitter was chosen as TD 488/543/633, and the emission bandwidth of the photomultiplier tube was set to 650–695 nm. All partitioning experiments were performed in duplicates or triplicates and 3 images of at least 5 droplets were analyzed for each trial (each result was usually an average of 15 droplets per trial ×2 or 3).

To obtain the partitioning coefficient K (ratio of U15 concentration in coacervate phase/concentration in dilute phase ratio), the samples were centrifuged (Centrifuge 5415 R, Eppendorf) at 13,200 rpm (16,100 × *g*) for 15 min, and around 200 µL of the supernatant was transferred to a new tube for fluorimetry measurements. For samples that were dried further and did not contain enough supernatant volume for the analysis in the 160-µL cuvette, a specific volume of the supernatant was diluted in water and the results were corrected to adjust for the dilution factor. A Fluorolog instrument (Horiba Scientific) was used to measure the fluorescence emission from the supernatant at 665 nm for the Alexa 647-U15 probe. Excitation was at 633 nm, like in the confocal measurements and multiple scans (at least 10) were averaged for each trial to obtain the final results. A calibration curve was established using the same settings, and was used to convert counts to concentration.

FRAP experiments were performed on the same confocal instrument with all the lasers turned on (543, 633 nm, and the Argon laser at 20%). While imaging was still performed using the same settings mentioned above, the bleaching was performed at 100% 458, 476, 488, 514, 543, and 633 nm laser power. Partial regions of droplets (1 µm diameter) or the whole droplet (5–9 µm diameter, depending on the coacervate composition) were selected as regions of interest (ROI). Depending on the experiment, pre-bleaching imaging and bleaching were set to 5 or 10 frames. In fast diffusing samples, bidirectional scanning was performed to capture as much data as possible at the beginning of the post-bleach imaging. Post-bleach time and frames were adjusted to match the diffusion within each sample. All experiments were performed in duplicates or triplicates (with measurements of at least nine internal regions for partial-droplet analysis and six droplets for whole-droplet analysis). When experiments that required larger amounts of U15 were performed in duplicates, the number of droplets and internal regions were increased to match the same number of trials done in triplicates.

Because of loss of fluorescence during multi-frame post-bleach measurements, the fluorescence was normalized to obtain the *F*_*N*_(*t*) as was described before^47,64^, using:

1 $$F_N\left( t \right) = \frac{{\left[ {S_{(t)} - B_{(t)}} \right]\left[ {R_{(0)} - B_{\left( 0 \right)}} \right]}}{{\left[ {R_{(t)} - B_{(t)}} \right]\left[ {S_{(0)} - B_{\left( 0 \right)}} \right]}}$$

where *S*_(*t*)_ was the average measured fluorescence recovery postbleaching of the bleached ROI, *R*_(*t*)_ the average fluorescence intensity of a reference ROI that was either a similarly-sized whole droplet in the same frame for whole-droplet experiments or a 1-µm nonbleached region within the same droplet for intradroplet experiments, *B*_(*t*)_ the average background fluorescence of a randomly chosen region postbleaching. As for background fluorescence, *B*_(0)_ was the background reference obtained as the average of five images measured separately with all the lasers turned off, *S*_(0)_ was the average of the prebleach fluorescence for the specific bleached ROI, and *R*_(0)_ was the average prebleach fluorescence of the reference ROI.

Data fitting was performed using an OriginPro 8.5 software according to the following equation:

2 $$F_N\left( t \right) = A\left[ {1 - \exp \left( { - \frac{t}{\tau }} \right)} \right] + C$$

where *A* is the mobile fraction of the fluorescence probe, *C* the y-intercept of the *F*_*N*_(*t*) fluorescence intensity recovery curve, and *τ* the fluorescence recovery time constant specific to each experiment, which could be used to obtain the halftime (*τ*_1/2_) of fluorescence recovery:

3 $$\tau _{1/2} = \tau {\mathrm{ln}}(2)$$

The halftime can then be used, along with the radius (*r*) of the specific bleached ROI, to obtain the apparent 2D diffusion coefficient, according to^67^:

4 $$D_{{\mathrm{app}}} = \frac{{0.88\,r^2}}{{4\tau _{1/2}}}$$

where 0.88 is a constant used for calculating diffusion for circular beams.

### Small-angle X-ray scattering (SAXS)

SAXS experiments were conducted at beamline 12-ID-B of the Advanced Photon Source at Argonne National Laboratory (Lemont, IL USA). Measurements were collected with 14 keV X-rays at a sample-to-detector distance of 4 m (*q*-range = 0.002–0.5 Å^−1^, equivalent to 1.2–310 nm length scale, with displayed region of interest corresponding to 1.25–63 nm) at 25 °C. The prepared coacervate samples were transferred into 2 mm quartz capillaries (Charles Supper Company, Natick, MA USA) and centrifuged at 5000 × *g* for 10 min, resulting in two coexisting phases. The capillaries were sealed with epoxy glue and stored at room temperature for at least 24 h prior to measurement. During the SAXS experiments, the capillaries were positioned in holding racks so that the coacervate phase was in the path of the beam to obtain scattering from the centrifuged coacervate. After collecting each sample, the path of the beam was moved vertically to check the supernatant phase against the respective aqueous background. The signal from this background, which is mostly water, was later subtracted from the coacervate phase signal. The exposure time of the samples was limited to 0.1 s. All two-dimensional data were converted to one-dimensional intensity profiles and processed using the Irena package^62^ for Igor Pro.

### Statistical analysis

The means and standard deviations are shown within the figures with additional details in the captions. We employed a single-factor ANOVA test to measure the differences between cycled samples and mimics samples. Where the results would not allow such a test, a two-tailed *t*-test was used. The results of these tests are available in the Supplementary Note 7.

## Supplementary information

Supplementary Information

## Data Availability

All the data are available within the paper and its Supplementary Information file. Relevant data can be provided by the corresponding author upon request.
