# Supplementary material for: Impact of wet-dry cycling on the phase behavior and compartmentalization properties of complex coacervates
Source: Nat Commun. 2020 Oct 27;11:5423. doi: 10.1038/s41467-020-19184-z (PMC7592044; doi:10.1038/s41467-020-19184-z)
Supplement: Supplementary file 1 — Supplementary Information [file 41467_2020_19184_MOESM1_ESM.pdf]

Supplementary Information

Impact of Wet-Dry Cycling on the Phase Behavior and Compartmentalization Properties of Complex Coacervates

Hadi M. Fares[1,2], Alexander E. Marras[3,4,†], Jeffrey M. Ting[3,4,†], Matthew V. Tirrell[3,4], and Christine D. Keating[1]*

[1] Department of Chemistry, The Pennsylvania State University, University Park, PA 16802, USA;
[2] NASA Postdoctoral Program, Universities Space Research Association, Columbia, MD 21046, USA;
[3] Pritzker School of Molecular Engineering, University of Chicago, Chicago, IL 60637, USA;
[4] Center for Molecular Engineering and Materials Science Division, Argonne National Laboratory, Lemont, IL 60439, USA.

Supplementary Notes, Discussion, and References:

Supplementary Note 1: Choosing the coacervate system 3

Supplementary Note 2: Troubleshooting the drying step and quantification of the dried mixture (Supplementary Figures 1, 2, 3, 4) 4

Supplementary Note 3: Troubleshooting the buffer and $MgCl_2$ concentrations (Supplementary Figures 5 and 6) 6

Supplementary Note 4: Example weight percent of starting solution mixture, and dried samples (Supplementary Figure 10) 7

Supplementary Note 5: Zeta potential measurements 22

Supplementary Note 6: Troubleshooting the whole-droplet exchange experiment with equal [U15] 27

Supplementary Discussion 1: Discussion of the movements on the phase diagram 30

Supplementary Note 7: Statistical analysis for Figures 4 and 5 33

Supplementary References 56

Supplementary Figures and Tables

Supplementary Figure 1....................................8
Supplementary Figure 2....................................9
Supplementary Figure 3....................................9
Supplementary Figure 4..................................10
Supplementary Figure 5..................................11
Supplementary Figure 6..................................12
Supplementary Figure 7..................................13
Supplementary Figure 8..................................14
Supplementary Figure 9..................................15
Supplementary Figure 10................................16
Supplementary Figure 11................................17
Supplementary Figure 12................................18
Supplementary Figure 13................................18
Supplementary Figure 14................................19
Supplementary Table 1...................................20
Supplementary Figure 15................................21
Supplementary Figure 16................................21
Supplementary Table 2...................................23
Supplementary Table 3...................................25
Supplementary Figure 17................................27
Supplementary Table 4...................................28
Supplementary Figure 18................................30
Supplementary Figure 19. 32
Supplementary Figure 20. 32
Supplementary Figure 21. 34
Supplementary Table 5..................................35
Supplementary Table 6..................................37
Supplementary Table 7..................................39
Supplementary Figure 22. 39
Supplementary Figure 23. 40
Supplementary Figure 24. 41
Supplementary Table 8..................................42
Supplementary Table 9..................................43
Supplementary Figure 25. 44
Supplementary Table 10................................44
Supplementary Figure 26. 45
Supplementary Table 11................................45
Supplementary Figure 27. 46
Supplementary Figure 28. 47
Supplementary Table 12................................48
Supplementary Table 13................................50
Supplementary Table 14................................52
Supplementary Table 15................................54

Supplementary Note 1: Choosing the coacervate system

Many coacervate forming polymer pairs have been used to demonstrate the potential of the coacervate polymer-rich phase to exhibit protocell or artificial-cell like behaviors. While choosing the type of polymers here, the focus was to pick a pair with proven functional versatility but also one that was amply available to support the multiple troubleshooting steps of drying a coacervate. The poly(diallyldimethylammonium) PDADMA/poly(acrylic acid) PAA pair matched these requirements. PDADMA/PAA has also been used in protocell research,[1-3] with the polycation specifically taking prominence in numerous studies[3-6] and shown to promote RNA polymerization.[7] We chose polydisperse shorter chains, with a polycation molecular weight around 8500 and that of the polyanion ca. 1800 g mol^{-1}, to ensure the prebiotic relevance of the molecular weight (around 53 and 25 units, respectively). The polydisperse aspect of these macromolecules means that some longer chains, but also shorter ones, are expected to exist in the mixture. With an approximate dispersity ($Đ$) of 1.5 – 2.0 for polydisperse molecules, we can estimate the range of repeat units using the standard deviation ($\sigma = M_n (Đ - 1)^{1/2}$) of the molar mass distribution.[8] The resulting standard deviation ranges of PAA and PDADMA, respectively, are around 1270 - 1800 g mol^{-1} (21 units) and 6010 - 8500 g mol^{-1} (45 units). This provides an estimate of 4 - 46 units for PAA and 8 - 98 units for PDADMA. Studies on wet-dry cycling-driven polymerization have shown that oligopeptides of up to 11 units,[9, 10] nucleic acids of up to 150 base pairs,[11] at least 10 monomeric units of malic acid oligomers,[12] and up to 14-mer depsipeptide-oligomers,[13] can be produced through dehydration-hydration cycles. We also picked functional groups, carboxylate and ammonium, that are building blocks widely available in nature and involved in numerous prebiotic syntheses.[14] The monomer of the polycation has also been detected, among a number of other amine groups, in carbonaceous chondrites.[15]

The presence of salts was also required to fit the suggested ionic content of geothermal fields or ponds.[16] Sodium chloride was chosen as the main salt and magnesium chloride was added in anticipation of introducing ribonucleic acids, as it has been shown to enhance their function by possibly organizing water molecules around the RNA and interacting with the phosphate oxygens.[17-19] The starting concentrations of the main ions were around 0.4 – 0.9 mM for Mg^{2+} and between 1 – 50 mM for NaCl. The choice of these values is explained further in Figure 1 and Note 3 below. These concentrations are equivalent to around 9.6 mg/L – 21.6 mg/L (9,600 – 21,600 ppb) of Mg^{2+} and 23 mg/L – 1,150 mg/L (23,000 – 1,150,000 ppb) of Na^{+} – ranges that fall mostly within, or on a similar order of magnitude, of those present in the waters of various inland ponds as reported by Mulkidjanian et al.[16] While pH could be low in these ponds, we added a buffer, HEPES, in our experiments to ensure that the pH would not vary, placing the emphasis on the change in hydration level and the accompanying concentration effect. The pH was tested in the experiments below.

The water of primordial oceans is thought to have been more concentrated in sodium and magnesium (~0.4 M and ~0.01 M, respectively)[16]. Such locations are not, thus, relevant to the wet-dry cycles studied here. Nonetheless, the literature demonstrates that coacervates can have stronger interaction strengths, depending on the chemistry of their constituents, which would translate into improved salt resistance.[20] Such stronger-interacting coacervates could be, in the future, studied in the context of wet-dry cycles occurring in tide pools or ocean water.

Supplementary Note 2: Troubleshooting the drying step and quantification of the dried mixture (Supplementary Figures 1, 2, 3, 4)

For drying, the goal was to find a manually controllable procedure that would allow the simultaneous drying of multiple samples. While initial attempts included using an oil bath with

temperature control, drying directly on a hot plate, and immersing tubes in a heated water bath, the most fitting process was to use a heat block. Empty tubes and samples were weighed before and after each step. A thermometer equipped with a thermocouple was used to measure the temperature of the samples and the heater. A separate meter recorded the ambient humidity and temperature which were not found to impact the drying rate and final volumes. Supplementary Figures 1 – 3 show a series of troubleshooting experiments that were used to validate the quantification of the tubes content and the drying rates (see Methods section for more details).

A series of drying trials was performed to choose the best method to calculate the coacervate content (Supplementary Figure 1). The drying rate of a coacervate suspension prepared from the same charge concentration of polymers in different solvents was found to be identical to the evaporation rate of water (Supplementary Figure 1a). Two different quantification techniques were used to obtain the volume of the content (Supplementary Figure 1b). The first relied on visually comparing the content of the tubes with a calibration curve made with different volumes of water, where the content was quantified using the ImageJ software (Supplementary Figure 2). The second was based on converting the net weight of the tube content into volume using the density of water at room temperature. Both gave identical volumes, and the weight technique was adopted since it was more straightforward. Finally, the volumes of different coacervate compositions (with different salt and polymer concentrations) were compared to that of water at the same time points and were found to be identical which showed, experimentally, that the concentrations are low enough for a density of 1 to still be valid for weight-to-volume conversions (Supplementary Figure 1c).

The evaporation rate was shown to decrease from around 4 to 1.8 µL min^{-1} over the course of a range of time points where the volume decreased by a factor of ~ 10 (from ~ 500 to ~ 50 µL). This

could be attributed to the conical shape of the tube which caused a reduction in the surface area of evaporation with time (Supplementary Figure 3).

The effect of temperature on the PDADMA/PAA coacervate was evaluated in the presence of water only as well as in the presence of buffer (50 mM NaCl, 25 mM HEPES, and 4.3 mM $MgCl_2$) in the range of 0 – 95 °C. This was done by measuring the turbidity after holding the coacervate at the chosen temperature for 10 min. As it can be seen from Supplementary Figure 4, the turbidity did not change across this range of temperatures. In addition to turbidity, further measurements performed in this study support our assertion that temperature does not seem to affect the coacervate. These are: 1) volume measurements (Figures 5, Supplementary Figures 22, 23), which show that the volume ratio is consistent between dried samples (at high temperature) and non-dried samples; 2) weight measurements (Figure 4), which show that the same weight of the dry material is regained after multiple wet-dry cycles at high temperature.

Supplementary Note 3: Troubleshooting the buffer and $MgCl_2$ concentrations (Supplementary Figures 5 and 6)

The compositions and drying procedure were chosen as to not lead to a HEPES and $MgCl_2$ concentrations that surpassed 25 mM and 5 mM, respectively – both within the high turbidity range observed in control experiments (Supplementary Figure 5, Supplementary Information). The starting concentration of 2.5 mM HEPES ensured that the pH would not change significantly as the solutions became more concentrated. Supplementary Figure 6 demonstrates this outcome with pH measurements of the turbid coacervate (entire coacervate solution, Supplementary Figure 6a) and the dilute phase or supernatant (Supplementary Figure 6b). Lower HEPES concentrations caused the pH to decrease by more than one unit, while the chosen starting concentration of 2.5 mM limited this decline to around 0.25 unit.

Supplementary Note 4: Example weight percent of starting solution mixture, and dried samples (Supplementary Figure 10)

An example of the weight/weight percentages of the materials in solution are as follows: PDADMA 0.081% (0.81 g/L), PAA 0.036% (0.36 g/L), NaCl 0.029% (0.29 g/L), $MgCl_2$ 0.0041% (0.041 g/L), HEPES 0.060% (0.595 g/L).

Different compositions of coacervates were imaged after drying 100 µL solutions on slides placed directly on a hot plate (Supplementary Figure 10). When the original sample was prepared in a buffer mixture similar to the one used in the rest of the study, deposits of periodic structures were observed which could be the result of crystallization of salts (Supplementary Figure 10a). When no salt or buffers were added to the original sample, the dried images revealed the "remains" of individual droplets (Supplementary Figure 10b). These are likely the residues of a coacervate phase that coalesced before/during drying. The phase diagram shows that droplets cannot resist high salt concentrations and should dissolve, which would explain why no sign of the coacervate was observed in Supplementary Figure 10a. We then tried to dry samples prepared in the presence of NaCl alone. The dried structures appeared smaller than in Supplementary Figure 10b and evidence of salt crystallization was observed (Supplementary Figure 10c). Drying in the presence of salt, but on an oligoethylene glycol-functionalized slide, led to the coacervate forming a gel-like film that seemed to preserve the shape of droplets (Supplementary Figure 10c – right, marked with asterisk).

The heterogeneity of these observations with coacervates dried on a flat surface is fascinating; we imagine the results would be even more complex on a rocky surface. Further analyses of dry and near-dry coacervates are needed to elucidate the mechanisms behind these observations.

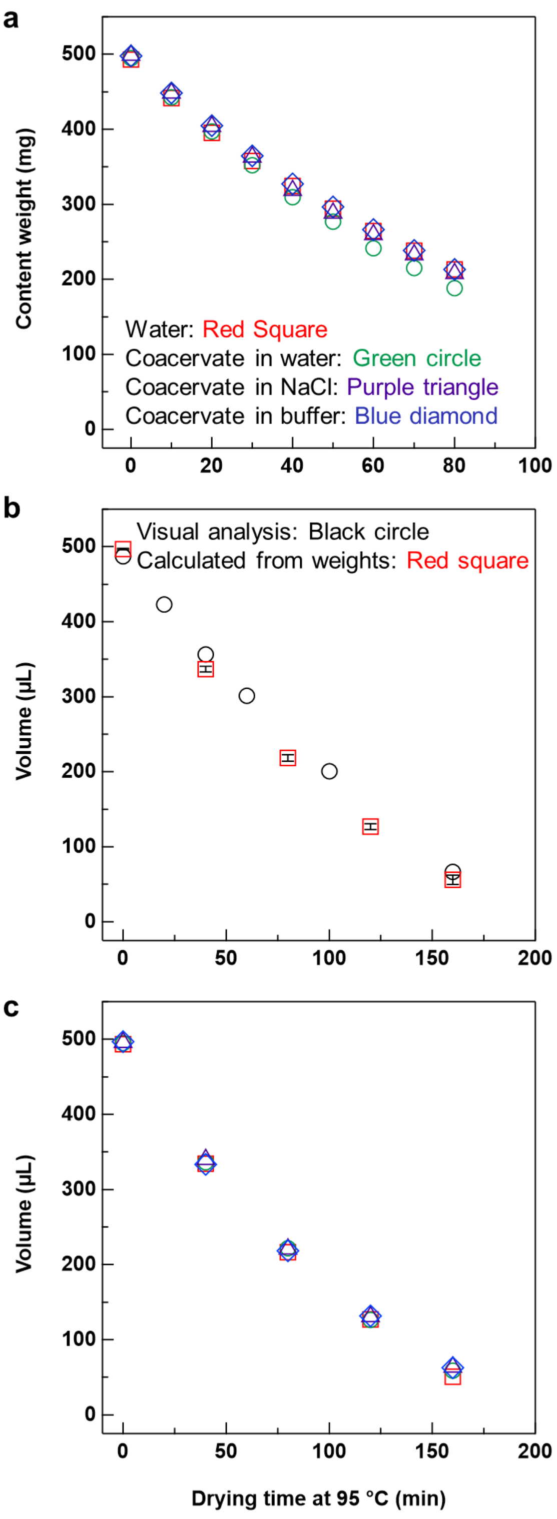

Supplementary Figure 1. Drying and quantification of coacervate content.

In (a), the drying rates of 1:1 PDADMA:PAA coacervates prepared with a fixed charge concentration of each polymer of 15 mM in water, in 50 mM NaCl, and in buffer (50 mM NaCl, 25 mM HEPES, 4.3 mM $MgCl_2$) were compared with the drying rate of water.

In (b), two methods to estimate the volume of a coacervate solution were compared: estimation of the volume by visual analysis using a calibration curve of different water volumes (see below) and estimation of the volume through dividing the weight by the density of water at the weighing temperature (room temperature ~ 23 °C). The content is 1:1 PDADMA:PAA prepared at 5 mM charge concentration, with 5 mM NaCl, 2.5 mM HEPES, and 0.43 mM $MgCl_2$. Error bars represent standard deviations obtained from 3 trials.

In (c), the volumes of different coacervate compositions obtained from the weights are compared at the same time points. The charge concentration is shown in the legend (as "Pol"), as well as the salt concentration. All compositions contained 2.5 mM HEPES and 0.43 mM $MgCl_2$.

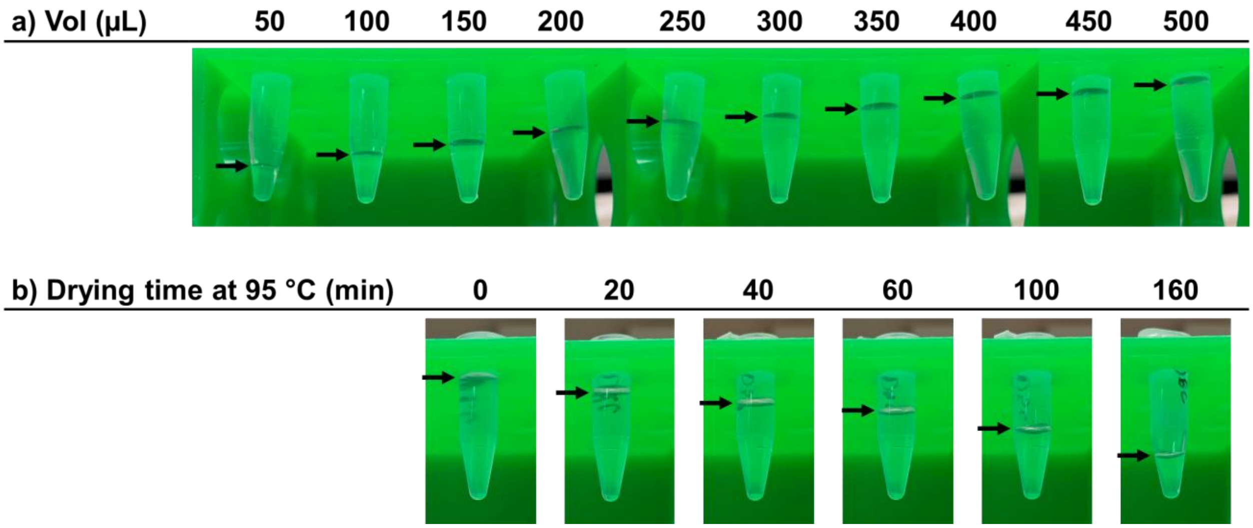

Supplementary Figure 2. Visual comparison of volumes used in Supplementary Figure 1b. a) The visual calibration curve used to estimate the volumes in (b) with the ImageJ software.

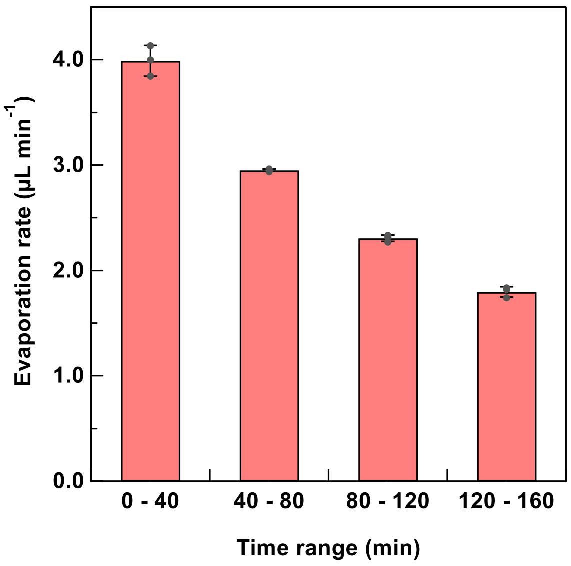

Supplementary Figure 3. Change of evaporation rate over the different time ranges. The evaporation rate was calculated as the difference of the volumes between two time points over the duration of time in minutes. The means and errors are obtained from 3 independent trials consisting each of triplicates.

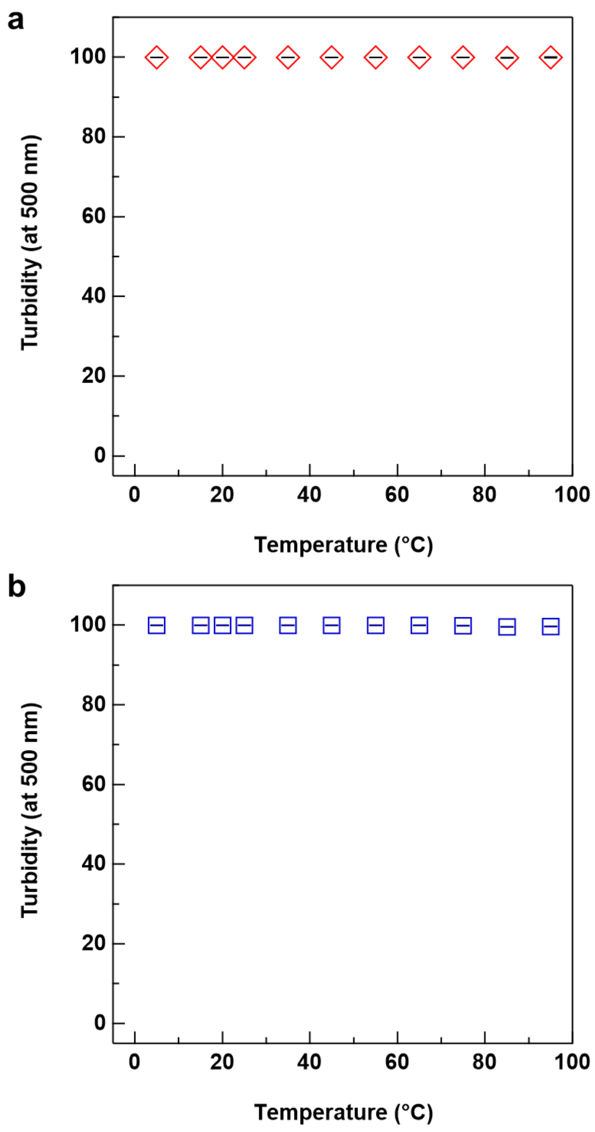

Supplementary Figure 4. Turbidity (100 − %Transmittance at 500 nm) of 1:1 PDADMA:PAA coacervates prepared in water (a) and in buffer (b). Buffer is 50 mM NaCl, 25 mM HEPES, and 4.3 mM $MgCl_2$. The concentration of each polymer in both experiments was 15 mM with respect to the monomer. Means and standard deviations are obtained from 2 samples for each measurement, with individual data points too close to distinguish.

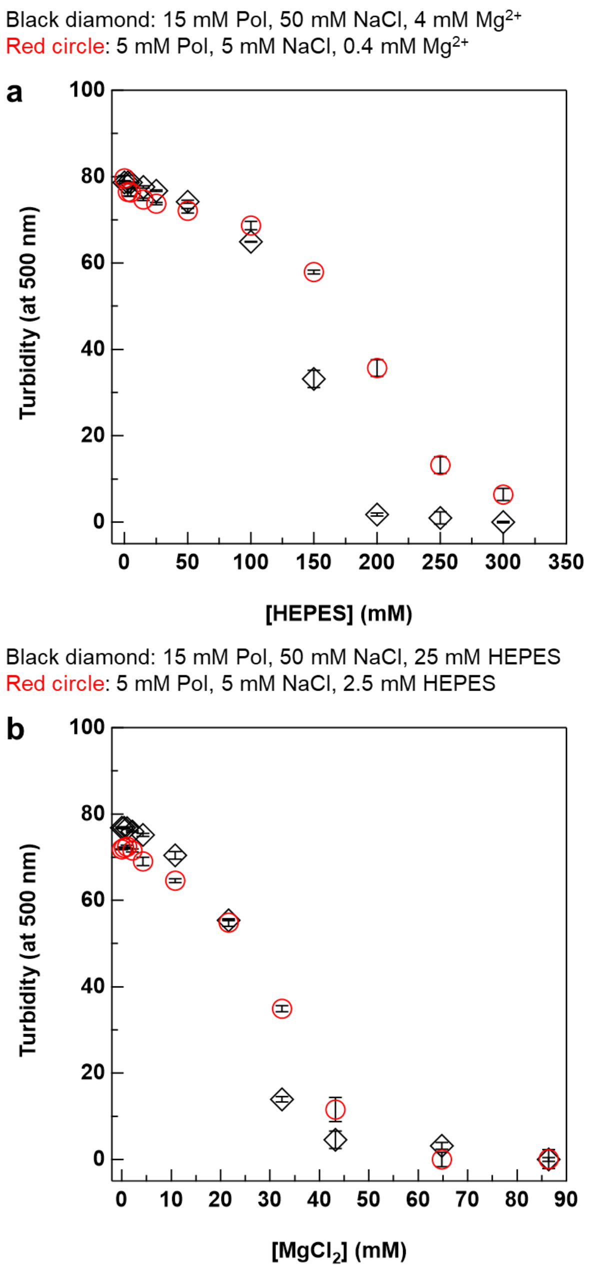

Supplementary Figure 5. Turbidity (100 − %Transmittance at 500 nm) for two different compositions of the 1:1 PDADMA:PAA coacervates (high and low [polymer], [NaCl], and [HEPES] or [$MgCl_2$]) with a range of a) [HEPES] and b) [$MgCl_2$] values. Means and standard deviations are obtained from 2 samples.

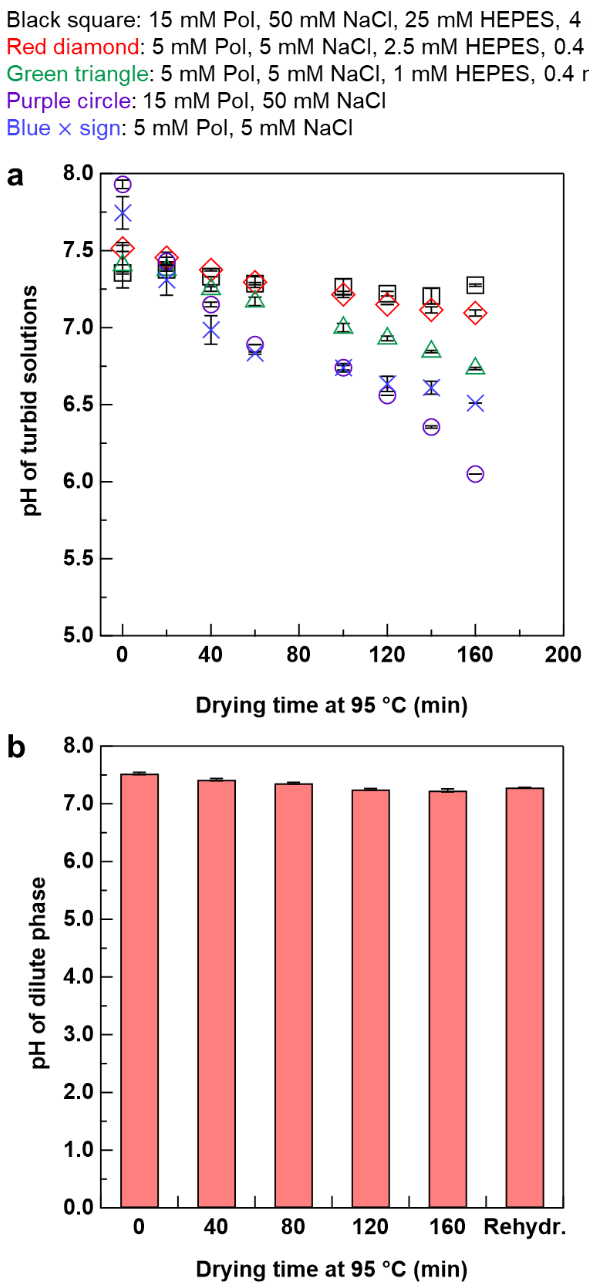

Supplementary Figure 6. a) pH values of the turbid 1:1 PDADMA:PAA coacervate solutions while drying at 95 °C. Different compositions with or without HEPES and $MgCl_2$ were tested, with an initial 2.5 mM HEPES appearing to be adequate for the preservation of the pH as the solution loses water. Means and standard deviations are obtained from 2 samples. b) pH of the dilute phase of a PDADMA/PAA coacervate with 5 mM charge (or polymer with respect to monomer concentration) and NaCl, 2.5 mM HEPES, and 0.43 mM $MgCl_2$. Averages and standard deviations are obtained from 3 different samples.

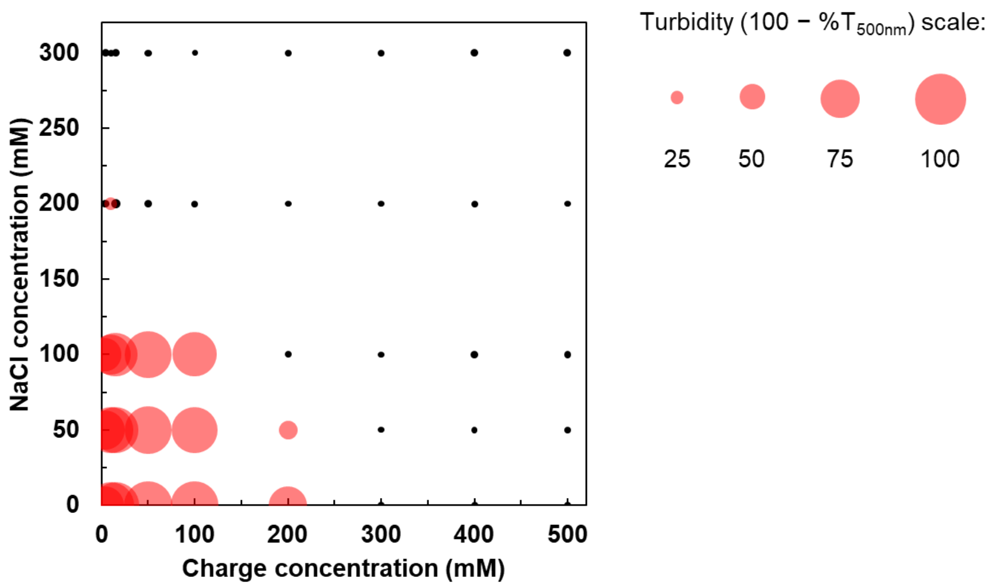

Supplementary Figure 7. Turbidity values (100 − %Transmittance at 500 nm) for PDADMA/PAA complex coacervates prepared at different charge concentrations ([charge] is in 1:1 ratio, as calculated with respect to the concentration of the repeat units) and salt concentrations. All solutions contained 25 mM HEPES and 4.3 mM $MgCl_2$. Black markers represent turbidity below a cutoff of 20, a value that was chosen based on inspection of microscopy images. The width of the circles is proportional to the turbidity values.

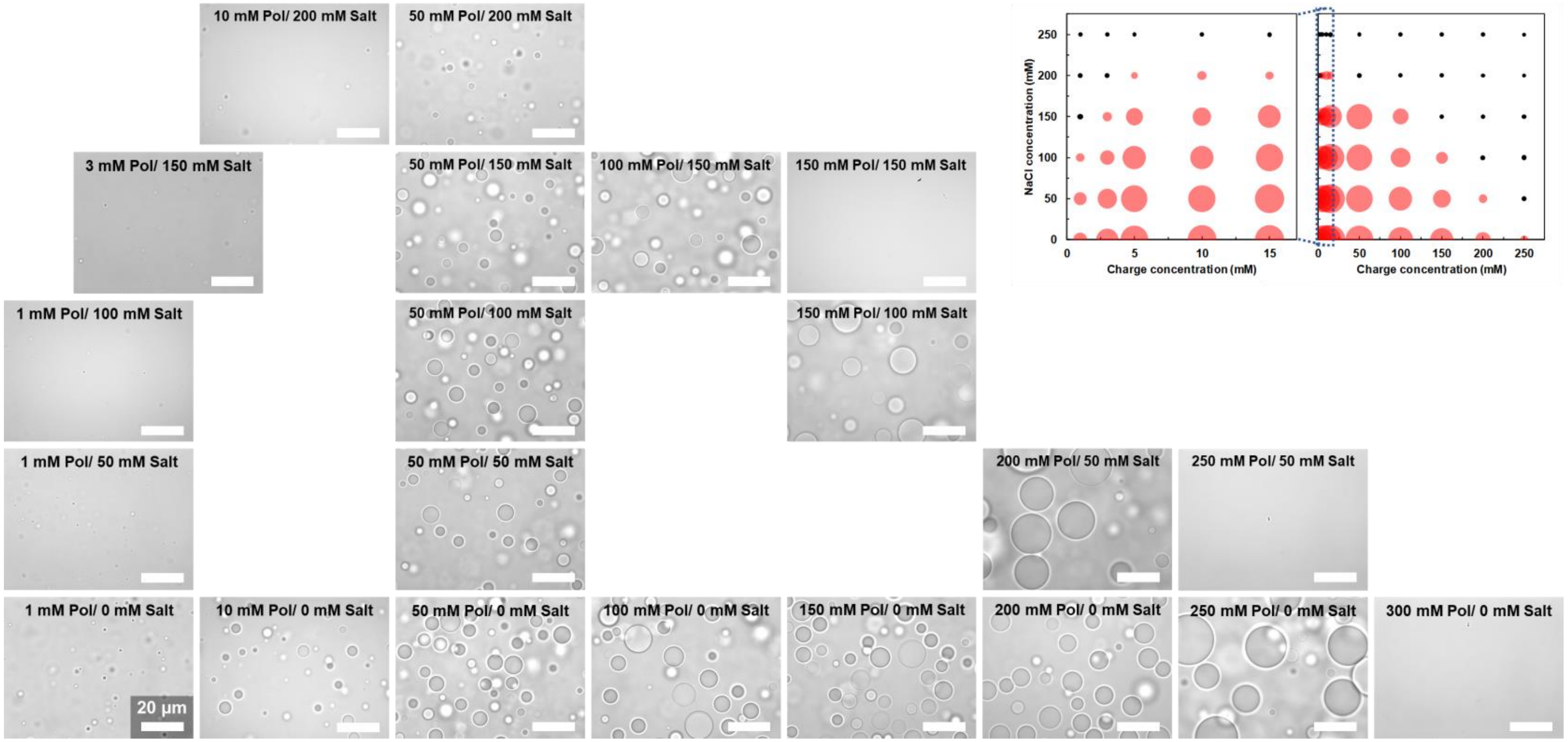

Supplementary Figure 8. Microscopy images of PDADMA/PAA complex coacervates with different salt and charge concentrations. All solutions contained 25 mM HEPES and 4.3 mM $MgCl_2$. All scale bars are 20 µm. The turbidity phase diagram is shown at the top right side for reference.

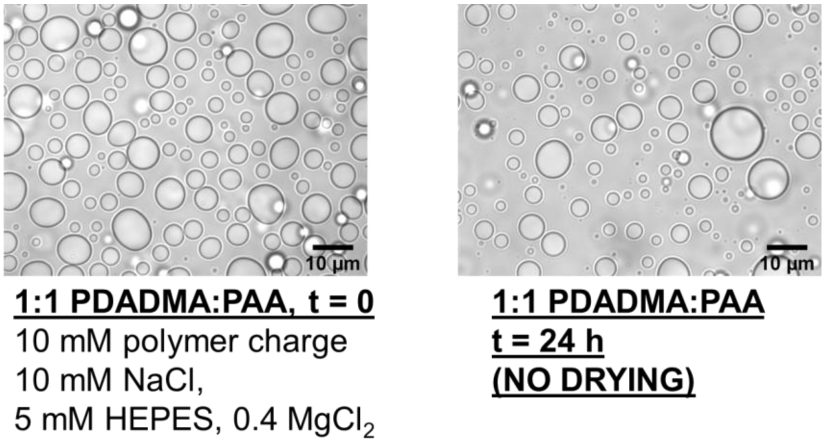

Supplementary Figure 9. 1:1 PDADMA/PAA complex coacervate as prepared (t = 0, left) and after allowing it to rest, without drying, for 24 hours. After 24 hours, it was resuspended, and imaged on an oligoethylene glycol-functionalized slide similar to the one at t = 0.

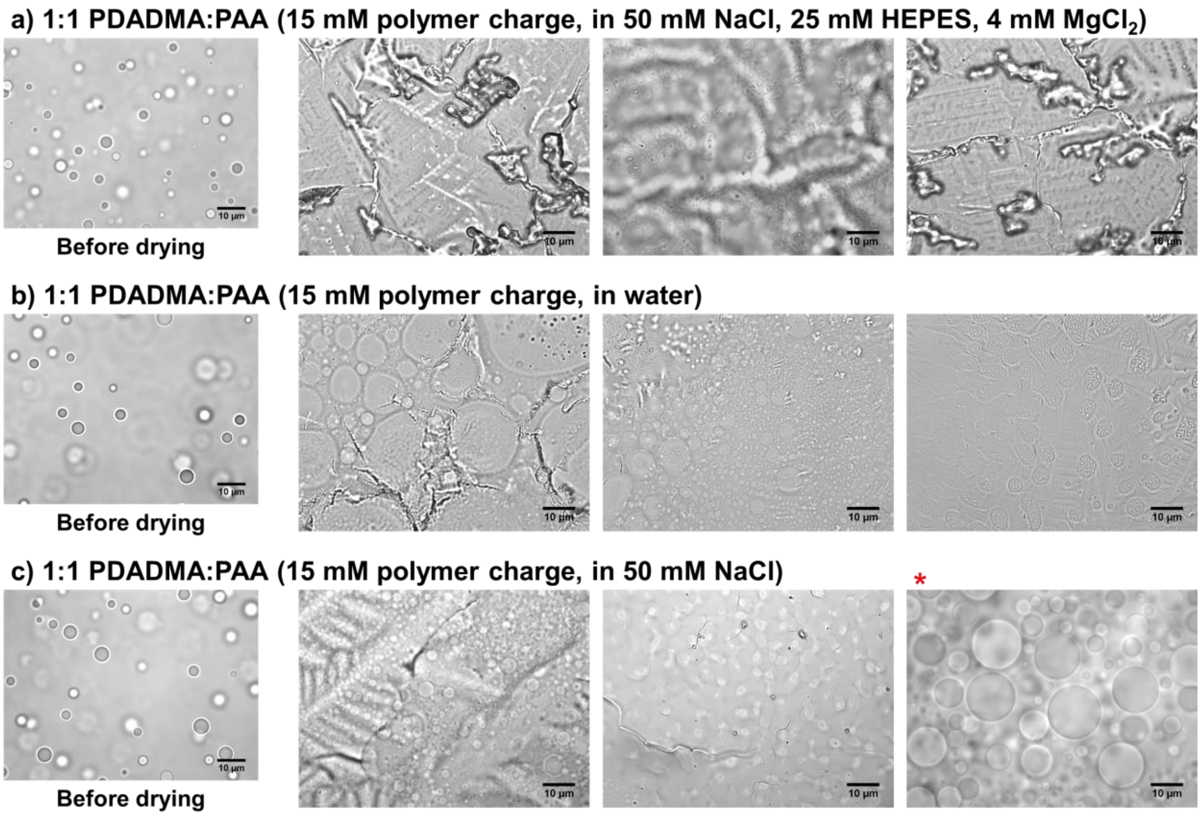

Supplementary Figure 10. Fully dried 1:1 PDADMA:PAA complex coacervate samples on microscope slides. Drying was performed directly on slides at 95 °C and the deposited mixture was imaged before and after drying, when no more liquid could be discerned with the naked eye. Coacervates were initially prepared at 15 mM charge concentration each (with respect to the monomer units) in a) 50 mM NaCl, 25 mM HEPES, and 4 mM $MgCl_2$, b) water, and c) 50 mM NaCl. Panels show the coacervate before drying (left image) and 3 different locations on the same slide after drying (on the right, except c). In c) the first two images after drying are from different locations on the same slide while the third one (on the right, marked with an asterisk *) is from a different sample prepared on an oligoethylene glycol-functionalized slide. All scale bars are 10 µm.

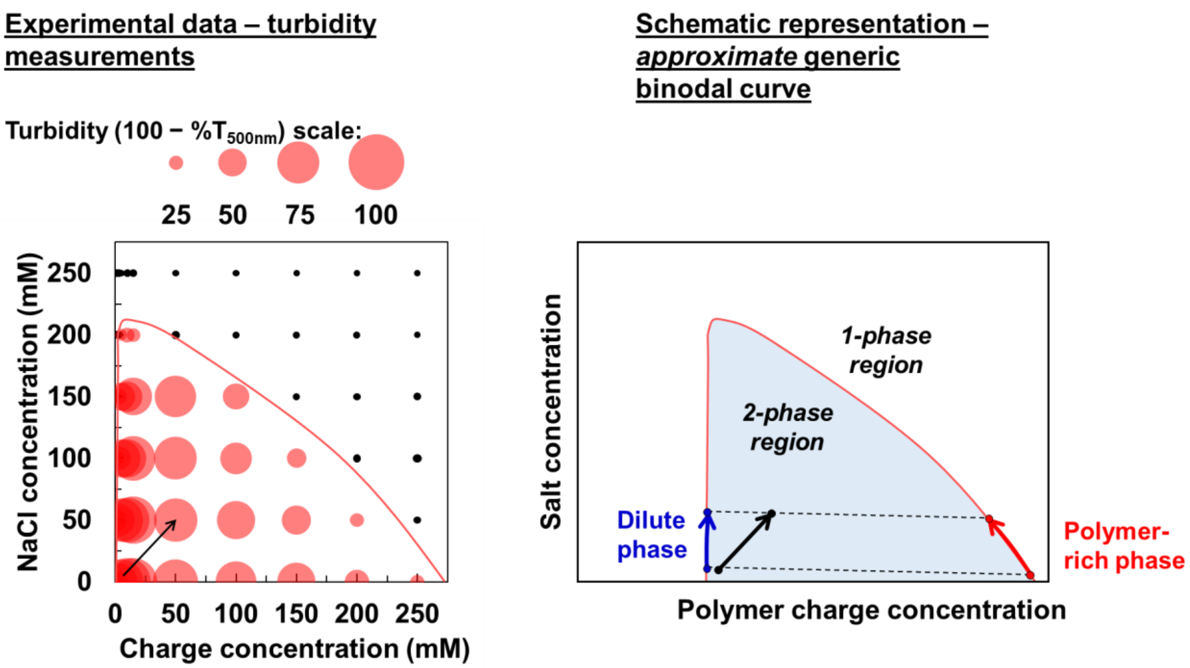

Supplementary Figure 11. Experimental data (left) showing the turbidity phase diagram and schematic representation (right) showing a simplified representation of an approximate binodal curve and tie-lines that could describe the drying-induced movement on the phase diagram from the separate perspectives of the dilute phase and the polymer-rich phase which constitute the overall sample, located experimentally using turbidity measurements. The red line on the turbidity diagram (left) is used to define the *approximate* binodal or phase coexistence curve between the two-phase and one-phase regions.

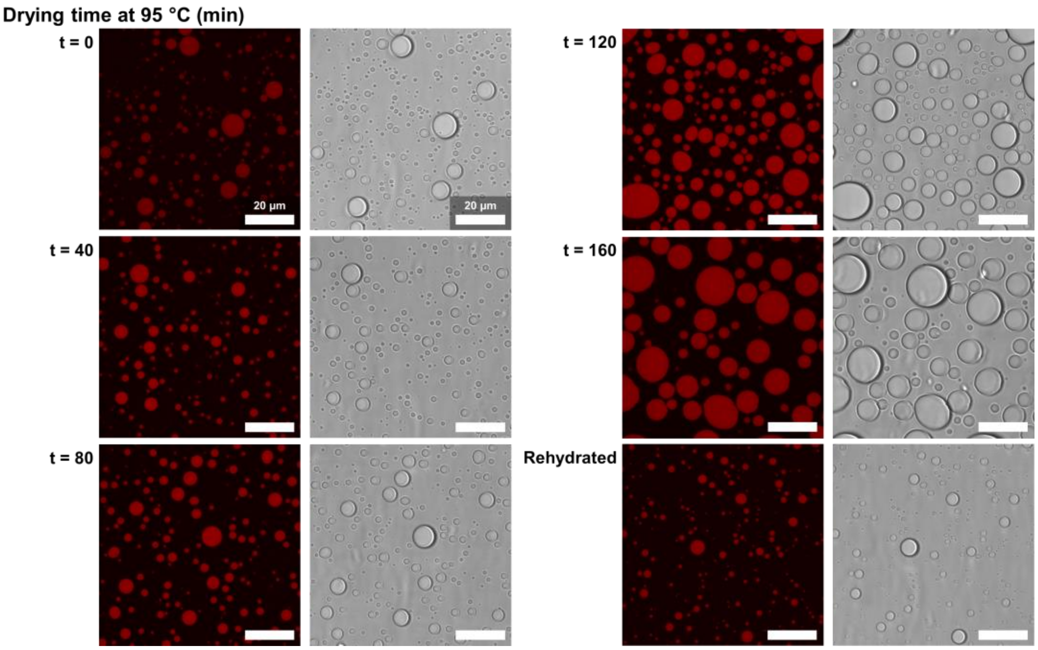

Supplementary Figure 12. Confocal microscope images of Alexa 647-U15 partitioned within 1:1 PDADMA:PAA coacervates at different time points of drying at 95 °C. The initial concentrations added to all samples in these trials were the following (shown as time in min/ concentration in µM): 0/ 0.02, 40/ 0.03, 80/ 0.05, 120/ 0.08, 160/ 0.18, rehydrated sample/ 0.02. The concentrations are chosen so that the same number of moles of U15 is added to each sample in this experiment. All scale bars are 20 µm. Left panels are false colored fluorescence and right panels are differential interference contrast, DIC, images.

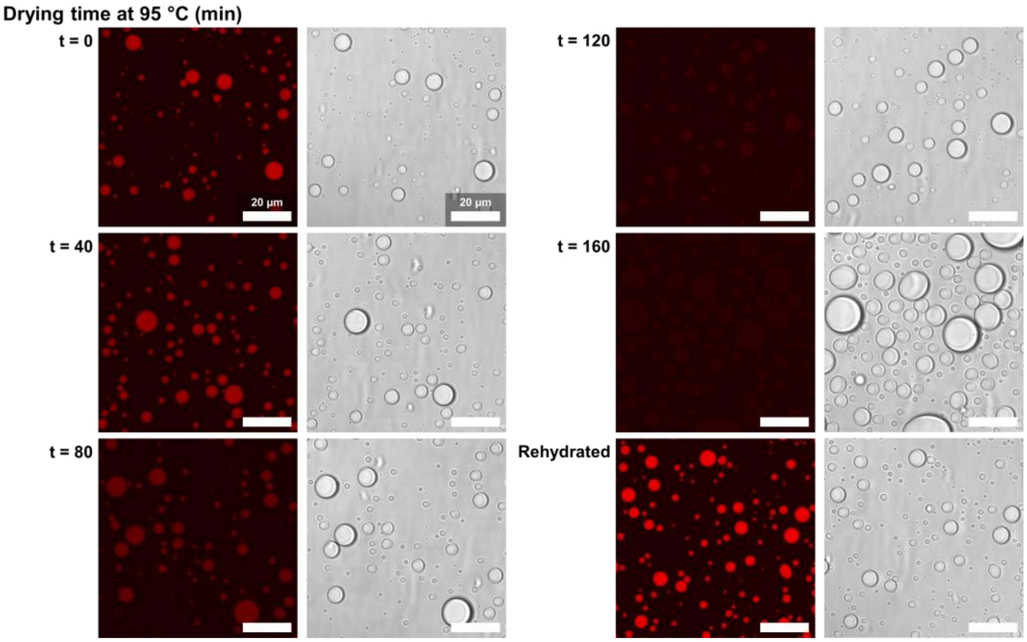

Supplementary Figure 13. Confocal microscope images of Alexa 647-U15 partitioned within 1:1 PDADMA:PAA coacervates at different time points of drying at 95 °C. The initial concentration added to all samples is a constant 0.02 µM. All scale bars are 20 µm. Left panels are false colored fluorescence and right panels are DIC.

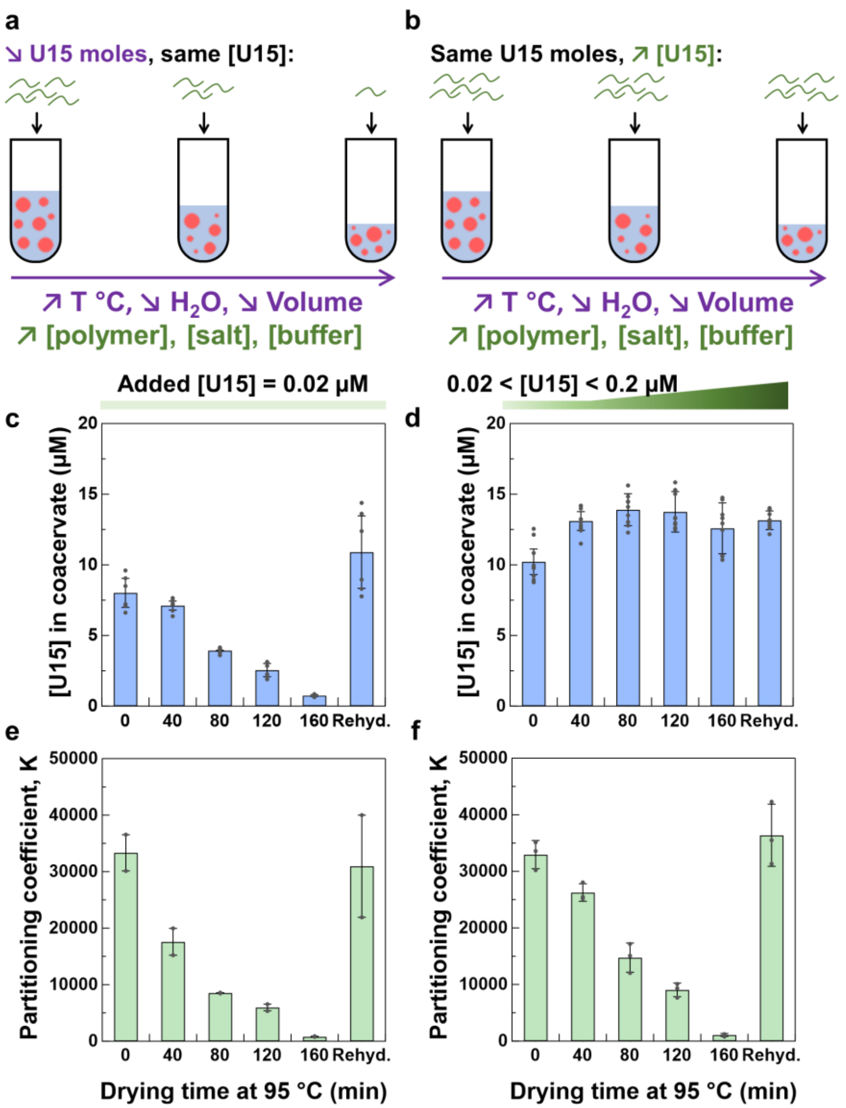

Supplementary Figure 14. U15 partitioning during PDADMA/PAA coacervate suspension drying. To understand the partitioning of U15 within the coacervate, different amounts were added in two experiments. a) In the first, the amount was decreased to match the trend in volume, thus the U15 concentration was constant at 0.02 µM. b) In the second, the amount was kept constant, thus the U15 concentration increased in the same way as all the other components in solution. The addition of U15 was performed after each drying step was completed and the solutions returned to room temperature, in both experiments, to check the new state of the coacervate and to avoid RNA hydrolysis. c and d) Concentrations of U15 within the coacervate after addition of the Alexa 647-tagged probe in a constant initial concentration and in increasing concentrations, respectively. e) and f) show the partitioning coefficient, calculated as $[U15]_{\text{coacervate phase}}/[U15]_{\text{dilute phase}}$, in both experiments. The green bars above (c) and (d) are symbolic representations of total [U15] throughout the experiment. c) and e) include means and standard deviations from 2 samples, each consisting of an analysis of 15 droplets over 3 confocal images. d) and f) include means and standard deviations from 3 samples, with 15 droplets over 3 confocal images per sample. The individual data points in c) and d) show the averages of images – and not of all the individual droplets within each image. The individual data points in e) and f) are the results of dividing the averages of data points in c) and d) by the dilute phases [U15] values, obtained using fluorimetry measurements.

Supplementary Table 1. Concentrations of U15 in the dilute phase in the drying experiment where the total concentrations of U15 increased in the mixture (Figure 2c, d, Supplementary Figure 14b) as measured by fluorimetry. All values are in nM.

	$[U15]_{dilute\ phase}$ (nM)				
**Drying time (min)**	**Trial 1**	**Trial 2**	**Trial 3**	**Average**	**Standard deviation**
**0**	0.3	0.3	0.3	0.3	0.0
**40**	0.5	0.5	0.5	0.5	0.0
**80**	1.1	0.9	0.9	1.0	0.1
**120**	1.4	1.7	1.5	1.5	0.2
**160**	10.8	13.2	11.6	11.9	1.2
**Rehydrated**	0.4	0.4	0.3	0.4	0.0

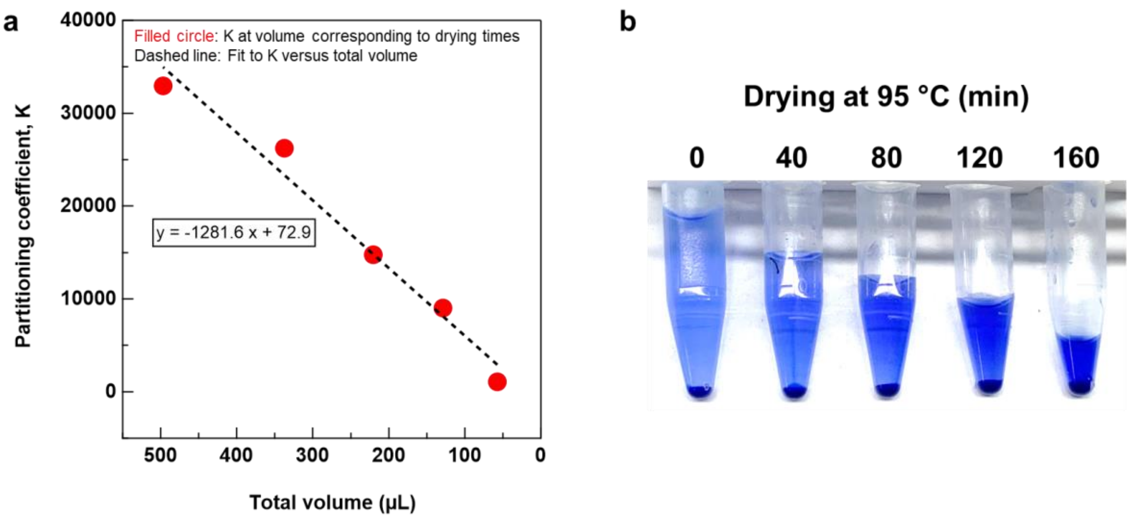

Supplementary Figure 15. a) Partitioning coefficient, K, of U15 within the 1:1 PDADMA:PAA coacervate calculated at each time point represented by the corresponding total volume. The U15 is added at increasing concentrations ranging between 0.02 and 0.2 µM. The volume of the coacervate phase stays constant (around 1.25 µL, see below for more details). b) Photographs of the dried samples with bromophenol blue dye.

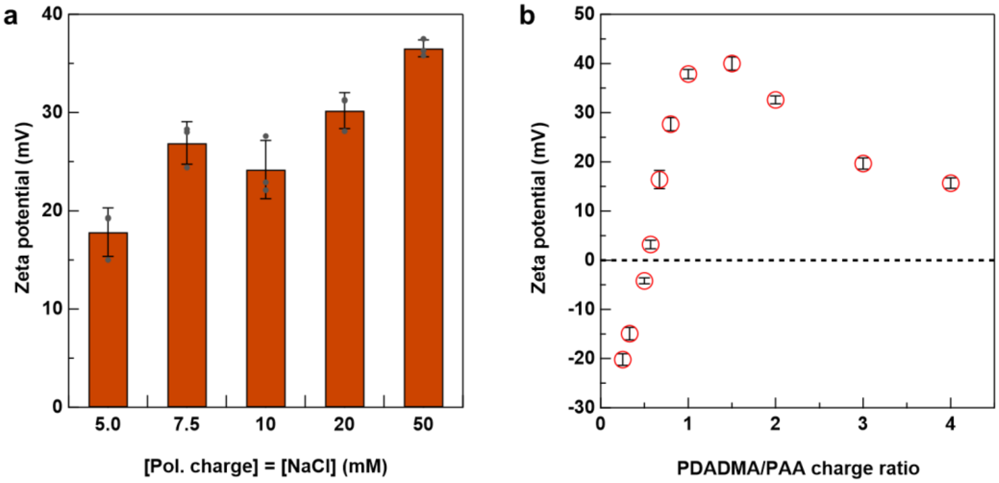

Supplementary Figure 16. a) Zeta potential of a series of "mimics" 1:1 PDADMA:PAA coacervate samples with 10-fold increase in the concentrations of all components, starting with 5 mM charge, 5 mM NaCl, 2.5 mM HEPES, and 0.43 mM $MgCl_2$. The increase in concentrations of the subsequent samples aimed at mimicking the range of concentrations obtained during water evaporation. Means and standard deviations are obtained from 3 trials each with at least 10 measurements per sample. Individual data points are shown as grey markers. b) Zeta potential of different PDADMA/PAA charge ratios. In these samples, NaCl, HEPES, and $MgCl_2$ were kept constant at 50, 25, and 4.3 mM. The polymer concentrations, with respect to the monomer, was 15 mM in the 1:1 sample. Increasing or decreasing the ratio was performed by increasing the concentration of one of the polymers 4-fold while keeping the other at 15 mM. Note: microscopy images have shown that higher PDADMA/PAA ratios (PDADMA:PAA of 3:1 and 4:1) do not produce significant amounts of coacervate droplets. Means and standard deviations are obtained from at least 10 measurements.

Supplementary Note 5: Zeta potential measurements

Zeta potential measurements were performed on large volumes of samples prepared at the same concentrations reached during each drying step, as it was impractical to perform them on the dried samples. The results showed an increase in the coacervate charge from +18 mV to +37 mV, with values similar to what was observed before with a similar polymer pair[1] (Supplementary Figure 16a). A decrease in charge interactions as the salt concentration increases could be causing this change in zeta potential, in addition to perturbations contributed by the buffer and Mg^{2+} molecules.[21] This measurement discounted the argument that a more negative coacervate could be repelling the nucleic acid and causing K to decrease. Zeta potential measurements on a series of different charge ratios PDADMA/PAA coacervates showed that the system became neutral around 1:2 PDADMA:PAA (Supplementary Figure 16b). Such a change in behavior has been observed before with coacervate systems that contain various charged components.[22] Zeta potential at various ratios, such as 1:1 and 1:4 PDADMA:PAA, agree with previously reported measurements with the same system.[1, 2]

Supplementary Table 2. Fitting parameters used in the FRAP experiments performed during 1 cycle (whole droplet fits). Std = standard deviation.

t = 0

Trial	ROI (μm)	Bckgd (μm)	τ (s)	std τ (s)	$\tau^{1/2}$ (s)	std $\tau^{1/2}$ (s)	*A*	Std *A*	*C*	std *C*	χ^2	*D* (μm^2 s^{-1})
1	5.85	6.00	504.8	0.9	349.9	0.6	1.00	0.000	0.00	0.000	0.00039	0.005
2	5.40	5.40	480.9	0.9	333.3	0.6	1.00	0.000	0.00	0.000	0.00042	0.005
3	5.50	5.00	460.0	0.8	318.9	0.6	1.00	0.000	0.00	0.000	0.00037	0.005
4	5.70	5.00	592.4	3.4	410.6	2.4	1.00	0.000	0.02	0.002	0.00023	0.004
5	5.00	4.00	576.6	4.0	399.7	2.8	0.98	0.003	0.00	0.003	0.00062	0.003
6	5.50	6.25	570.2	3.7	395.3	2.6	1.00	0.000	0.03	0.000	0.00288	0.004
7	5.75	5.25	872.4	3.6	604.7	2.5	1.08	0.002	0.00	0.000	0.00032	0.003
8	6.00	6.00	895.2	11.6	620.5	8.0	1.10	0.000	0.08	0.004	0.00160	0.003
9	5.90	5.20	824.7	5.0	571.7	3.5	1.05	0.002	0.01	0.002	0.00038	0.003
10	4.50	3.60	511.6	2.9	354.6	2.0	1.15	0.000	0.10	0.000	0.00276	0.003
			**Avg $\tau^{1/2}$ (s)**	**435.9**	**Std $\tau^{1/2}$ (s)**	**116.8**			**Avg *D***	**0.004**	**Std *D***	**0.0009**

t = 40 min

Trial	ROI (μm)	Bckgd (μm)	τ (s)	std τ (s)	$\tau^{1/2}$ (s)	std $\tau^{1/2}$ (s)	*A*	Std *A*	*C*	std *C*	χ^2	*D* (μm^2 s^{-1})
1	5.55	5.50	333.8	2.0	231.4	1.4	0.90	0.002	0.00	0.001	0.00010	0.007
2	5.20	5.50	253.2	1.4	175.5	1.0	1.05	0.002	0.00	0.002	0.00020	0.008
3	5.70	5.40	398.7	1.2	276.4	0.8	1.00	0.000	0.00	0.000	0.00101	0.006
4	5.50	5.50	413.0	1.1	286.2	0.8	1.11	0.034	0.01	0.001	0.00013	0.006
5	6.00	5.40	388.0	3.8	268.9	2.6	1.00	0.000	0.04	0.003	0.00092	0.007
6	5.50	5.80	443.0	4.1	307.1	2.8	1.00	0.000	0.07	0.003	0.00066	0.005
7	5.25	4.80	508.7	2.7	352.6	1.9	1.10	0.000	0.05	0.001	0.00065	0.004
8	6.10	5.50	516.3	2.0	357.9	1.4	1.06	0.002	0.01	0.002	0.00031	0.006
9	5.60	5.60	636.3	2.7	441.0	1.9	1.01	0.001	0.02	0.002	0.00017	0.004
10	5.75	4.75	467.6	1.8	324.1	1.3	0.97	0.002	0.00	0.002	0.00018	0.006
			**Avg $\tau^{1/2}$ (s)**	**302.1**	**Std $\tau^{1/2}$ (s)**	**73.4**			**Avg *D***	**0.006**	**Std *D***	**0.0014**

t = 80 min

Trial	ROI (μm)	Bckgd (μm)	τ (s)	std τ (s)	$\tau^{1/2}$ (s)	std $\tau^{1/2}$ (s)	*A*	Std *A*	*C*	std *C*	χ^2	*D* (μm^2 s^{-1})
1	6.00	6.00	222.3	0.9	154.1	0.7	0.88	0.001	0.02	0.001	0.00010	0.013
2	5.60	6.00	219.9	1.0	152.4	0.7	0.95	0.001	0.02	0.001	0.00014	0.011
3	5.25	5.00	245.8	1.8	170.4	1.2	1.00	0.000	0.03	0.002	0.00038	0.009
4	5.50	6.00	237.3	1.1	164.5	0.8	1.00	0.000	0.03	0.001	0.00016	0.010
5	5.90	4.25	198.6	1.0	137.7	0.7	0.86	0.002	0.02	0.002	0.00029	0.014
6	6.15	6.00	196.8	0.8	136.4	0.5	0.96	0.002	0.02	0.002	0.00023	0.015
7	6.25	6.60	359.4	0.9	249.1	0.6	1.14	0.001	0.00	0.000	0.00029	0.009
8	7.00	7.00	238.4	0.7	165.2	0.5	0.91	0.001	0.00	0.002	0.00015	0.016
9	6.90	5.50	398.2	1.0	276.0	0.7	1.05	0.001	0.00	0.000	0.00025	0.009
10	6.75	5.25	289.5	1.0	200.7	0.7	0.91	0.002	0.01	0.002	0.00022	0.012
			**Avg $\tau^{1/2}$ (s)**	**180.6**	**Std $\tau^{1/2}$ (s)**	**47.2**			**Avg *D***	**0.012**	**Std *D***	**0.0027**

Supplementary Table 2 (continued)

t = 120 min

Trial	ROI (μm)	Bckgd (μm)	τ (s)	std τ (s)	$\tau^{1/2}$ (s)	std $\tau^{1/2}$ (s)	A	Std A	C	std C	χ^2	D (μm^2 s^{-1})
1	7.75	7.25	181.1	0.5	125.6	0.4	1.00	0.001	0.00	0.000	0.00011	0.026
2	7.75	7.00	184.6	0.6	127.9	0.4	0.99	0.001	0.01	0.001	0.00011	0.026
3	6.80	6.80	177.7	0.7	123.2	0.5	1.00	0.000	0.02	0.001	0.00014	0.021
4	6.80	6.80	179.1	1.7	124.1	1.2	1.00	0.000	0.09	0.002	0.00089	0.020
5	7.00	5.50	139.5	0.3	96.7	0.2	1.01	0.000	0.00	0.000	0.00023	0.028
6	6.50	6.50	99.0	0.2	68.6	0.2	0.96	0.000	0.00	0.000	0.00020	0.034
7	7.00	6.00	121.1	0.3	84.0	0.2	0.92	0.000	0.00	0.000	0.00017	0.032
8	7.70	7.00	142.0	0.5	98.4	0.4	0.98	0.002	0.03	0.002	0.00029	0.033
9	6.90	6.50	98.7	0.2	68.4	0.2	1.09	0.000	0.00	0.000	0.00026	0.038
10	7.00	6.75	88.8	0.2	61.5	0.1	0.92	0.000	0.00	0.000	0.00015	0.044
			**Avg $\tau^{1/2}$ (s)**	**97.9**	**Std $\tau^{1/2}$ (s)**	**26.3**			**Avg D**	**0.030**	**Std D**	**0.0075**

t = 160 min

Trial	ROI (μm)	Bckgd (μm)	τ (s)	std τ (s)	$\tau^{1/2}$ (s)	std $\tau^{1/2}$ (s)	A	Std A	C	std C	χ^2	D (μm^2 s^{-1})
1	7.10	6.75	21.8	0.1	15.1	0.1	1.00	0.001	0.00	0.000	0.00054	0.183
2	7.20	7.40	23.7	0.1	16.5	0.1	0.98	0.001	0.00	0.000	0.00042	0.173
3	7.60	8.20	41.9	0.3	29.0	0.2	1.00	0.000	0.11	0.001	0.00084	0.109
4	8.70	8.70	36.9	0.1	25.6	0.1	1.00	0.000	0.00	0.001	0.00015	0.163
5	7.75	7.90	29.4	0.1	20.4	0.1	1.05	0.000	0.00	0.000	0.00034	0.162
6	7.00	8.00	26.3	0.1	18.2	0.1	1.10	0.001	0.00	0.000	0.00066	0.148
7	8.75	9.50	29.7	0.1	20.6	0.1	1.03	0.000	0.00	0.000	0.00034	0.204
8	7.50	7.25	19.8	0.1	13.7	0.1	0.99	0.000	0.00	0.000	0.00040	0.225
9	8.25	8.25	31.2	0.1	21.6	0.1	1.03	0.000	0.00	0.000	0.00027	0.173
10	8.00	8.25	23.2	0.1	16.1	0.0	0.97	0.000	0.00	0.000	0.00002	0.219
			**Avg $\tau^{1/2}$ (s)**	**19.7**	**Std $\tau^{1/2}$ (s)**	**4.8**			**Avg D**	**0.176**	**Std D**	**0.0345**

Rehydrated

Trial	ROI (μm)	Bckgd (μm)	τ (s)	std τ (s)	$\tau^{1/2}$ (s)	std $\tau^{1/2}$ (s)	A	Std A	C	std C	χ^2	D (μm^2 s^{-1})
1	5.50	5.40	334.2	0.9	231.6	0.6	1.00	0.000	0.00	0.000	0.00082	0.007
2	5.00	5.50	261.6	2.9	181.3	2.0	1.00	0.000	0.03	0.003	0.00087	0.008
3	5.40	4.50	406.5	2.1	281.8	1.5	1.00	0.000	0.03	0.002	0.00029	0.006
4	5.30	4.50	306.2	1.8	212.2	1.2	0.88	0.002	0.02	0.001	0.00018	0.007
5	5.20	4.75	729.4	1.0	505.6	0.7	1.02	0.003	0.00	0.000	0.00018	0.003
6	4.75	4.50	594.7	3.3	412.2	2.3	1.04	0.003	0.00	0.000	0.00025	0.003
7	4.75	4.50	892.4	5.3	618.5	3.7	1.08	0.002	0.02	0.002	0.00022	0.002
8	5.30	5.00	707.9	4.0	490.7	2.8	0.94	0.002	0.02	0.002	0.00023	0.003
9	5.90	5.30	896.5	1.9	621.4	1.3	1.00	0.000	0.00	0.000	0.00054	0.003
10	4.75	4.00	620.0	4.0	429.8	2.8	1.00	0.000	0.04	0.002	0.00041	0.003
			**Avg $\tau^{1/2}$ (s)**	**398.5**	**Std $\tau^{1/2}$ (s)**	**164.2**			**Avg D**	**0.004**	**Std D**	**0.0022**

Supplementary Table 3. Fitting parameters used in the FRAP experiments performed during 1 cycle (partial droplet fits).

t = 0

Trial	ROI (μm)	Bckgd (μm)	τ (s)	std τ (s)	$\tau^{1/2}$ (s)	std $\tau^{1/2}$ (s)	*A*	Std *A*	*C*	std *C*	χ^2	*D* (μm^2 s^{-1})
1	1.0	1.0	5.32	0.64	3.68	0.45	0.99	0.123	0.00	0.13	0.014	0.015
2	1.0	1.0	6.25	0.28	4.33	0.20	1.00	0.008	0.00	0.00	0.011	0.013
3	1.0	1.0	4.96	0.70	3.44	0.49	1.00	0.151	0.00	0.15	0.017	0.016
4	1.0	1.0	2.82	0.33	1.96	0.23	1.00	0.120	0.03	0.12	0.014	0.028
5	1.0	1.0	3.07	0.34	2.13	0.24	1.00	0.107	0.00	0.11	0.014	0.026
6	1.0	1.0	3.00	0.29	2.08	0.20	0.97	0.092	0.00	0.09	0.010	0.026
7	1.0	1.0	3.41	0.17	2.37	0.12	0.92	0.008	0.10	0.00	0.012	0.023
8	1.0	1.0	2.55	0.37	1.77	0.25	1.00	0.155	0.05	0.16	0.017	0.031
9	1.0	1.0	3.15	0.36	2.19	0.25	0.99	0.107	0.00	0.11	0.015	0.025
			**Avg $\tau^{1/2}$ (s)**	**2.66**	**Std $\tau^{1/2}$ (s)**	**0.862**			**Avg *D***	**0.023**	**Std *D***	**0.0065**

t = 40 min

Trial	ROI (μm)	Bckgd (μm)	τ (s)	std τ (s)	$\tau^{1/2}$ (s)	std $\tau^{1/2}$ (s)	*A*	Std *A*	*C*	std *C*	χ^2	*D* (μm^2 s^{-1})
1	1.0	1.0	3.04	0.44	2.11	0.30	0.98	0.205	0.00	0.21	0.006	0.026
2	1.0	1.0	3.11	0.50	2.15	0.35	1.00	0.230	0.02	0.23	0.008	0.026
3	1.0	1.0	2.43	0.55	1.69	0.38	0.98	0.371	0.00	0.37	0.008	0.033
4	1.0	1.0	2.55	0.22	1.77	0.16	1.00	0.095	0.01	0.10	0.006	0.031
5	1.0	1.0	2.56	0.22	1.78	0.15	1.00	0.091	0.00	0.09	0.006	0.031
6	1.0	1.0	2.31	0.20	1.60	0.14	1.00	0.100	0.02	0.10	0.005	0.034
7	1.0	1.0	2.65	0.22	1.83	0.15	1.00	0.086	0.00	0.09	0.006	0.030
8	1.0	1.0	2.54	0.20	1.76	0.14	0.98	0.082	0.00	0.08	0.005	0.031
9	1.0	1.0	2.84	0.21	1.97	0.15	1.00	0.075	0.00	0.08	0.005	0.028
			**Avg $\tau^{1/2}$ (s)**	**1.85**	**Std $\tau^{1/2}$ (s)**	**0.177**			**Avg *D***	**0.030**	**Std *D***	**0.0029**

t = 80 min

Trial	ROI (μm)	Bckgd (μm)	τ (s)	std τ (s)	$\tau^{1/2}$ (s)	std $\tau^{1/2}$ (s)	*A*	Std *A*	*C*	std *C*	χ^2	*D* (μm^2 s^{-1})
1	1.0	1.0	2.99	0.38	2.07	0.26	1.00	0.186	0.02	0.19	0.005	0.027
2	1.0	1.0	2.66	0.32	1.84	0.22	1.00	0.165	0.00	0.17	0.006	0.030
3	1.0	1.0	2.60	0.29	1.80	0.20	0.97	0.151	0.00	0.15	0.005	0.030
4	1.0	1.0	2.02	0.21	1.40	0.15	1.00	0.130	0.04	0.13	0.006	0.039
5	1.0	1.0	1.98	0.20	1.37	0.14	0.97	0.121	0.00	0.12	0.005	0.040
6	1.0	1.0	2.11	0.20	1.46	0.14	1.00	0.114	0.02	0.11	0.005	0.038
7	1.0	1.0	2.15	0.21	1.49	0.14	0.98	0.115	0.00	0.12	0.005	0.037
8	1.0	1.0	2.24	0.19	1.55	0.13	0.96	0.098	0.00	0.10	0.004	0.035
9	1.0	1.0	2.14	0.22	1.48	0.15	1.00	0.125	0.00	0.13	0.006	0.037
			**Avg $\tau^{1/2}$ (s)**	**1.61**	**Std $\tau^{1/2}$ (s)**	**0.226**			**Avg *D***	**0.035**	**Std *D***	**0.0047**

Supplementary Table 3 (continued)

t = 120 min

Trial	ROI (μm)	Bckgd (μm)	τ (s)	std τ (s)	$\tau^{1/2}$ (s)	std $\tau^{1/2}$ (s)	*A*	Std *A*	*C*	std *C*	χ^2	*D* (μm^2 s^{-1})
1	1.0	1.0	3.08	0.19	2.13	0.13	1.00	0.006	0.00	0.00	0.007	0.026
2	1.0	1.0	2.12	0.29	1.47	0.20	0.99	0.237	0.00	0.24	0.005	0.037
3	1.0	1.0	2.60	0.31	1.80	0.22	0.99	0.169	0.00	0.17	0.006	0.031
4	1.0	1.0	1.59	0.23	1.10	0.16	1.00	0.208	0.00	0.21	0.006	0.050
5	1.0	1.0	1.91	0.20	1.32	0.14	1.00	0.132	0.01	0.13	0.005	0.042
6	1.0	1.0	1.78	0.19	1.24	0.13	0.98	0.143	0.00	0.14	0.004	0.045
7	1.0	1.0	1.80	0.22	1.25	0.16	1.00	0.168	0.02	0.17	0.006	0.044
8	1.0	1.0	1.88	0.25	1.31	0.17	0.98	0.171	0.00	0.17	0.007	0.042
9	1.0	1.0	1.96	0.24	1.36	0.16	1.00	0.155	0.05	0.16	0.007	0.041
			**Avg $\tau^{1/2}$ (s)**	**1.44**	**Std $\tau^{1/2}$ (s)**	**0.306**			**Avg *D***	**0.040**	**Std *D***	**0.0074**

t = 160 min

Trial	ROI (μm)	Bckgd (μm)	τ (s)	std τ (s)	$\tau^{1/2}$ (s)	std $\tau^{1/2}$ (s)	*A*	Std *A*	*C*	std *C*	χ^2	*D* (μm^2 s^{-1})
1	1.0	1.0	2.13	0.27	1.48	0.19	1.00	0.214	0.00	0.22	0.004	0.037
2	1.0	1.0	1.74	0.29	1.21	0.20	1.00	0.359	0.00	0.36	0.005	0.046
3	1.0	1.0	2.53	0.35	1.76	0.25	1.00	0.270	0.01	0.27	0.005	0.031
4	1.0	1.0	1.37	0.24	0.95	0.16	0.98	0.273	0.00	0.27	0.006	0.058
5	1.0	1.0	1.49	0.25	1.03	0.17	0.99	0.251	0.00	0.25	0.007	0.053
6	1.0	1.0	1.43	0.23	0.99	0.16	0.98	0.242	0.00	0.24	0.005	0.055
7	1.0	1.0	1.61	0.23	1.12	0.16	1.00	0.209	0.04	0.21	0.006	0.049
8	1.0	1.0	1.58	0.23	1.09	0.16	1.00	0.215	0.02	0.22	0.006	0.050
9	1.0	1.0	1.70	0.17	1.18	0.12	0.96	0.110	0.00	0.11	0.005	0.047
			**Avg $\tau^{1/2}$ (s)**	**1.20**	**Std $\tau^{1/2}$ (s)**	**0.245**			**Avg *D***	**0.047**	**Std *D***	**0.0086**

Rehydrated

Trial	ROI (μm)	Bckgd (μm)	τ (s)	std τ (s)	$\tau^{1/2}$ (s)	std $\tau^{1/2}$ (s)	*A*	Std *A*	*C*	std *C*	χ^2	*D* (μm^2 s^{-1})
1	1.0	1.0	3.54	0.31	2.45	0.21	1.00	0.093	0.02	0.09	0.005	0.022
2	1.0	1.0	3.27	0.32	2.27	0.22	1.00	0.110	0.00	0.11	0.006	0.024
3	1.0	1.0	2.88	0.30	2.00	0.21	0.99	0.133	0.00	0.13	0.005	0.028
4	1.0	1.0	2.69	0.17	1.87	0.12	1.00	0.067	0.00	0.07	0.004	0.029
5	1.0	1.0	2.88	0.14	1.99	0.10	1.00	0.049	0.00	0.05	0.002	0.028
6	1.0	1.0	2.80	0.15	1.94	0.10	0.99	0.054	0.00	0.05	0.003	0.028
7	1.0	1.0	2.15	0.17	1.49	0.12	1.00	0.076	0.00	0.08	0.005	0.037
8	1.0	1.0	2.99	0.17	2.07	0.12	0.99	0.057	0.00	0.06	0.003	0.027
9	1.0	1.0	2.52	0.20	1.75	0.14	0.99	0.086	0.00	0.09	0.005	0.031
			**Avg $\tau^{1/2}$ (s)**	**1.98**	**Std $\tau^{1/2}$ (s)**	**0.263**			**Avg *D***	**0.028**	**Std *D***	**0.0042**

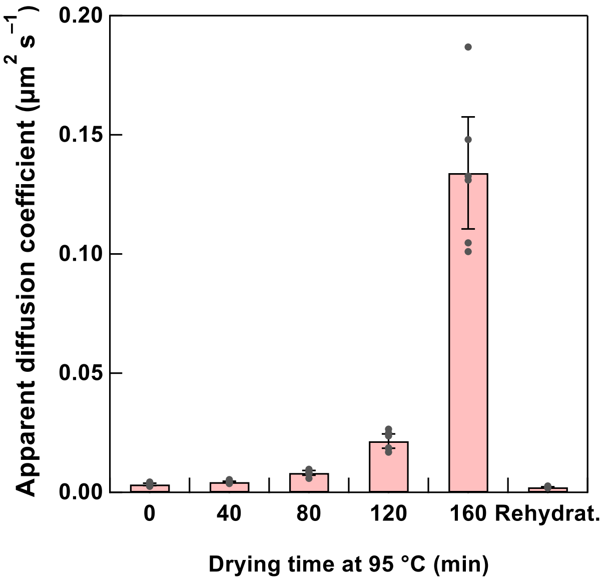

Supplementary Figure 17. FRAP performed with U15 partitioned within 1:1 PDADMA:PAA coacervates with the RNA added at a constant concentration of 0.12 µM. The bleached area is equal to a whole coacervate droplet. Means and standard deviations are obtained from 3 samples with 2 bleached droplets per sample. Individual data points are shown as grey markers.

Supplementary Note 6: Troubleshooting the whole-droplet exchange experiment with equal [U15]

We wanted to examine whether the increase in diffusion stemmed from adding U15 in increasing concentrations from 0.02 µM to 0.2 µM in Figure 3e (similar to the experiment in Figure 2c). This was done to ensure a good level of fluorescence for FRAP. This experiment was thus repeated with a constant concentration of U15 equal to 0.12 µM – higher than the first 4 samples in the first experiment. The result (Supplementary Figure 17 and Supplementary Table 4) was not significantly different from the values obtained in Figure 3e, further supporting the role of salt concentration, and the loss of water in the solution, in delivering this trend.

Supplementary Table 4. Fitting parameters for whole droplet FRAP with constant initial [U15].

t = 0

Trial	ROI (μm)	Bckgd (μm)	τ (s)	std τ (s)	$\tau^{1/2}$ (s)	std $\tau^{1/2}$ (s)	A	Std A	C	std C	χ^2	D (μm^2 s^{-1})
1	5.50	4.20	690.3	1.9	478.5	1.3	1.08	0.001	0.02	0.000	0.00020	0.003
2	4.70	4.25	397.4	1.4	275.5	1.0	0.85	0.002	0.01	0.002	0.00013	0.004
3	4.65	4.00	690.5	2.7	478.6	1.9	0.94	0.001	0.00	0.000	0.00029	0.002
4	4.60	3.75	443.9	0.8	307.7	0.6	0.95	0.000	0.00	0.000	0.00008	0.004
5	5.30	5.20	862.8	2.6	598.0	1.8	0.99	0.001	0.01	0.001	0.00007	0.003
6	4.60	4.30	523.4	1.6	362.8	1.1	0.92	0.001	0.04	0.001	0.00010	0.003
			**Avg $\tau^{1/2}$ (s)**	**416.9**	**Std $\tau^{1/2}$ (s)**	**122.7**			**Avg D**	**0.003**	**Std D**	**0.0007**

t = 40 min

Trial	ROI (μm)	Bckgd (μm)	τ (s)	std τ (s)	$\tau^{1/2}$ (s)	std $\tau^{1/2}$ (s)	A	Std A	C	std C	χ^2	D (μm^2 s^{-1})
1	5.15	5.15	395.5	0.9	274.2	0.6	1.04	0.001	0.00	0.001	0.00008	0.005
2	5.05	4.40	460.1	1.2	318.9	0.8	1.00	0.001	0.01	0.001	0.00009	0.004
3	4.90	4.60	517.7	1.4	358.9	1.0	0.98	0.000	0.00	0.000	0.00062	0.004
4	5.00	4.60	488.0	1.1	338.3	0.8	0.96	0.001	0.00	0.001	0.00006	0.004
5	6.10	5.80	767.4	2.0	531.9	1.4	0.98	0.001	0.03	0.001	0.45957	0.004
6	5.75	5.80	560.7	0.0	388.6	0.0	0.97	0.001	0.04	0.001	0.00011	0.005
			**Avg $\tau^{1/2}$ (s)**	**368.5**	**Std $\tau^{1/2}$ (s)**	**88.9**			**Avg D**	**0.004**	**Std D**	**0.0006**

t = 80 min

Trial	ROI (μm)	Bckgd (μm)	τ (s)	std τ (s)	$\tau^{1/2}$ (s)	std $\tau^{1/2}$ (s)	A	Std A	C	std C	χ^2	D (μm^2 s^{-1})
1	8.20	7.70	577.9	1.1	400.6	0.8	1.10	0.000	0.00	0.000	0.00074	0.009
2	8.50	8.20	592.1	1.2	410.4	0.8	1.00	0.001	0.01	0.001	0.00004	0.010
3	5.70	5.20	328.5	0.6	227.7	0.4	0.99	0.001	0.00	0.000	0.00014	0.008
4	5.25	4.80	271.7	0.8	188.4	0.5	1.02	0.001	0.01	0.002	0.00017	0.008
5	5.90	6.25	469.5	1.1	325.4	0.8	1.12	0.001	0.05	0.001	0.00010	0.006
6	6.40	5.25	390.6	1.5	270.8	1.0	0.95	0.000	0.05	0.001	0.00025	0.008
			**Avg $\tau^{1/2}$ (s)**	**303.9**	**Std $\tau^{1/2}$ (s)**	**91.0**			**Avg D**	**0.008**	**Std D**	**0.0013**

t = 120 min

Trial	ROI (μm)	Bckgd (μm)	τ (s)	std τ (s)	$\tau^{1/2}$ (s)	std $\tau^{1/2}$ (s)	A	Std A	C	std C	χ^2	D (μm^2 s^{-1})
1	7.20	7.00	244.7	0.7	169.6	0.5	1.14	0.001	0.00	0.000	0.00038	0.017
2	7.15	6.75	221.5	0.6	153.5	0.4	1.06	0.001	0.03	0.001	0.00015	0.018
3	7.00	5.80	208.1	0.6	144.2	0.4	0.98	0.001	0.00	0.001	0.00016	0.019
4	7.50	6.80	179.3	0.3	124.3	0.2	0.97	0.000	0.00	0.000	0.00015	0.025
5	7.30	7.70	159.7	0.4	110.7	0.2	0.87	0.001	0.01	0.001	0.00008	0.026
6	7.90	8.60	209.9	0.4	145.5	0.3	0.99	0.001	0.04	0.001	0.00008	0.024
			**Avg $\tau^{1/2}$ (s)**	**141.3**	**Std $\tau^{1/2}$ (s)**	**21.0**			**Avg D**	**0.021**	**Std D**	**0.0040**

t = 160 min

Trial	ROI (μm)	Bckgd (μm)	τ (s)	std τ (s)	$\tau^{1/2}$ (s)	std $\tau^{1/2}$ (s)	A	Std A	C	std C	χ^2	D (μm^2 s^{-1})
1	8.50	8.75	30.7	0.1	21.3	0.1	0.98	0.001	0.00	0.000	0.00044	0.187
2	7.60	7.00	34.5	0.1	23.9	0.1	1.00	0.001	0.00	0.000	0.00036	0.133
3	8.50	8.00	43.8	0.2	30.3	0.1	1.02	0.000	0.00	0.000	0.00079	0.131
4	8.75	8.70	41.0	0.1	28.4	0.1	0.98	0.000	0.00	0.000	0.00022	0.148
5	8.40	7.50	53.5	0.1	37.1	0.1	1.00	0.000	0.00	0.000	0.00017	0.105
6	8.40	9.00	55.4	0.1	38.4	0.1	0.96	0.000	0.00	0.000	0.00016	0.101
			**Avg $\tau^{1/2}$ (s)**	**29.9**	**Std $\tau^{1/2}$ (s)**	**6.9**			**Avg D**	**0.134**	**Std D**	**0.0314**

Supplementary Table 4 (continued)

Rehydrated

**Trial**	**ROI (μm)**	**Bckgd (μm)**	**τ (s)**	**std τ (s)**	**$\tau^{1/2}$ (s)**	**std $\tau^{1/2}$ (s)**	***A***	**Std *A***	***C***	**std *C***	**χ^2**	***D* (μm^2 s^{-1})**
1	4.00	3.10	536.6	1.7	371.9	1.2	0.84	0.001	0.00	0.000	0.00016	0.002
2	4.25	4.40	705.3	8.4	488.9	5.8	1.00	0.000	0.05	0.003	0.00120	0.002
3	4.75	4.75	936.8	7.7	649.3	5.3	0.99	0.000	0.04	0.002	0.00056	0.002
4	5.40	4.30	1095.8	3.7	759.5	2.6	0.01	0.001	0.01	0.001	0.00016	0.002
5	4.90	4.80	849.8	4.2	589.1	2.9	1.00	0.000	0.00	0.001	0.00020	0.002
6	5.00	5.10	759.4	5.2	526.4	3.6	1.02	0.000	0.07	0.002	0.00040	0.003
			**Avg $\tau^{1/2}$ (s)**	**564.2**	**Std $\tau^{1/2}$ (s)**	**134.2**			**Avg *D***	**0.002**	**Std *D***	**0.0003**

Supplementary Discussion 1: Discussion of the movements on the phase diagram

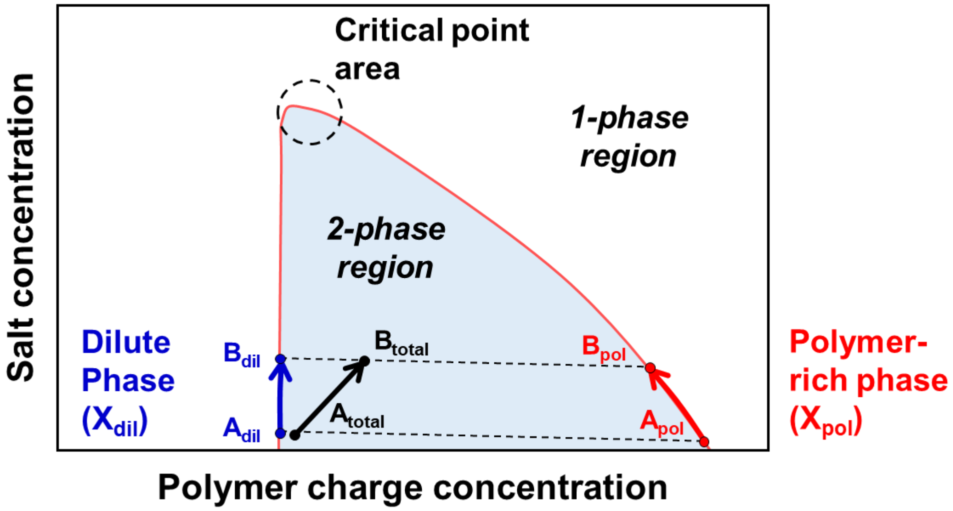

Supplementary Figure 18. The movements on the phase diagram are shown on a generic approximate binodal curve and tie-lines (dashed lines which project to dilute phase and polymer-rich phase compositions on the binodal) in addition to the diagonal movement of the overall system composition inside the two-phase region. The location of the total composition on the tie-lines inside the two-phase region is related to the volume fractions of the phases. The critical point area (dashed circle), where the two phases eventually become the same, is also pointed out. A projection of the diagonal movement on the binodal curve provides insights into the expected compositions of each phase.

Supplementary Figure 18 shows the projection of the diagonal movement of the coacervate samples onto an expected binodal curve which reveals insights into the predicted composition of each phase. While samples increase tenfold in overall concentration ($A_{total} \rightarrow B_{total}$), the polymer concentration in the coacervate phase is in fact expected to decrease ($A_{pol} \rightarrow B_{pol}$ shows a leftward move on the x-axis), accompanied by a minimal increase in the dilute phase ($A_{dil} \rightarrow B_{dil}$). In our system, these changes do not appear to be on an equal magnitude as the overall sample concentration ($A_{total} \rightarrow B_{total}$ change seems to be bigger than $A_{pol} \rightarrow B_{pol}$ change). Simultaneously, revisions to the Voorn-Overbeek theory on phase separation[23] predict a tilt in the tie-lines (downward slope) which means slightly more salt could be present in the dilute phase than in the polymer-rich phase. As we demonstrate in Figure 5, the volume that the coacervate phase occupies in the total sample volume increases. These parallel changes in

volume and composition of the polymer-rich phase lead to an increase in its hydration, which explains the observed partitioning and diffusion outcomes. As we mention in the main text, this is usually accompanied by self-suppression, which is shown by the decrease of the polymer concentration in the coacervate phase. We note here that these changes in the polymer-rich phase are determined by the boundary approximately defined by sample turbidity, but could be affected by the presence of other components in the system ($MgCl_2$ and HEPES) which could also impact the partitioning. As the phases become more similar, it is reasonable to expect reduced partitioning of not only the polymers themselves, but also other solutes such as the added U15 RNA. Here, the decrease of RNA partitioning is manifested by a constant [RNA] inside the coacervate phase and its increase in the dilute phase. The counterintuitive decrease in partitioning is consistent with the shorter tie-line length at higher ionic strength. However, [RNA] in the polymer-rich phase does not decrease (Figure 2), as is anticipated for the total polymer content based on the binodal curve. This could be affected by the higher volume fraction of the coacervate phase (the diagonal movement encourages formation of polymer-rich phase), as shown below, but also by an increase in the overall [U15] in the solution mixture. We note that the shape of the binodal is not precisely defined in our turbidity experiments (Figure 1b), and hence the anticipated reduction in total polymer concentration in the coacervate phase could be quite small. Further, the RNA may influence the position of the binodal and/or behave differently from the PDADMA and PAA; for example, as a polyanion, it can be expected to compete with PAA for ion pairing interactions with the PDADMA.

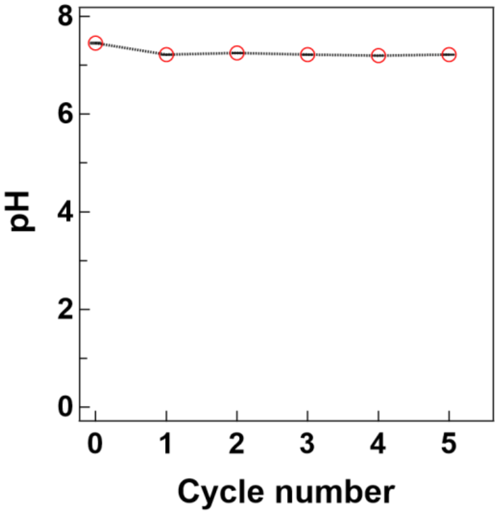

Supplementary Figure 19. pH of the turbid PDADMA/PAA coacervate (pH probe is inserted in the solution where the coacervate is suspended) at t = 0 and after the rehydration step of each wet-dry cycle. Means and standard deviations are obtained from at least 2 samples, with individual data points too close to distinguish.

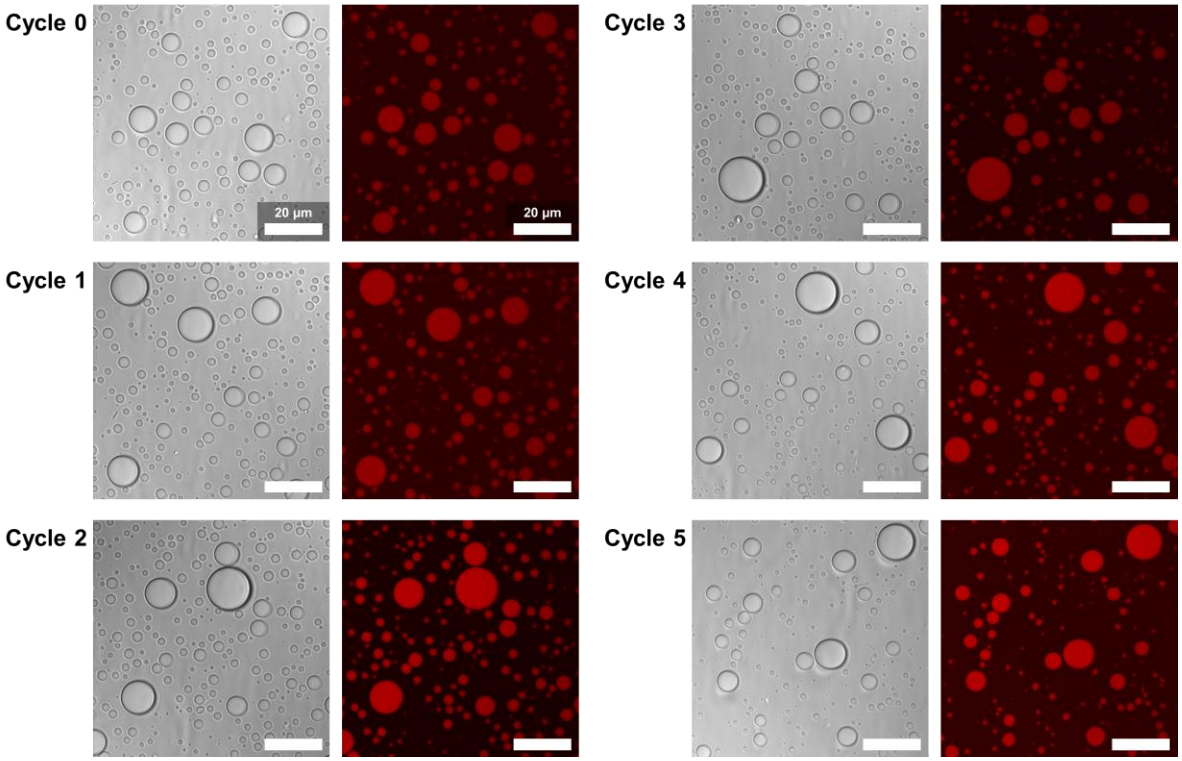

Supplementary Figure 20. Examples of DIC and fluorescent microscopy images of PDADMA/PAA coacervates with Alexa 647-U15 partitioned within them before cycles (Cycle 0) and after the rehydration step of each cycle. U15 is added at a final concentration of 0.04 μM. Left panels are DIC and right panels are false colored fluorescence.

Supplementary Note 7: Statistical analysis for Figures 4 and 5

Figure 4b:

Single-factor ANOVA test performed among different groups of cycles – difference is not significant between cycles 1-4. Cycles 0 and 5 are different, but less significantly for cycle 5:

- **Among all samples:** P = 1.12E-7 (P < 0.05, difference of [U15] values between cycles is significant). F = 11.68; degrees of freedom between cycles, $df1$ = 5; degrees of freedom within samples, $df2$ = 54.
- **Among cycles 0 – 4:** P = 0.00038 (P < 0.05, difference of [U15] values between these cycles is significant). F = 6.36; $df1$ = 4; $df2$ = 45.
- **Among cycles 1 – 5:** P = 0.0025 (P < 0.05, difference of [U15] values between these cycles is significant, but less than cycles 0 – 4). F = 4.82; $df1$ = 4; $df2$ = 45.
- **Among cycles 1 – 4:** P = 0.70 (P > 0.05, difference of [U15] values between these cycles is not significant). F = 0.48; $df1$ = 3; $df2$ = 36.

Figure 4c:

Single-factor ANOVA test:

- Among all samples: P = 0.14 (P > 0.05, difference of partitioning coefficients among cycles is not significant). F = 2.59; $df1$ = 5; $df2$ = 6.

Figure 5c:

Single-factor ANOVA test:

- Among all samples: P = 0.17 (P > 0.05, difference of [U15] values among samples that are dried and the studied mimics is not significant). F = 1.69; $df1$ = 8; $df2$ = 18.

Figure 5d:

Two-sample t-test, assuming equal variances:

- Among 5 mM samples: $P = 0.025$ ($P < 0.05$, the values are considered different).

  Nonetheless the scaled values (Supplementary Figure 21) are similar.

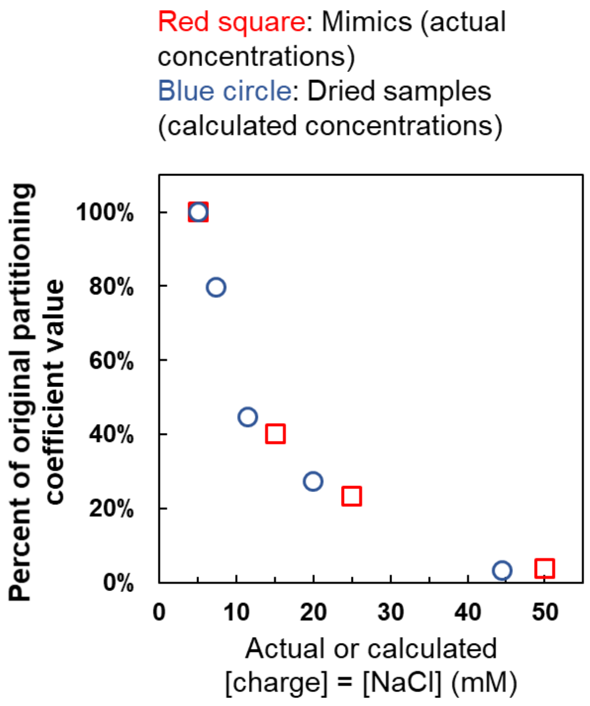

Supplementary Figure 21. Percent of original U15 partitioning coefficient values in dried and mimics PDADMA/PAA complex coacervate samples.

Supplementary Table 5. Fitting parameters used for the FRAP experiments of the repeating cycles samples (whole droplet FRAP fits).

Cycle 0

Trial	ROI (µm)	Bckgd (µm)	τ (s)	std τ (s)	$\tau^{1/2}$ (s)	std $\tau^{1/2}$ (s)	*A*	Std *A*	*C*	std *C*	χ^2	*D* (µm^2 s^{-1})
1	7.0	6.0	384.0	3.1	266.2	2.1	0.72	0.002	0.12	0.002	0.00030	0.010
2	6.9	6.4	572.7	7.8	397.0	5.4	0.89	0.004	0.23	0.004	0.00039	0.007
3	6.3	6.5	461.9	5.6	320.2	3.9	0.91	0.004	0.23	0.004	0.00044	0.007
4	6.7	6.2	515.8	7.3	357.5	5.0	0.70	0.003	0.24	0.003	0.00031	0.007
5	6.7	7.2	406.9	4.4	282.1	3.0	0.77	0.003	0.26	0.003	0.00029	0.009
6	6.8	6.0	433.8	5.9	300.7	4.1	0.76	0.004	0.29	0.004	0.00043	0.008
			**Avg τ (s)**	**320.6**	**Std τ (s)**	**49.1**			**Avg (*D*)**	**0.008**	**Std *D***	**0.001**

Cycle 1

Trial	ROI (µm)	Bckgd (µm)	τ (s)	std τ (s)	$\tau^{1/2}$ (s)	std $\tau^{1/2}$ (s)	*A*	Std *A*	*C*	std *C*	χ^2	*D* (µm^2 s^{-1})
1	6.6	7.0	584.5	4.6	405.1	3.2	0.88	0.002	0.08	0.002	0.00013	0.006
2	6.4	5.3	490.0	4.7	339.7	3.3	0.84	0.003	0.13	0.003	0.00023	0.007
3	6.9	7.2	612.3	5.4	424.4	3.8	0.88	0.002	0.14	0.002	0.00015	0.006
4	6.0	8.0	401.9	3.8	278.6	2.7	0.78	0.003	0.19	0.003	0.00024	0.007
5	6.8	5.6	503.1	5.1	348.7	3.6	0.78	0.003	0.16	0.003	0.00021	0.007
6	6.1	5.3	353.1	3.5	244.8	2.4	0.77	0.003	0.19	0.003	0.00028	0.008
			**Avg τ (s)**	**340.2**	**Std τ (s)**	**69.7**			**Avg (*D*)**	**0.007**	**Std *D***	**0.001**

Cycle 2

Trial	ROI (µm)	Bckgd (µm)	τ (s)	std τ (s)	$\tau^{1/2}$ (s)	std $\tau^{1/2}$ (s)	*A*	Std *A*	*C*	std *C*	χ^2	*D* (µm^2 s^{-1})
1	6.6	6.6	577.5	4.4	400.3	3.0	0.99	0.002	0.03	0.002	0.00015	0.006
2	6.7	6.6	459.3	3.0	318.3	2.1	0.93	0.002	0.02	0.002	0.00014	0.008
3	6.5	6.4	396.4	2.7	274.7	1.9	0.94	0.002	0.02	0.003	0.00018	0.008
4	6.7	6.4	630.4	6.0	436.9	4.1	0.94	0.003	0.05	0.002	0.00018	0.006
5	6.5	6.5	468.5	3.5	324.7	2.5	0.99	0.002	0.03	0.003	0.00020	0.007
6	6.6	8.6	554.4	5.4	384.3	3.7	1.00	0.003	0.05	0.003	0.00027	0.006
			**Avg τ (s)**	**356.5**	**Std τ (s)**	**60.5**			**Avg (*D*)**	**0.007**	**Std *D***	**0.001**

Cycle 3

Trial	ROI (µm)	Bckgd (µm)	τ (s)	std τ (s)	$\tau^{1/2}$ (s)	std $\tau^{1/2}$ (s)	*A*	Std *A*	*C*	std *C*	χ^2	*D* (µm^2 s^{-1})
1	6.2	6.1	508.0	4.0	352.2	2.7	0.93	0.002	0.03	0.002	0.00017	0.006
2	6.6	7.5	527.8	4.2	365.9	2.9	0.95	0.002	0.04	0.002	0.00017	0.007
3	7.4	7.8	538.7	3.9	373.4	2.7	0.97	0.002	0.04	0.002	0.00015	0.008
4	6.9	6.6	542.9	5.0	376.3	3.5	0.89	0.003	0.04	0.003	0.00020	0.007
5	6.0	7.3	384.4	3.7	266.4	2.6	0.91	0.003	0.07	0.004	0.00034	0.007
6	6.1	5.6	374.4	3.6	259.5	2.5	0.94	0.004	0.05	0.004	0.00037	0.008
			**Avg τ (s)**	**332.3**	**Std τ (s)**	**54.4**			**Avg (*D*)**	**0.007**	**Std *D***	**0.001**

Supplementary Table 5 (continued)

Cycle 4

Trial	ROI (μm)	Bckgd (μm)	τ (s)	std τ (s)	$\tau^{1/2}$ (s)	std $\tau^{1/2}$ (s)	A	Std A	C	std C	χ^2	D (μm^2 s^{-1})
1	5.9	6.5	502.6	5.1	348.4	3.5	0.80	0.003	0.16	0.003	0.00022	0.005
2	6.2	5.3	586.7	6.5	406.7	4.5	0.88	0.003	0.19	0.003	0.00027	0.005
3	6.5	7.4	576.6	6.1	399.6	4.2	0.80	0.003	0.19	0.002	0.00019	0.006
4	5.6	5.0	434.0	4.0	300.8	2.8	0.86	0.003	0.13	0.003	0.00025	0.006
5	6.0	5.7	505.8	4.9	350.6	3.4	0.91	0.003	0.13	0.003	0.00026	0.006
6	6.5	6.4	457.2	3.7	316.9	2.6	0.88	0.002	0.15	0.003	0.00019	0.007
			**Avg τ (s)**	**353.8**	**Std τ (s)**	**42.7**			**Avg (D)**	**0.006**	**Std D**	**0.001**

Cycle 5

Trial	ROI (μm)	Bckgd (μm)	τ (s)	std τ (s)	$\tau^{1/2}$ (s)	std $\tau^{1/2}$ (s)	A	Std A	C	std C	χ^2	D (μm^2 s^{-1})
1	6.2	5.5	453.4	3.8	314.3	2.7	0.9	0.002	0.12	0.003	0.00020	0.007
2	5.7	5.5	309.3	2.8	214.4	1.9	0.8	0.003	0.16	0.004	0.00029	0.008
3	6.2	6.8	350.4	2.7	242.9	1.9	0.8	0.003	0.16	0.003	0.00018	0.009
4	5.7	6.1	544.1	4.5	377.2	3.1	1.0	0.003	0.12	0.003	0.00019	0.005
5	7.1	6.2	643.6	5.2	446.1	3.6	0.9	0.002	0.11	0.002	0.00011	0.006
6	6.5	6.5	570.8	4.6	395.7	3.2	0.9	0.002	0.13	0.002	0.00014	0.006
			**Avg τ (s)**	**331.7**	**Std τ (s)**	**90.8**			**Avg (D)**	**0.007**	**Std D**	**0.002**

Supplementary Table 6. Fitting parameters used for the FRAP experiments of the repeating cycles samples (partial droplet fits).

Cycle 0

Trial	ROI (μm)	Bckgd (μm)	τ (s)	std τ (s)	$\tau^{1/2}$ (s)	std $\tau^{1/2}$ (s)	A	Std A	C	std C	χ^2	D (μm^2 s^{-1})
1	1.0	1.0	2.72	0.35	1.88	0.24	0.97	0.112	0.03	0.11	0.020	0.029
2	1.0	1.0	2.83	0.43	1.96	0.30	0.77	0.102	0.26	0.10	0.018	0.028
3	1.0	1.0	2.44	0.14	1.69	0.10	0.94	0.008	0.00	0.00	0.014	0.033
4	1.0	1.0	2.43	0.14	1.69	0.10	0.95	0.008	0.00	0.00	0.013	0.033
5	1.0	1.0	2.25	0.14	1.56	0.10	0.88	0.008	0.00	0.00	0.013	0.035
6	1.0	1.0	1.86	0.13	1.29	0.09	0.94	0.008	0.00	0.00	0.015	0.043
7	1.0	1.0	1.91	0.12	1.33	0.09	0.95	0.008	0.00	0.00	0.014	0.041
8	1.0	1.0	2.65	0.16	1.83	0.11	0.84	0.008	0.10	0.00	0.014	0.030
9	1.0	1.0	2.36	0.15	1.64	0.10	0.84	0.008	0.10	0.00	0.013	0.034
10	1.0	1.0	2.57	0.16	1.78	0.11	0.97	0.009	0.05	0.00	0.017	0.031
			**Avg τ (s)**	**1.67**	**Std τ (s)**	**0.222**			**Avg D**	**0.034**	**Std D**	**0.0049**

Cycle 1

Trial	ROI (μm)	Bckgd (μm)	τ (s)	std τ (s)	$\tau^{1/2}$ (s)	std $\tau^{1/2}$ (s)	A	Std A	C	std C	χ^2	D (μm^2 s^{-1})
1	1.0	1.0	1.82	0.25	1.26	0.18	1.00	0.152	0.04	0.15	0.013	0.044
2	1.0	1.0	2.67	0.38	1.85	0.26	0.71	0.092	0.35	0.09	0.013	0.030
3	1.0	1.0	1.91	0.28	1.32	0.20	0.85	0.136	0.14	0.14	0.012	0.042
4	1.0	1.0	1.91	0.12	1.33	0.08	0.85	0.007	0.10	0.00	0.011	0.041
5	1.0	1.0	1.91	0.10	1.32	0.07	0.95	0.007	0.00	0.00	0.010	0.042
6	1.0	1.0	1.95	0.11	1.35	0.08	0.98	0.007	0.10	0.00	0.012	0.041
7	1.0	1.0	2.07	0.32	1.44	0.23	0.66	0.105	0.26	0.11	0.009	0.038
8	1.0	1.0	1.87	0.13	1.29	0.09	0.83	0.007	0.20	0.00	0.012	0.042
9	1.0	1.0	1.77	0.13	1.23	0.09	0.81	0.007	0.20	0.00	0.011	0.045
10	1.0	1.0	1.94	0.28	1.34	0.20	0.81	0.125	0.24	0.13	0.011	0.041
			**Avg τ (s)**	**1.37**	**Std τ (s)**	**0.176**			**Avg D**	**0.041**	**Std D**	**0.0042**

Cycle 2

Trial	ROI (μm)	Bckgd (μm)	τ (s)	std τ (s)	$\tau^{1/2}$ (s)	std $\tau^{1/2}$ (s)	A	Std A	C	std C	χ^2	D (μm^2 s^{-1})
1	1.0	1.0	2.04	0.12	1.42	0.08	0.95	0.007	0.10	0.00	0.013	0.039
2	1.0	1.0	1.90	0.11	1.32	0.07	1.00	0.007	0.00	0.00	0.012	0.042
3	1.0	1.0	1.76	0.10	1.22	0.07	1.00	0.007	0.00	0.00	0.012	0.045
4	1.0	1.0	1.85	0.11	1.28	0.07	1.00	0.007	0.00	0.00	0.012	0.043
5	1.0	1.0	2.09	0.28	1.45	0.20	0.86	0.119	0.18	0.12	0.012	0.038
6	1.0	1.0	1.90	0.11	1.32	0.08	0.96	0.007	0.00	0.00	0.012	0.042
7	1.0	1.0	1.64	0.11	1.14	0.08	1.00	0.008	0.00	0.00	0.015	0.048
8	1.0	1.0	2.01	0.12	1.39	0.09	0.96	0.008	0.00	0.00	0.014	0.040
9	1.0	1.0	2.30	0.32	1.59	0.22	0.83	0.113	0.17	0.12	0.014	0.035
10	1.0	1.0	1.92	0.28	1.33	0.19	1.00	0.154	0.03	0.16	0.016	0.041
			**Avg τ (s)**	**1.35**	**Std τ (s)**	**0.127**			**Avg D**	**0.041**	**Std D**	**0.0039**

Supplementary Table 6 (continued)

Cycle 3

Trial	ROI (µm)	Bckgd (µm)	τ (s)	std τ (s)	$\tau^{1/2}$ (s)	std $\tau^{1/2}$ (s)	*A*	Std *A*	*C*	std *C*	χ^2	*D* (µm^2 s^{-1})
1	1.0	1.0	2.10	0.12	1.45	0.09	0.88	0.007	0.10	0.00	0.011	0.038
2	1.0	1.0	1.82	0.10	1.26	0.07	0.91	0.006	0.00	0.00	0.009	0.044
3	1.0	1.0	2.21	0.11	1.53	0.08	0.98	0.007	0.00	0.00	0.011	0.036
4	1.0	1.0	1.75	0.09	1.21	0.06	0.91	0.006	0.00	0.00	0.008	0.045
5	1.0	1.0	1.82	0.10	1.26	0.07	0.97	0.006	0.00	0.00	0.010	0.044
6	1.0	1.0	2.02	0.13	1.40	0.09	0.98	0.008	0.00	0.00	0.015	0.039
7	1.0	1.0	1.78	0.13	1.23	0.09	0.93	0.008	0.10	0.00	0.016	0.045
8	1.0	1.0	1.82	0.28	1.26	0.19	0.95	0.160	0.05	0.16	0.014	0.044
9	1.0	1.0	1.75	0.33	1.21	0.23	1.00	0.207	0.12	0.21	0.022	0.045
10	1.0	1.0	1.94	0.34	1.34	0.23	0.92	0.168	0.09	0.17	0.019	0.041
			**Avg τ (s)**	**1.32**	**Std τ (s)**	**0.111**			**Avg *D***	**0.042**	**Std *D***	**0.0033**

Cycle 4

Trial	ROI (µm)	Bckgd (µm)	τ (s)	std τ (s)	$\tau^{1/2}$ (s)	std $\tau^{1/2}$ (s)	*A*	Std *A*	*C*	std *C*	χ^2	*D* (µm^2 s^{-1})
1	1.0	1.0	1.86	0.11	1.29	0.07	0.98	0.007	0.10	0.00	0.011	0.043
2	1.0	1.0	1.78	0.09	1.24	0.07	0.94	0.006	0.05	0.00	0.009	0.045
3	1.0	1.0	1.70	0.10	1.18	0.07	0.90	0.006	0.10	0.00	0.010	0.047
4	1.0	1.0	1.64	0.08	1.14	0.06	0.90	0.005	0.10	0.00	0.006	0.048
5	1.0	1.0	2.32	0.36	1.61	0.25	0.63	0.095	0.39	0.10	0.010	0.034
6	1.0	1.0	2.43	0.32	1.68	0.22	0.64	0.081	0.29	0.08	0.008	0.033
7	1.0	1.0	2.36	0.33	1.64	0.23	0.66	0.088	0.33	0.09	0.009	0.034
8	1.0	1.0	2.02	0.10	1.40	0.07	0.89	0.006	0.10	0.00	0.008	0.039
9	1.0	1.0	1.83	0.10	1.27	0.07	0.87	0.006	0.10	0.00	0.008	0.043
10	1.0	1.0	1.80	0.25	1.25	0.17	0.67	0.101	0.23	0.10	0.006	0.044
			**Avg τ (s)**	**1.37**	**Std τ (s)**	**0.201**			**Avg *D***	**0.041**	**Std *D***	**0.0056**

Cycle 5

Trial	ROI (µm)	Bckgd (µm)	τ (s)	std τ (s)	$\tau^{1/2}$ (s)	std $\tau^{1/2}$ (s)	*A*	Std *A*	*C*	std *C*	χ^2	*D* (µm^2 s^{-1})
1	1.0	1.0	2.17	0.13	1.50	0.09	0.76	0.006	0.20	0.00	0.009	0.037
2	1.0	1.0	1.85	0.26	1.28	0.18	0.90	0.135	0.17	0.14	0.011	0.043
3	1.0	1.0	1.65	0.23	1.14	0.16	1.00	0.164	0.04	0.17	0.011	0.048
4	1.0	1.0	1.66	0.25	1.15	0.17	0.88	0.150	0.11	0.15	0.010	0.048
5	1.0	1.0	1.98	0.25	1.37	0.17	0.94	0.125	0.09	0.13	0.011	0.040
6	1.0	1.0	2.21	0.13	1.53	0.09	0.78	0.006	0.20	0.00	0.009	0.036
7	1.0	1.0	1.78	0.28	1.24	0.19	0.78	0.133	0.21	0.13	0.009	0.044
8	1.0	1.0	1.83	0.21	1.27	0.15	0.97	0.123	0.02	0.12	0.008	0.043
9	1.0	1.0	2.25	0.27	1.56	0.19	0.73	0.087	0.24	0.09	0.008	0.035
10	1.0	1.0	2.76	0.34	1.91	0.24	0.55	0.061	0.36	0.06	0.006	0.029
			**Avg τ (s)**	**1.40**	**Std τ (s)**	**0.237**			**Avg *D***	**0.040**	**Std *D***	**0.0062**

Supplementary Table 7. Concentrations of U15 in the dilute phase, in the cycling experiment (Figure 4) as measured by fluorimetry. All RNA values are in nM.

	$[U15]_{dilute\ phase}$ (nM)			
**Cycle**	**Trial 1**	**Trial 2**	**Average**	**Standard deviation**
**0**	2.1	1.3	1.7	0.4
**1**	2.0	1.6	1.8	0.2
**2**	1.6	1.2	1.4	0.2
**3**	1.4	1.6	1.5	0.1
**4**	1.5	1.1	1.3	0.2
**5**	1.5	1.5	1.5	0.0

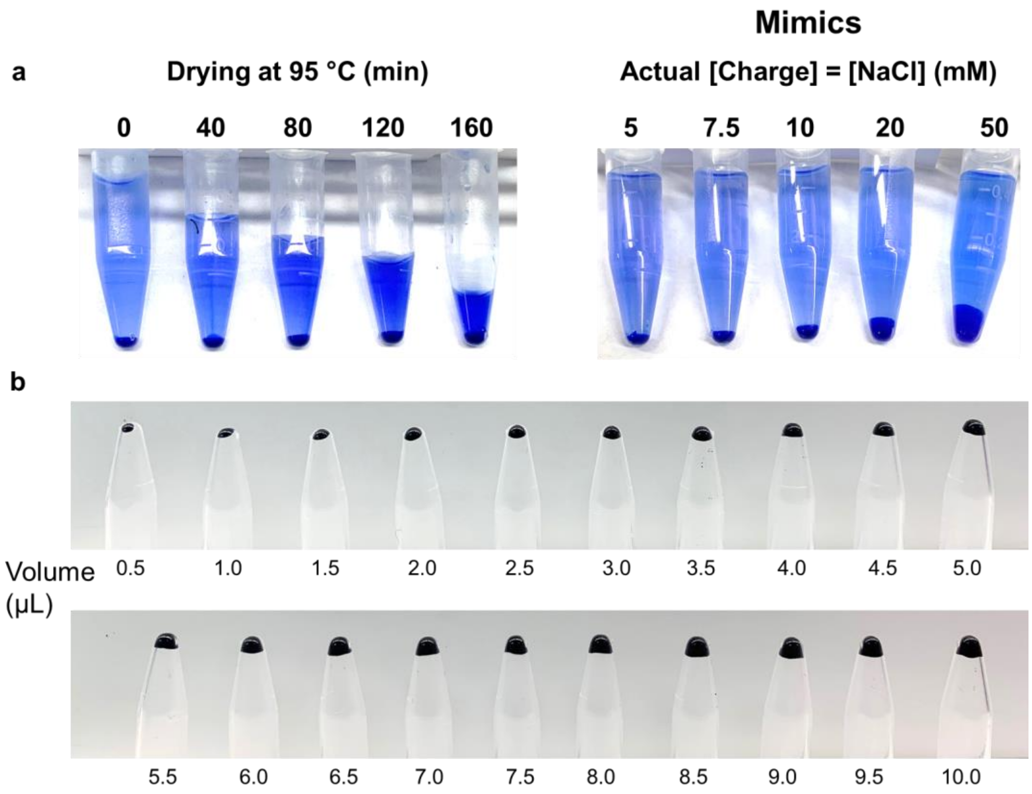

Supplementary Figure 22. a) PDADMA/PAA coacervate samples dried (left) and prepared at compositions that mimic the dried samples (right), with bromophenol blue added to show the two phases. b) Visual calibration curve prepared with bromophenol blue solutions used to estimate the coacervate volume.

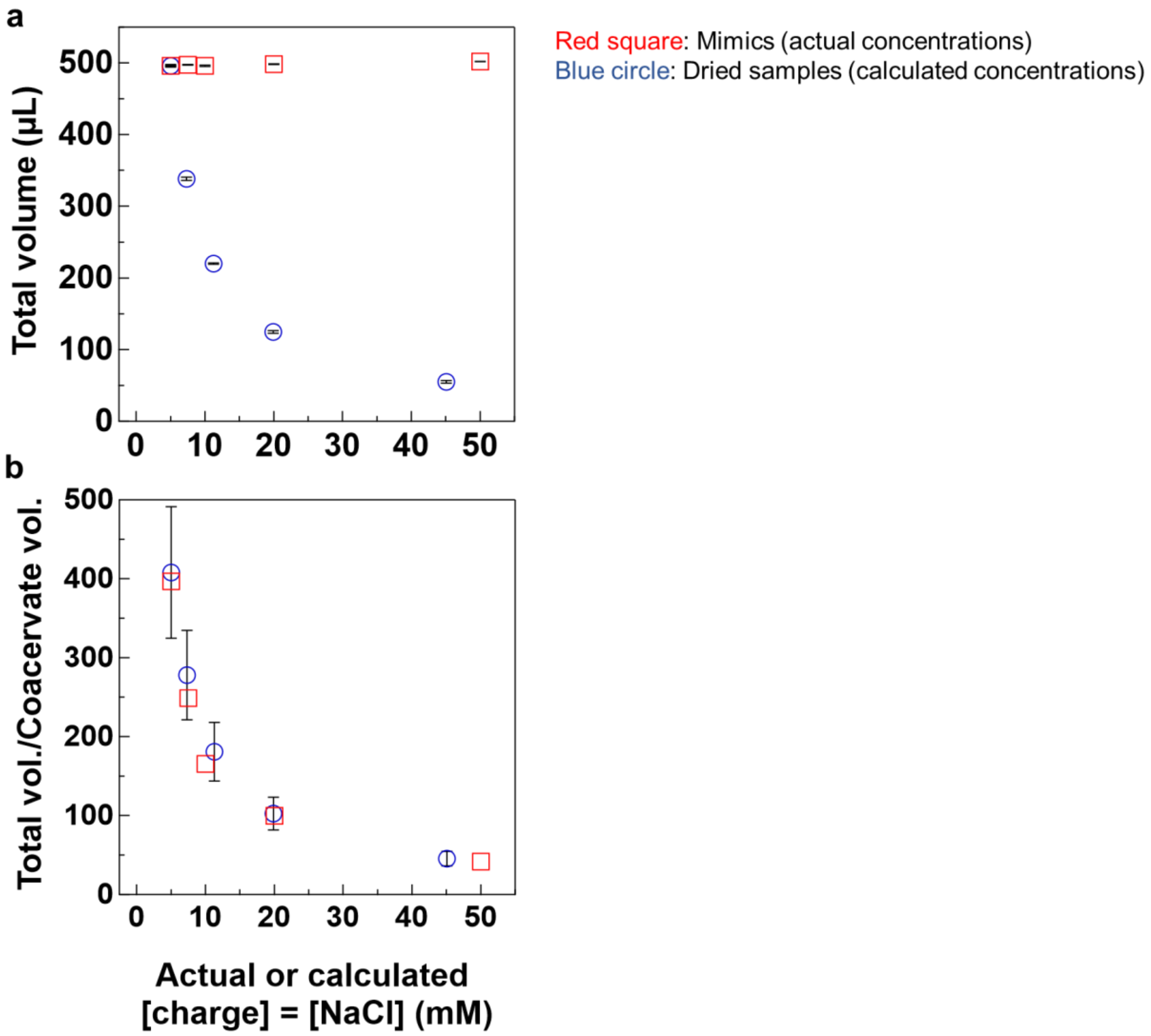

Supplementary Figure 23. a) Total volumes of dried and mimics samples obtained by weighing the PDADMA/PAA coacervates in tubes. Means and standard deviations are obtained from 3 samples for dried ones, and 2 for mimics. b) Ratio of Total solution volume/Coacervate volume in dried and mimics samples. The coacervate volume is estimated from the visual calibration curve in Supplementary Figure 22. Error bars in (b) were obtained by estimating the difference that 0.25 μL in volume would cause. This error value was chosen because it was difficult to visually estimate this fraction of volume.

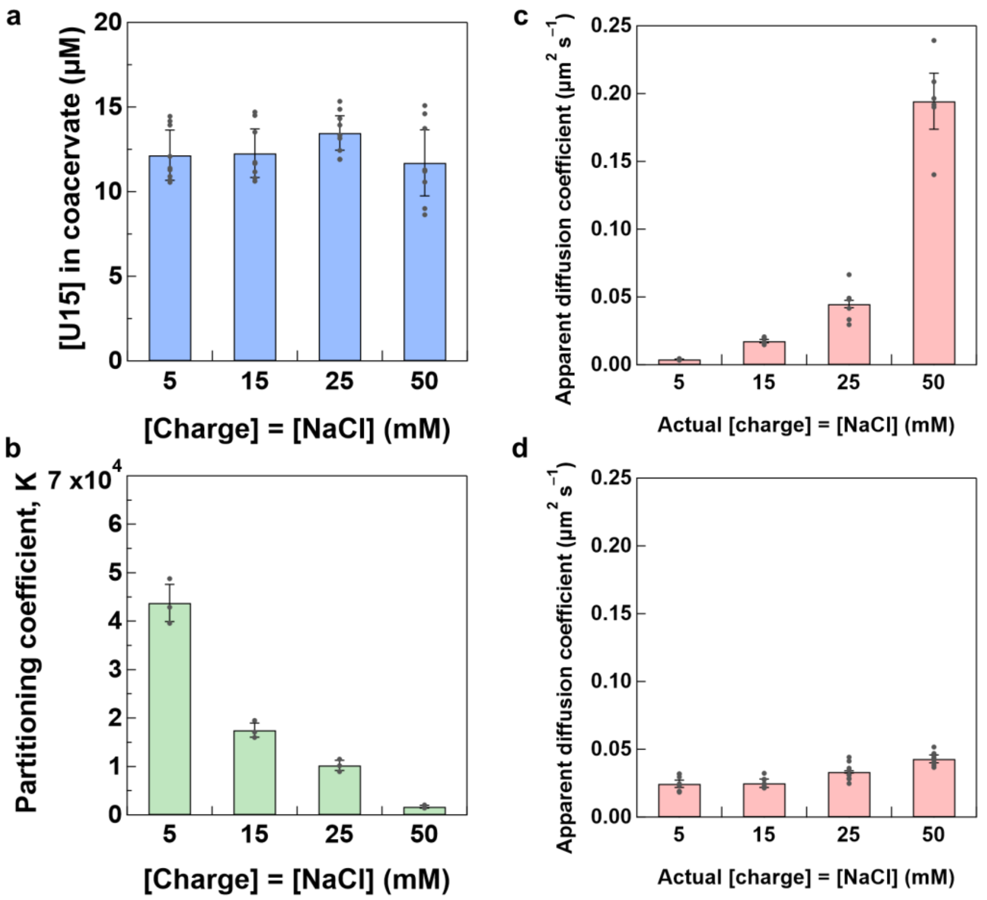

Supplementary Figure 24. a) Concentrations of Alexa 647-U15 within different PDADMA/PAA coacervate compositions that mimic the initial concentration as well as the ones reached at various time points of the drying experiment. The HEPES and $MgCl_2$ concentrations started at 2.5 and 0.43 mM, respectively, and were increased by the same factor as the polymer and NaCl concentrations. Individual data points (grey markers) are obtained from averages of various droplets in one image – and not individual droplets. b) The partitioning coefficient, taken as $[U15]_{coacervate\ phase}/[U15]_{dilute\ phase}$, in the same "mimics" samples. c) The apparent diffusion coefficients obtained from FRAP experiments over whole coacervate droplets and d) over partial 1-μm diameter regions in the same mimics samples. U15 was added in concentrations that matched the increase in the components' concentrations starting with 0.02 μM. Partitioning means and standard deviations are obtained from 3 samples with analysis of 15 droplets per sample over 3 images. FRAP means and standard deviations are obtained from 3 samples with at least 2 droplets/sample and 3 bleached areas/sample for whole-droplet and partial-droplet analysis, respectively.

Supplementary Table 8. Fitting parameters for whole droplet FRAP of PDADMA/PAA coacervate mimics samples.

5 mM samples

Trial	ROI (μm)	Bckgd (μm)	τ (s)	std τ (s)	$\tau^{1/2}$ (s)	std $\tau^{1/2}$ (s)	A	Std A	C	std C	χ^2	D (μm^2 s^{-1})
1	5.70	6.30	640.2	5.4	443.7	3.7	0.96	0.003	0.14	0.003	0.00046	0.004
2	6.20	6.30	768.3	7.9	532.5	5.5	0.81	0.003	0.16	0.002	0.00030	0.004
3	6.40	5.50	777.3	7.3	538.8	5.1	0.93	0.003	0.11	0.003	0.00032	0.004
4	6.40	5.50	707.2	6.9	490.2	4.8	0.85	0.003	0.14	0.003	0.00034	0.005
5	5.60	5.50	756.4	7.9	524.3	5.5	0.93	0.003	0.11	0.003	0.00041	0.003
6	5.60	5.50	610.8	6.2	423.4	4.3	0.80	0.003	0.14	0.003	0.00040	0.004
			**Avg $\tau^{1/2}$ (s)**	**492.2**	**Std $\tau^{1/2}$ (s)**	**48.8**			**Avg *D***	**0.004**	**Std *D***	**0.0004**

15 mM samples

Trial	ROI (μm)	Bckgd (μm)	τ (s)	std τ (s)	$\tau^{1/2}$ (s)	std $\tau^{1/2}$ (s)	A	Std A	C	std C	χ^2	D (μm^2 s^{-1})
1	8.00	7.40	253.3	1.6	175.6	1.1	0.86	0.003	0.00	0.003	0.00032	0.020
2	8.00	7.40	309.6	1.9	214.6	1.3	1.00	0.003	0.01	0.003	0.00037	0.016
3	7.90	7.50	300.7	1.8	208.4	1.2	0.89	0.002	0.03	0.003	0.00027	0.016
4	7.90	7.20	239.8	1.6	166.2	1.1	0.90	0.003	0.07	0.003	0.00037	0.021
5	8.00	7.50	341.5	2.2	236.7	1.5	0.98	0.003	0.06	0.003	0.00039	0.015
6	8.40	7.50	329.8	3.3	228.6	2.3	1.00	0.004	0.08	0.005	0.00102	0.017
			**Avg $\tau^{1/2}$ (s)**	**205.0**	**Std $\tau^{1/2}$ (s)**	**28.4**			**Avg *D***	**0.018**	**Std *D***	**0.0023**

25 mM samples

Trial	ROI (μm)	Bckgd (μm)	τ (s)	std τ (s)	$\tau^{1/2}$ (s)	std $\tau^{1/2}$ (s)	A	Std A	C	std C	χ^2	D (μm^2 s^{-1})
1	7.00	7.00	93.2	0.7	64.6	0.5	0.91	0.005	0.00	0.005	0.00050	0.042
2	6.70	7.00	74.1	0.6	51.4	0.4	0.98	0.006	0.00	0.006	0.00046	0.048
3	7.00	9.00	131.3	3.0	91.0	2.1	1.15	0.014	0.10	0.015	0.00542	0.030
4	7.40	8.50	65.3	0.5	45.3	0.4	0.97	0.005	0.00	0.005	0.00033	0.067
5	7.00	7.60	116.9	1.1	81.1	0.7	1.00	0.005	0.00	0.005	0.00063	0.033
6	7.40	7.40	88.4	0.7	61.3	0.5	0.88	0.005	0.00	0.005	0.00038	0.049
			**Avg $\tau^{1/2}$ (s)**	**65.8**	**Std $\tau^{1/2}$ (s)**	**17.4**			**Avg *D***	**0.045**	**Std *D***	**0.013**

50 mM samples

Trial	ROI (μm)	Bckgd (μm)	τ (s)	std τ (s)	$\tau^{1/2}$ (s)	std $\tau^{1/2}$ (s)	A	Std A	C	std C	χ^2	D (μm^2 s^{-1})
1	8.20	8.60	25.6	0.4	17.7	0.3	0.97	0.013	0.00	0.013	0.00074	0.209
2	8.40	8.20	28.5	0.4	19.7	0.3	1.00	0.010	0.02	0.010	0.00050	0.197
3	7.90	7.10	20.7	0.4	14.4	0.3	1.00	0.014	0.00	0.014	0.00062	0.239
4	8.20	7.75	28.1	0.5	19.5	0.3	1.00	0.013	0.00	0.013	0.00078	0.190
5	8.20	8.00	27.8	0.6	19.3	0.4	1.00	0.016	0.00	0.016	0.00125	0.192
6	7.80	8.00	34.4	0.5	23.9	0.3	1.00	0.009	0.04	0.010	0.00063	0.140
			**Avg $\tau^{1/2}$ (s)**	**19.1**	**Std $\tau^{1/2}$ (s)**	**3.1**			**Avg *D***	**0.194**	**Std *D***	**0.0322**

Supplementary Table 9. Fitting parameters for partial droplet FRAP of PDADMA/PAA coacervate mimics samples.

5 mM samples

Trial	ROI (μm)	Bckgd (μm)	τ (s)	std τ (s)	$\tau^{1/2}$ (s)	std $\tau^{1/2}$ (s)	*A*	Std *A*	*C*	std *C*	χ^2	*D* (μm^2 s^{-1})
1	1.0	1.0	4.22	0.45	2.93	0.31	0.79	0.059	0.15	0.06	0.014	0.019
2	1.0	1.0	3.38	0.36	2.34	0.25	0.87	0.074	0.16	0.08	0.014	0.023
3	1.0	1.0	3.23	0.35	2.24	0.24	0.81	0.072	0.11	0.07	0.012	0.025
4	1.0	1.0	2.68	0.34	1.86	0.23	1.00	0.113	0.04	0.12	0.020	0.030
5	1.0	1.0	4.33	0.37	3.00	0.26	0.82	0.049	0.09	0.05	0.010	0.018
6	1.0	1.0	3.39	0.31	2.35	0.21	0.96	0.070	0.01	0.07	0.013	0.023
7	1.0	1.0	2.49	0.33	1.73	0.23	1.00	0.125	0.03	0.13	0.020	0.032
8	1.0	1.0	2.49	0.34	1.72	0.24	0.99	0.127	0.00	0.13	0.021	0.032
9	1.0	1.0	4.14	0.48	2.87	0.33	0.85	0.071	0.23	0.07	0.020	0.019
			**Avg $\tau^{1/2}$ (s)**	**2.34**	**Std $\tau^{1/2}$ (s)**	**0.507**			**Avg *D***	**0.025**	**Std *D***	**0.0054**

15 mM samples

Trial	ROI (μm)	Bckgd (μm)	τ (s)	std τ (s)	$\tau^{1/2}$ (s)	std $\tau^{1/2}$ (s)	*A*	Std *A*	*C*	std *C*	χ^2	*D* (μm^2 s^{-1})
1	1.0	1.0	3.60	0.21	2.50	0.15	0.99	0.011	0.00	0.00	0.020	0.022
2	1.0	1.0	3.53	0.22	2.45	0.15	0.99	0.011	0.00	0.00	0.023	0.022
3	1.0	1.0	3.70	0.18	2.56	0.13	1.00	0.009	0.00	0.00	0.015	0.021
4	1.0	1.0	3.65	0.49	2.53	0.34	0.78	0.081	0.18	0.08	0.020	0.022
5	1.0	1.0	3.23	0.02	2.24	0.02	1.00	0.012	0.00	0.00	0.027	0.025
6	1.0	1.0	2.87	0.19	1.99	0.13	0.95	0.010	0.00	0.00	0.020	0.028
7	1.0	1.0	2.45	0.16	1.70	0.11	0.95	0.009	0.00	0.00	0.018	0.032
8	1.0	1.0	2.85	0.02	1.97	0.01	0.88	0.009	0.00	0.00	0.017	0.028
9	1.0	1.0	3.26	0.23	2.26	0.16	0.86	0.010	0.00	0.00	0.020	0.024
			**Avg $\tau^{1/2}$ (s)**	**2.24**	**Std $\tau^{1/2}$ (s)**	**0.301**			**Avg *D***	**0.025**	**Std *D***	**0.0037**

25 mM samples

Trial	ROI (μm)	Bckgd (μm)	τ (s)	std τ (s)	$\tau^{1/2}$ (s)	std $\tau^{1/2}$ (s)	*A*	Std *A*	*C*	std *C*	χ^2	*D* (μm^2 s^{-1})
1	1.0	1.0	3.21	0.64	2.22	0.44	1.00	0.189	0.12	0.19	0.045	0.025
2	1.0	1.0	1.92	0.35	1.33	0.25	1.00	0.195	0.01	0.20	0.025	0.041
3	1.0	1.0	2.55	0.36	1.77	0.25	1.00	0.131	0.04	0.13	0.024	0.031
4	1.0	1.0	2.21	0.34	1.53	0.23	0.95	0.143	0.00	0.15	0.020	0.036
5	1.0	1.0	2.54	0.35	1.76	0.24	1.00	0.128	0.00	0.13	0.022	0.031
6	1.0	1.0	2.30	0.33	1.59	0.23	1.00	0.140	0.01	0.14	0.021	0.035
7	1.0	1.0	1.80	0.37	1.25	0.25	1.00	0.224	0.02	0.23	0.027	0.044
8	1.0	1.0	2.82	0.40	1.96	0.28	0.98	0.122	0.00	0.12	0.026	0.028
9	1.0	1.0	2.77	0.39	1.92	0.27	1.00	0.123	0.07	0.13	0.026	0.029
			**Avg $\tau^{1/2}$ (s)**	**1.70**	**Std $\tau^{1/2}$ (s)**	**0.312**			**Avg *D***	**0.033**	**Std *D***	**0.0063**

50 mM samples

Trial	ROI (μm)	Bckgd (μm)	τ (s)	std τ (s)	$\tau^{1/2}$ (s)	std $\tau^{1/2}$ (s)	*A*	Std *A*	*C*	std *C*	χ^2	*D* (μm^2 s^{-1})
1	1.0	1.0	2.07	0.17	1.44	0.12	1.00	0.011	0.00	0.00	0.027	0.038
2	1.0	1.0	1.86	0.33	1.29	0.23	1.00	0.191	0.04	0.19	0.022	0.043
3	1.0	1.0	1.88	0.36	1.31	0.25	0.99	0.206	0.02	0.21	0.026	0.042
4	1.0	1.0	1.69	0.32	1.17	0.22	0.99	0.210	0.00	0.21	0.020	0.047
5	1.0	1.0	1.53	0.35	1.06	0.24	1.00	0.270	0.03	0.27	0.024	0.052
6	1.0	1.0	1.97	0.33	1.37	0.23	1.00	0.178	0.00	0.18	0.022	0.040
7	1.0	1.0	1.78	0.32	1.23	0.22	0.99	0.196	0.00	0.20	0.020	0.045
8	1.0	1.0	1.83	0.34	1.27	0.24	1.00	0.206	0.01	0.21	0.024	0.043
9	1.0	1.0	2.17	0.35	1.50	0.24	1.00	0.162	0.03	0.16	0.024	0.037
			**Avg $\tau^{1/2}$ (s)**	**1.29**	**Std $\tau^{1/2}$ (s)**	**0.133**			**Avg *D***	**0.043**	**Std *D***	**0.0046**

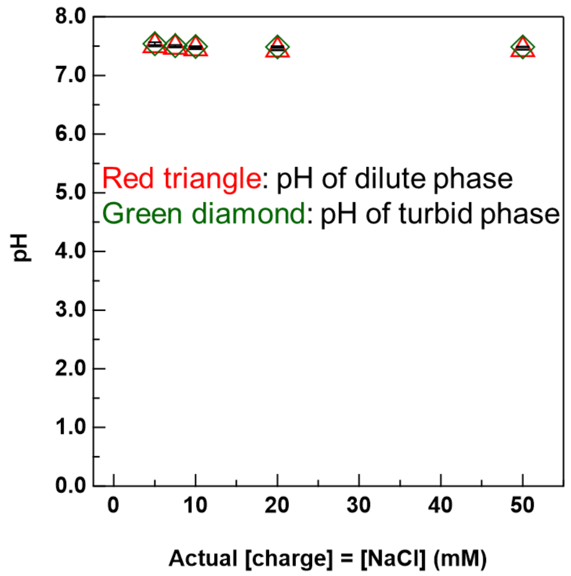

Supplementary Figure 25. pH of PDADMA/PAA coacervate mimics samples measured for both the dilute phase and the turbid overall mixture. Means and standard deviations are obtained from 2 samples.

Supplementary Table 10. PDADMA/PAA coacervate sample details for the SAXS experiment.

All in M	A	B	C	D	E	F
[Polymer]	0.05	0.055	0.06	0.065	0.07	0.075
[NaCl]	0.05	0.055	0.06	0.065	0.07	0.075
[HEPES]	0.025	0.0275	0.03	0.0325	0.035	0.0375
[$MgCl_2$]	0.0043	0.00473	0.00516	0.00559	0.00602	0.00645

All in M	G	H	I	J	K
[Polymer]	0.08	0.085	0.09	0.095	0.1
[NaCl]	0.08	0.085	0.09	0.095	0.1
[HEPES]	0.04	0.0425	0.045	0.0475	0.05
[$MgCl_2$]	0.00688	0.00731	0.00774	0.00817	0.0086

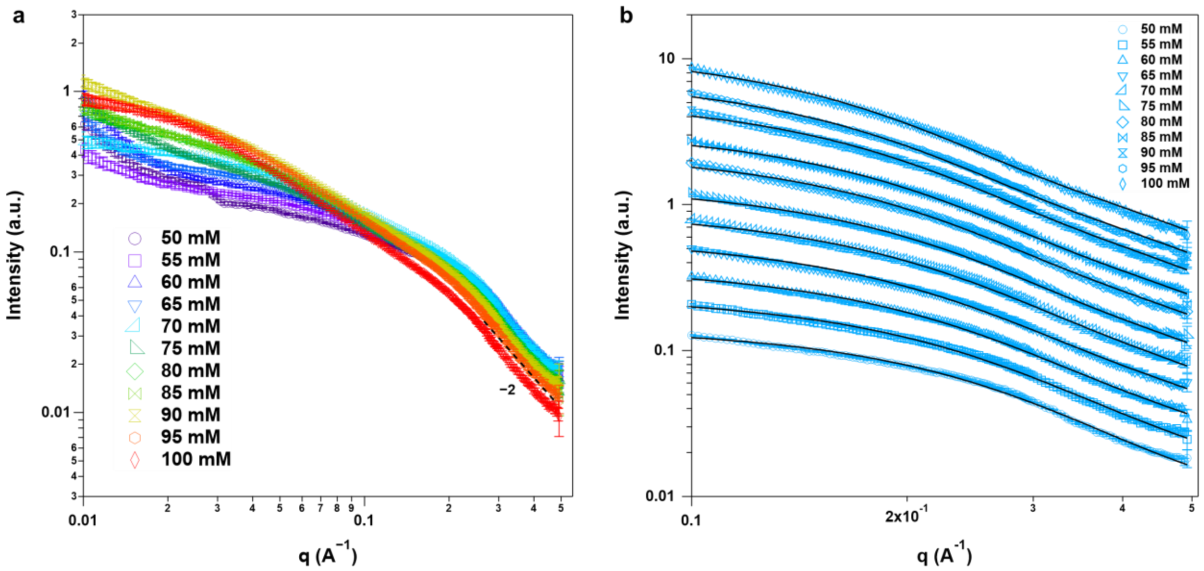

Supplementary Figure 26. a) Small-angle X-ray scattering (SAXS) plots, not shifted vertically for clarity, of PDADMA/PAA complex coacervate samples prepared at 50 – 100 mM polymer charge and NaCl, with HEPES ranging between 25 and 50 mM, and $MgCl_2$ between 4.3 and 8.6 mM for the same samples. b) SAXS fits of the high-q region obtained from PDADMA/PAA coacervates prepared at concentrations that range between 50- and 100-mM charge (polymer with respect to the monomer) and NaCl, between 25 and 50 mM HEPES, and 4.3 and 8.6 mM $MgCl_2$. The black lines are fits (Unified fit) to the experimental data which are shown with markers.

Supplementary Table 11. Fitting parameters for the Unified fit[24] of the SAXS plots of PDADMA/PAA samples prepared at concentrations ranging between 50 and 100 mM NaCl and charge (polymer concentration with respect to the monomer) with increasing concentrations of $MgCl_2$ and HEPES. R_g refers to the measured length scale from the broad peak at 0.3 Å^{-1}, which is the correlation length, and not the overall polymer size.

[Pol]=[NaCl] (mM)	*G*	Error, *G*	R_g (Å)	Error, R_g	*B*	Error, *B*	*P*
50	0.142	0.002	7.02	0.02	0.00362	0.00039	2.08
55	0.163	0.002	7.42	0.02	0.00435	0.00030	2.08
60	0.176	0.001	7.83	0.02	0.00455	0.00024	1.99
65	0.190	0.001	8.15	0.02	0.00440	0.00018	2.05
70	0.200	0.001	8.28	0.02	0.00420	0.00017	2.09
75	0.171	0.001	8.49	0.03	0.00354	0.00014	2.03
80	0.197	0.001	8.94	0.03	0.00367	0.00011	2.04
85	0.192	0.001	9.14	0.03	0.00349	0.00009	2.03
90	0.214	0.001	9.52	0.03	0.00337	0.00008	2.06
95	0.204	0.001	10.01	0.04	0.00313	0.00006	2.01
100	0.175	0.001	10.38	0.04	0.00251	0.00005	1.99

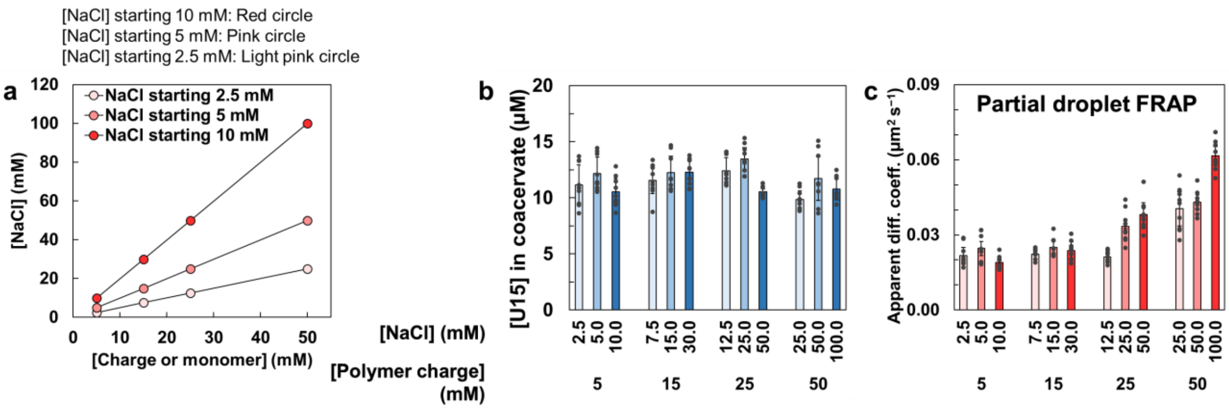

Supplementary Figure 27. Changing the directionality of movements on the phase diagram. a) Plot showing the change in salt concentrations in experiments that represent different compositions on the PDADMA/PAA phase diagram. HEPES and $MgCl_2$ were added at 2.5 and 0.43 mM initially and increased in all samples with the same factor of increase of NaCl on the y-axis. b) U15 concentrations within the PDADMA/PAA coacervate phase. c) Partial droplet FRAP of the same samples. Supplementary Table 13 includes all the fitting parameters. Partitioning means and standard deviations are obtained from 3 samples each with analysis of 15 droplets/sample over 3 confocal images. Means and standard deviations in FRAP are obtained from 3 samples with at least 3 bleached areas each. Individual data points are shown as grey markers.

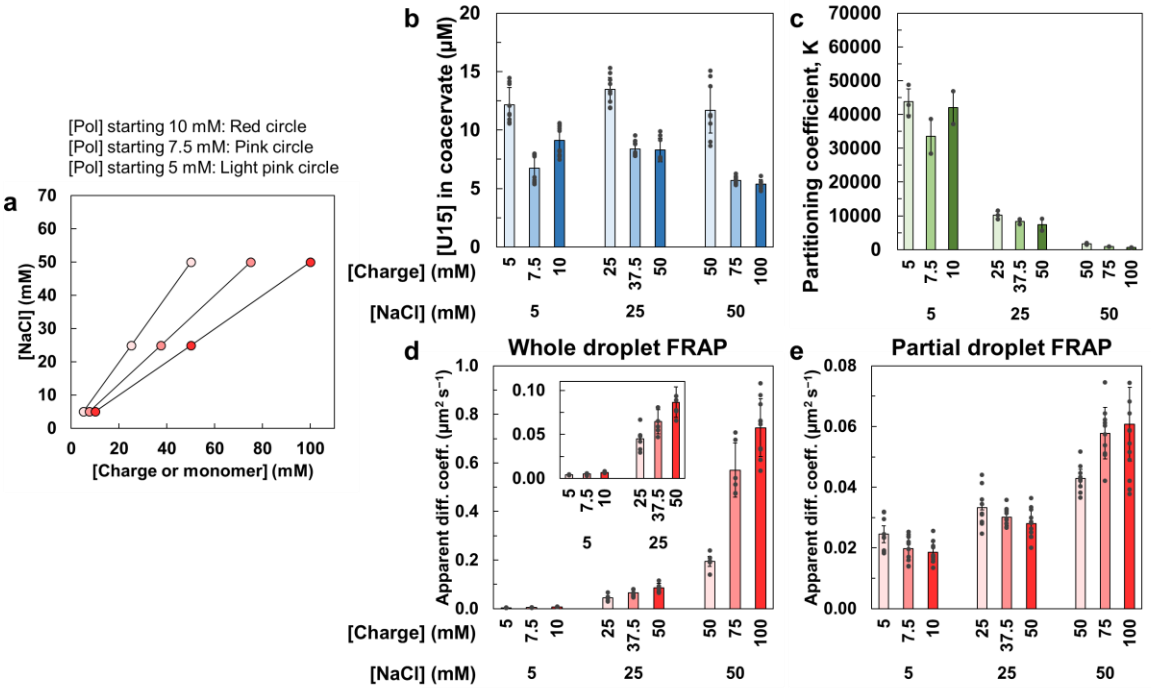

Supplementary Figure 28. a) Plot showing the change in polymer charge concentrations in experiments that represent different compositions on the PDADMA/PAA coacervate phase diagram. HEPES and $MgCl_2$ were added at 2.5 and 0.43 mM initially and increased in all samples with the same factor of increase of [polymer] on the x-axis. b) U15 concentrations within the PDADMA/PAA coacervate and c) partitioning coefficient, taken as $[U15]_{coacervate\ phase}/[U15]_{dilute\ phase}$, with different concentrations of polymer charge for the same salt concentrations. d) Whole droplet FRAP experiments of the same range of samples. The inset corresponds to a zoom-in on the low concentration samples. e) Partial droplet FRAP of the same samples. Supplementary Tables 14 and 15 (below) include all the fitting parameters. The added U15 was increased between 0.03 – 0.3 μM and between 0.04 – 0.4 μM for the increasing [polymer charge] samples starting with 7.5 mM and 10 mM for the lowest concentrations, respectively. Partitioning means and standard deviations are obtained from 2 samples each with analysis of at least 15 droplets/sample over at least 3 confocal images. Means and standard deviations in whole-droplet FRAP are obtained from 2 samples with at least 3 bleached droplets each. In partial-droplet FRAP, means and standard deviations were from 2 samples with at least 5 bleached areas each. Individual data points are shown as grey markers.

Supplementary Table 12. Starting 2.5 and 10 mM NaCl mimics – whole droplet FRAP fitting parameters.

[Charge] = 5 mM, [NaCl] = 2.5 mM

Trial	ROI (μm)	Bckgd (μm)	τ (s)	std τ (s)	$\tau^{1/2}$ (s)	std $\tau^{1/2}$ (s)	A	Std A	C	std C	χ^2	D (μm^2 s^{-1})
1	6.20	6.50	959.0	23.4	664.7	16.2	0.89	0.010	0.05	0.003	0.00049	0.0032
2	6.40	6.20	942.1	19.6	653.0	13.6	0.98	0.008	0.06	0.003	0.00047	0.0034
3	5.80	6.20	762.2	21.6	528.3	15.0	0.71	0.006	0.06	0.005	0.00119	0.0035
4	5.80	5.90	811.7	25.9	562.7	18.0	1.00	0.010	0.07	0.007	0.00267	0.0033
5	5.60	4.60	543.3	12.8	376.6	8.9	0.79	0.006	0.13	0.007	0.00178	0.0046
6	6.50	4.80	439.9	6.3	304.9	4.4	0.72	0.004	0.12	0.004	0.00069	0.0076
			**Avg $\tau^{1/2}$ (s)**	**515.0**	**Std $\tau^{1/2}$ (s)**	**146.4**			**Avg D**	**0.0043**	**Std D**	**0.0017**

[Charge] = 5 mM, [NaCl] = 10 mM

Trial	ROI (μm)	Bckgd (μm)	τ (s)	std τ (s)	$\tau^{1/2}$ (s)	std $\tau^{1/2}$ (s)	A	Std A	C	std C	χ^2	D (μm^2 s^{-1})
1	6.00	7.30	644.7	6.9	446.9	4.8	1.00	0.003	0.18	0.003	0.00037	0.004
2	6.20	6.60	526.9	5.0	365.2	3.5	0.89	0.003	0.21	0.003	0.00033	0.006
3	6.60	6.40	598.9	5.4	415.2	3.8	0.95	0.003	0.08	0.002	0.00037	0.006
4	6.10	6.30	521.0	6.3	361.1	4.4	1.00	0.004	0.13	0.004	0.00069	0.006
5	6.30	7.40	648.6	6.9	449.5	4.8	0.96	0.003	0.12	0.003	0.00033	0.005
6	6.00	6.80	418.9	4.7	290.4	3.2	0.92	0.004	0.13	0.004	0.00066	0.007
			**Avg $\tau^{1/2}$ (s)**	**388.0**	**Std $\tau^{1/2}$ (s)**	**61.3**			**Avg D**	**0.0056**	**Std D**	**0.00083**

[Charge] = 15 mM, [NaCl] = 7.5 mM

Trial	ROI (μm)	Bckgd (μm)	τ (s)	std τ (s)	$\tau^{1/2}$ (s)	std $\tau^{1/2}$ (s)	A	Std A	C	std C	χ^2	D (μm^2 s^{-1})
1	7.30	6.20	214.7	1.5	148.8	1.1	0.82	0.003	0.14	0.003	0.00035	0.020
2	7.40	6.00	269.8	2.2	187.0	1.5	0.91	0.003	0.15	0.003	0.00034	0.016
3	6.40	6.60	257.1	2.1	178.2	1.4	0.77	0.003	0.14	0.003	0.00025	0.013
4	6.80	8.20	252.4	1.8	174.9	1.3	0.83	0.002	0.18	0.003	0.00024	0.015
5	6.50	5.20	170.8	1.6	118.4	1.1	0.75	0.004	0.15	0.004	0.00036	0.020
6	6.50	5.50	177.6	1.6	123.1	1.1	0.80	0.004	0.21	0.004	0.00041	0.019
			**Avg $\tau^{1/2}$ (s)**	**155.1**	**Std $\tau^{1/2}$ (s)**	**29.5**			**Avg D**	**0.017**	**Std D**	**0.0029**

[Charge] = 15 mM, [NaCl] = 30 mM

Trial	ROI (μm)	Bckgd (μm)	τ (s)	std τ (s)	$\tau^{1/2}$ (s)	std $\tau^{1/2}$ (s)	A	Std A	C	std C	χ^2	D (μm^2 s^{-1})
1	7.40	8.70	192.9	1.4	133.7	1.0	0.98	0.003	0.04	0.003	0.00032	0.023
2	7.10	8.50	142.5	1.1	98.8	0.8	0.93	0.003	0.08	0.003	0.00028	0.028
3	7.00	6.00	134.3	1.1	93.1	0.8	0.83	0.003	0.04	0.003	0.00026	0.029
4	7.80	6.20	141.7	1.2	98.2	0.8	0.71	0.002	0.07	0.003	0.00019	0.034
5	7.10	6.20	121.1	1.0	84.0	0.7	0.85	0.003	0.14	0.004	0.00032	0.033
6	7.40	6.10	114.6	1.1	79.4	0.8	0.83	0.004	0.09	0.004	0.00038	0.038
			**Avg $\tau^{1/2}$ (s)**	**97.9**	**Std $\tau^{1/2}$ (s)**	**19.2**			**Avg D**	**0.031**	**Std D**	**0.0054**

Supplementary Table 12 (continued)

[Charge] = 25 mM, [NaCl] = 12.5 mM

Trial	ROI (μm)	Bckgd (μm)	τ (s)	std τ (s)	$\tau^{1/2}$ (s)	std $\tau^{1/2}$ (s)	A	Std A	C	std C	χ^2	D (μm^2 s^{-1})
1	7.00	7.40	145.6	1.4	100.9	1.0	0.92	0.006	0.13	0.006	0.00067	0.027
2	6.80	6.70	111.2	0.7	77.1	0.5	0.93	0.004	0.11	0.004	0.00036	0.033
3	7.40	6.20	90.5	0.7	62.7	0.5	0.84	0.004	0.11	0.004	0.00028	0.048
4	6.80	6.70	69.4	0.7	48.1	0.5	0.79	0.005	0.07	0.005	0.00038	0.053
5	6.60	8.10	92.3	0.7	64.0	0.5	0.79	0.004	0.11	0.004	0.00027	0.037
6	7.50	8.30	99.2	0.6	68.7	0.4	0.82	0.003	0.10	0.003	0.00017	0.045
			**Avg $\tau^{1/2}$ (s)**	**70.3**	**Std $\tau^{1/2}$ (s)**	**17.7**			**Avg D**	**0.041**	**Std D**	**0.0099**

[Charge] = 25 mM, [NaCl] = 50 mM

Trial	ROI (μm)	Bckgd (μm)	τ (s)	std τ (s)	$\tau^{1/2}$ (s)	std $\tau^{1/2}$ (s)	A	Std A	C	std C	χ^2	D (μm^2 s^{-1})
1	8.10	7.80	58.8	0.4	40.7	0.3	0.90	0.003	0.08	0.003	0.00017	0.089
2	8.10	7.10	60.8	0.4	42.2	0.3	0.98	0.003	0.09	0.003	0.00021	0.086
3	7.20	8.00	50.1	0.4	34.7	0.3	0.95	0.004	0.17	0.004	0.00025	0.082
4	7.60	6.30	43.6	0.4	30.3	0.2	0.85	0.004	0.08	0.004	0.00022	0.105
5	7.40	7.00	29.8	0.3	20.6	0.2	0.89	0.006	0.08	0.006	0.00027	0.146
6	8.10	7.00	37.2	0.3	25.8	0.2	0.87	0.005	0.11	0.005	0.00024	0.140
			**Avg $\tau^{1/2}$ (s)**	**32.4**	**Std $\tau^{1/2}$ (s)**	**8.5**			**Avg D**	**0.108**	**Std D**	**0.0284**

[Charge] = 50 mM, [NaCl] = 25 mM

Trial	ROI (μm)	Bckgd (μm)	τ (s)	std τ (s)	$\tau^{1/2}$ (s)	std $\tau^{1/2}$ (s)	A	Std A	C	std C	χ^2	D (μm^2 s^{-1})
1	8.30	7.60	29.0	0.2	20.1	0.1	0.95	0.003	0.02	0.003	0.00022	0.188
2	8.40	8.40	22.9	0.1	15.9	0.1	0.93	0.004	0.06	0.004	0.00022	0.245
3	7.30	6.60	18.6	0.1	12.9	0.1	0.95	0.005	0.02	0.005	0.00027	0.227
4	8.20	6.30	23.9	0.2	16.6	0.1	0.97	0.004	0.01	0.005	0.00031	0.223
5	7.30	6.90	16.0	0.1	11.1	0.1	1.00	0.006	0.01	0.007	0.00033	0.264
6	7.80	6.70	17.8	0.2	12.3	0.1	0.90	0.007	0.00	0.007	0.00047	0.271
			**Avg $\tau^{1/2}$ (s)**	**14.8**	**Std $\tau^{1/2}$ (s)**	**3.3**			**Avg D**	**0.236**	**Std D**	**0.0303**

[Charge] = 50 mM, [NaCl] = 100 mM

Trial	ROI (μm)	Bckgd (μm)	τ (s)	std τ (s)	$\tau^{1/2}$ (s)	std $\tau^{1/2}$ (s)	A	Std A	C	std C	χ^2	D (μm^2 s^{-1})
1	7.50	7.10	10.8	0.3	7.5	0.2	1.00	0.020	0.01	0.021	0.00141	0.413
2	8.00	8.40	12.8	0.3	8.9	0.2	1.00	0.018	0.02	0.019	0.00161	0.397
3	7.80	7.20	11.4	0.2	7.9	0.1	1.00	0.015	0.00	0.016	0.00091	0.424
4	7.80	7.80	11.3	0.2	7.8	0.2	1.00	0.016	0.00	0.017	0.00098	0.428
5	7.80	7.40	8.7	0.2	6.0	0.1	0.89	0.019	0.00	0.019	0.00075	0.554
6	7.80	7.80	8.5	0.2	5.9	0.1	0.98	0.021	0.00	0.021	0.00084	0.571
			**Avg $\tau^{1/2}$ (s)**	**7.3**	**Std $\tau^{1/2}$ (s)**	**1.2**			**Avg D**	**0.465**	**Std D**	**0.0768**

Supplementary Table 13. Starting 2.5 and 10 mM NaCl mimics – partial droplet FRAP fitting parameters.

[Charge] = 5 mM, [NaCl] = 2.5 mM

Trial	ROI (μm)	Bckgd (μm)	τ (s)	std τ (s)	$\tau^{1/2}$ (s)	std $\tau^{1/2}$ (s)	A	Std A	C	std C	χ^2	D (μm^2 s^{-1})
1	1.0	1.0	4.03	0.47	2.79	0.33	0.83	0.071	0.09	0.07	0.018	0.020
2	1.0	1.0	4.68	0.43	3.24	0.30	0.93	0.057	0.08	0.06	0.016	0.017
3	1.0	1.0	3.95	0.39	2.74	0.27	0.98	0.072	0.02	0.07	0.018	0.020
4	1.0	1.0	3.81	0.42	2.64	0.29	0.95	0.079	0.10	0.08	0.021	0.021
5	1.0	1.0	4.27	0.57	2.96	0.39	0.82	0.076	0.18	0.08	0.024	0.019
6	1.0	1.0	3.36	0.44	2.33	0.30	0.90	0.094	0.16	0.10	0.023	0.024
7	1.0	1.0	2.75	0.37	1.91	0.26	0.98	0.117	0.00	0.12	0.023	0.029
8	1.0	1.0	2.79	0.35	1.94	0.24	0.91	0.101	0.02	0.10	0.017	0.028
9	1.0	1.0	4.21	0.52	2.92	0.36	0.87	0.077	0.15	0.08	0.024	0.019
			**Avg $\tau^{1/2}$ (s)**	**2.61**	**Std $\tau^{1/2}$ (s)**	**0.460**			**Avg D**	**0.022**	**Std D**	**0.0043**

[Charge] = 5 mM, [NaCl] = 10 mM

Trial	ROI (μm)	Bckgd (μm)	τ (s)	std τ (s)	$\tau^{1/2}$ (s)	std $\tau^{1/2}$ (s)	A	Std A	C	std C	χ^2	D (μm^2 s^{-1})
1	1.0	1.0	3.93	0.49	2.72	0.34	0.64	0.058	0.31	0.06	0.012	0.020
2	1.0	1.0	4.45	0.51	3.08	0.35	0.76	0.060	0.29	0.06	0.016	0.018
3	1.0	1.0	4.76	0.54	3.30	0.37	0.67	0.050	0.33	0.05	0.012	0.017
4	1.0	1.0	3.74	0.40	2.59	0.28	0.85	0.070	0.20	0.07	0.016	0.021
5	1.0	1.0	4.53	0.47	3.14	0.32	0.83	0.058	0.23	0.06	0.016	0.018
6	1.0	1.0	3.94	0.46	2.73	0.32	0.72	0.061	0.20	0.06	0.013	0.020
7	1.0	1.0	3.30	0.42	2.29	0.29	0.71	0.074	0.19	0.08	0.013	0.024
8	1.0	1.0	4.91	0.59	3.41	0.41	0.77	0.060	0.27	0.06	0.019	0.016
9	1.0	1.0	4.61	0.50	3.20	0.34	0.79	0.057	0.25	0.06	0.015	0.017
			**Avg $\tau^{1/2}$ (s)**	**2.94**	**Std $\tau^{1/2}$ (s)**	**0.373**			**Avg D**	**0.019**	**Std D**	**0.0026**

[Charge] = 15 mM, [NaCl] = 7.5 mM

Trial	ROI (μm)	Bckgd (μm)	τ (s)	std τ (s)	$\tau^{1/2}$ (s)	std $\tau^{1/2}$ (s)	A	Std A	C	std C	χ^2	D (μm^2 s^{-1})
1	1.0	1.0	4.17	0.50	2.89	0.35	0.68	0.058	0.32	0.06	0.014	0.019
2	1.0	1.0	3.54	0.39	2.45	0.27	0.81	0.070	0.22	0.07	0.014	0.022
3	1.0	1.0	3.47	0.36	2.40	0.25	0.72	0.059	0.21	0.06	0.010	0.023
4	1.0	1.0	3.93	0.34	2.72	0.24	0.77	0.050	0.20	0.05	0.009	0.020
5	1.0	1.0	3.59	0.39	2.49	0.27	0.75	0.064	0.22	0.07	0.012	0.022
6	1.0	1.0	3.18	0.37	2.21	0.25	0.71	0.068	0.25	0.07	0.011	0.025
7	1.0	1.0	3.26	0.41	2.26	0.29	0.70	0.072	0.26	0.07	0.013	0.024
8	1.0	1.0	3.33	0.40	2.31	0.28	0.74	0.072	0.26	0.07	0.013	0.024
			**Avg $\tau^{1/2}$ (s)**	**2.47**	**Std $\tau^{1/2}$ (s)**	**0.235**			**Avg D**	**0.022**	**Std D**	**0.0020**

[Charge] = 15 mM, [NaCl] = 30 mM

Trial	ROI (μm)	Bckgd (μm)	τ (s)	std τ (s)	$\tau^{1/2}$ (s)	std $\tau^{1/2}$ (s)	A	Std A	C	std C	χ^2	D (μm^2 s^{-1})
1	1.0	1.0	3.90	0.45	2.70	0.31	0.83	0.071	0.23	0.07	0.017	0.020
2	1.0	1.0	3.28	0.37	2.27	0.26	0.84	0.077	0.14	0.08	0.015	0.024
3	1.0	1.0	3.90	0.42	2.70	0.29	0.72	0.057	0.21	0.06	0.011	0.020
4	1.0	1.0	2.87	0.33	1.99	0.23	0.90	0.091	0.10	0.09	0.015	0.028
5	1.0	1.0	2.61	0.28	1.81	0.20	0.97	0.096	0.03	0.10	0.013	0.030
6	1.0	1.0	3.20	0.36	2.22	0.25	0.81	0.076	0.16	0.08	0.013	0.025
7	1.0	1.0	4.27	0.59	2.96	0.41	0.69	0.067	0.37	0.07	0.019	0.019
8	1.0	1.0	3.55	0.46	2.46	0.32	0.75	0.077	0.23	0.08	0.017	0.022
9	1.0	1.0	3.11	0.38	2.15	0.26	0.78	0.080	0.16	0.08	0.014	0.026
			**Avg $\tau^{1/2}$ (s)**	**2.36**	**Std $\tau^{1/2}$ (s)**	**0.374**			**Avg D**	**0.024**	**Std D**	**0.0038**

Supplementary Table 13 (continued)

[Charge] = 25 mM, [NaCl] = 12.5 mM

Trial	ROI (μm)	Bckgd (μm)	τ (s)	std τ (s)	$\tau^{1/2}$ (s)	std $\tau^{1/2}$ (s)	A	Std A	C	std C	χ^2	D (μm^2 s^{-1})
1	1.0	1.0	4.13	0.41	2.86	0.28	0.79	0.055	0.23	0.06	0.012	0.019
2	1.0	1.0	4.42	0.58	3.07	0.41	0.64	0.058	0.35	0.06	0.015	0.018
3	1.0	1.0	3.54	0.40	2.45	0.28	0.73	0.064	0.23	0.07	0.012	0.022
4	1.0	1.0	3.46	0.43	2.39	0.30	0.69	0.068	0.26	0.07	0.013	0.023
5	1.0	1.0	3.26	0.37	2.26	0.26	0.78	0.073	0.21	0.08	0.013	0.024
6	1.0	1.0	4.12	0.50	2.86	0.35	0.68	0.059	0.35	0.06	0.014	0.019
7	1.0	1.0	3.32	0.42	2.30	0.29	0.60	0.062	0.34	0.06	0.010	0.024
8	1.0	1.0	3.74	0.48	2.59	0.33	0.64	0.062	0.35	0.06	0.013	0.021
9	1.0	1.0	3.96	0.51	2.75	0.35	0.70	0.066	0.35	0.07	0.016	0.020
			**Avg $\tau^{1/2}$ (s)**	**2.61**	**Std $\tau^{1/2}$ (s)**	**0.283**			**Avg D**	**0.021**	**Std D**	**0.0023**

[Charge] = 25 mM, [NaCl] = 50 mM

Trial	ROI (μm)	Bckgd (μm)	τ (s)	std τ (s)	$\tau^{1/2}$ (s)	std $\tau^{1/2}$ (s)	A	Std A	C	std C	χ^2	D (μm^2 s^{-1})
1	1.0	1.0	2.67	0.31	1.85	0.21	0.74	0.077	0.18	0.08	0.009	0.030
2	1.0	1.0	1.94	0.29	1.34	0.20	0.90	0.144	0.08	0.15	0.014	0.041
3	1.0	1.0	2.40	0.15	1.67	0.10	0.93	0.008	0.08	0.00	0.014	0.033
4	1.0	1.0	2.32	0.14	1.61	0.09	0.88	0.007	0.05	0.00	0.012	0.034
5	1.0	1.0	2.20	0.13	1.53	0.09	0.85	0.007	0.10	0.00	0.011	0.036
6	1.0	1.0	2.09	0.28	1.45	0.19	0.92	0.123	0.08	0.13	0.012	0.038
7	1.0	1.0	2.12	0.31	1.47	0.21	0.87	0.128	0.16	0.13	0.014	0.038
8	1.0	1.0	1.55	0.25	1.07	0.17	0.94	0.182	0.00	0.18	0.011	0.051
9	1.0	1.0	1.91	0.27	1.32	0.19	0.94	0.144	0.07	0.15	0.013	0.042
			**Avg $\tau^{1/2}$ (s)**	**1.48**	**Std $\tau^{1/2}$ (s)**	**0.225**			**Avg D**	**0.038**	**Std D**	**0.0062**

[Charge] = 50 mM, [NaCl] = 25 mM

Trial	ROI (μm)	Bckgd (μm)	τ (s)	std τ (s)	$\tau^{1/2}$ (s)	std $\tau^{1/2}$ (s)	A	Std A	C	std C	χ^2	D (μm^2 s^{-1})
1	1.0	1.0	2.15	0.30	1.49	0.21	0.77	0.107	0.19	0.11	0.010	0.037
2	1.0	1.0	2.49	0.36	1.73	0.25	0.80	0.109	0.29	0.11	0.015	0.032
3	1.0	1.0	1.81	0.26	1.25	0.18	1.00	0.155	0.01	0.16	0.013	0.044
4	1.0	1.0	2.45	0.35	1.70	0.24	0.79	0.107	0.26	0.11	0.014	0.032
5	1.0	1.0	2.84	0.34	1.97	0.24	0.76	0.081	0.23	0.08	0.012	0.028
6	1.0	1.0	1.72	0.13	1.19	0.09	0.89	0.008	0.10	0.00	0.014	0.046
7	1.0	1.0	1.65	0.25	1.15	0.18	1.00	0.175	0.05	0.18	0.013	0.048
8	1.0	1.0	1.86	0.26	1.29	0.18	0.99	0.150	0.00	0.15	0.013	0.043
9	1.0	1.0	1.48	0.23	1.02	0.16	0.97	0.182	0.00	0.18	0.010	0.054
			**Avg $\tau^{1/2}$ (s)**	**1.42**	**Std $\tau^{1/2}$ (s)**	**0.318**			**Avg D**	**0.040**	**Std D**	**0.0086**

[Charge] = 50 mM, [NaCl] = 100 mM

Trial	ROI (μm)	Bckgd (μm)	τ (s)	std τ (s)	$\tau^{1/2}$ (s)	std $\tau^{1/2}$ (s)	A	Std A	C	std C	χ^2	D (μm^2 s^{-1})
1	1.0	1.0	1.20	0.27	0.83	0.19	1.00	0.306	0.00	0.31	0.013	0.066
2	1.0	1.0	1.41	0.30	0.98	0.21	1.00	0.268	0.09	0.27	0.018	0.056
3	1.0	1.0	1.51	0.29	1.04	0.20	1.00	0.233	0.10	0.23	0.017	0.053
4	1.0	1.0	1.26	0.25	0.87	0.17	0.94	0.251	0.00	0.25	0.011	0.063
5	1.0	1.0	1.32	0.26	0.92	0.18	0.94	0.244	0.00	0.25	0.012	0.060
6	1.0	1.0	1.34	0.29	0.93	0.20	1.00	0.278	0.06	0.28	0.016	0.059
7	1.0	1.0	1.37	0.27	0.95	0.19	1.00	0.253	0.04	0.25	0.014	0.058
8	1.0	1.0	1.18	0.27	0.82	0.19	0.96	0.310	0.00	0.31	0.013	0.067
9	1.0	1.0	1.12	0.26	0.77	0.18	1.00	0.332	0.02	0.33	0.117	0.071
			**Avg $\tau^{1/2}$ (s)**	**0.90**	**Std $\tau^{1/2}$ (s)**	**0.085**			**Avg D**	**0.062**	**Std D**	**0.0058**

Supplementary Table 14. Starting 7.5 and 10 mM polymer (charge) mimics – whole droplet FRAP fitting parameters.

[Charge] = 7.5 mM, [NaCl] = 5 mM

Trial	ROI (μm)	Bckgd (μm)	τ (s)	std τ (s)	$\tau^{1/2}$ (s)	std $\tau^{1/2}$ (s)	A	Std A	C	std C	χ^2	D (μm^2 s^{-1})
1	6.00	6.50	885.1	22.0	613.5	15.2	0.94	0.009	0.28	0.004	0.00043	0.003
2	7.40	7.00	784.5	22.2	543.8	15.4	0.70	0.007	0.30	0.004	0.00034	0.006
3	7.70	6.00	827.8	25.0	573.8	17.3	0.76	0.009	0.25	0.004	0.00040	0.006
4	8.00	6.80	996.4	43.4	690.7	30.1	0.78	0.015	0.28	0.005	0.00056	0.005
5	8.00	7.20	888.5	29.1	615.8	20.1	0.66	0.009	0.27	0.004	0.00031	0.006
6	6.80	7.20	702.1	17.1	486.7	11.9	0.79	0.006	0.30	0.004	0.00041	0.005
			**Avg $\tau^{1/2}$ (s)**	**587.4**	**Std $\tau^{1/2}$ (s)**	**69.8**			**Avg D**	**0.005**	**Std D**	**0.0009**

[Charge] = 10 mM, [NaCl] = 5 mM

Trial	ROI (μm)	Bckgd (μm)	τ (s)	std τ (s)	$\tau^{1/2}$ (s)	std $\tau^{1/2}$ (s)	A	Std A	C	std C	χ^2	D (μm^2 s^{-1})
1	6.90	6.00	555.0	0.9	384.7	0.6	0.79	0.004	0.15	0.004	0.00042	0.007
2	7.20	6.10	545.3	8.9	378.0	6.2	0.79	0.004	0.22	0.004	0.00037	0.008
3	6.40	7.40	530.3	8.8	367.6	6.1	0.76	0.004	0.18	0.004	0.00036	0.006
4	6.70	6.80	568.1	10.1	393.8	7.0	0.79	0.004	0.19	0.004	0.00041	0.006
5	7.20	6.50	642.3	12.6	445.2	8.7	0.72	0.004	0.25	0.004	0.00033	0.006
6	6.80	6.00	445.5	11.5	308.8	8.0	0.83	0.007	0.28	0.008	0.00133	0.008
			**Avg $\tau^{1/2}$ (s)**	**379.7**	**Std $\tau^{1/2}$ (s)**	**44.0**			**Avg D**	**0.007**	**Std D**	**0.0008**

[Charge] = 37.5 mM, [NaCl] = 25 mM

Trial	ROI (μm)	Bckgd (μm)	τ (s)	std τ (s)	$\tau^{1/2}$ (s)	std $\tau^{1/2}$ (s)	A	Std A	C	std C	χ^2	D (μm^2 s^{-1})
1	8.20	7.50	81.9	0.8	56.8	0.6	0.86	0.005	0.13	0.006	0.00033	0.065
2	8.00	7.40	93.1	0.9	64.5	0.6	0.91	0.005	0.10	0.005	0.00035	0.055
3	7.40	7.40	73.5	1.0	50.9	0.7	0.80	0.007	0.18	0.007	0.00052	0.059
4	8.10	7.30	64.7	0.8	44.8	0.5	0.76	0.006	0.12	0.006	0.00030	0.080
5	8.00	7.30	108.2	1.4	75.0	1.0	0.81	0.006	0.30	0.006	0.00054	0.047
6	7.70	7.20	58.3	0.8	40.4	0.6	0.66	0.007	0.25	0.007	0.00032	0.081
			**Avg $\tau^{1/2}$ (s)**	**55.4**	**Std $\tau^{1/2}$ (s)**	**12.8**			**Avg D**	**0.064**	**Std D**	**0.0138**

[Charge] = 50 mM, [NaCl] = 25 mM

Trial	ROI (μm)	Bckgd (μm)	τ (s)	std τ (s)	$\tau^{1/2}$ (s)	std $\tau^{1/2}$ (s)	A	Std A	C	std C	χ^2	D (μm^2 s^{-1})
1	7.80	10.00	62.1	0.8	43.0	0.6	0.79	0.007	0.18	0.007	0.00041	0.078
2	7.60	8.10	69.6	1.0	48.3	0.7	0.90	0.008	0.21	0.009	0.00066	0.066
3	8.30	6.50	71.4	1.0	49.5	0.7	0.77	0.007	0.23	0.007	0.00047	0.077
4	7.70	7.00	52.8	0.6	36.6	0.4	0.77	0.006	0.18	0.006	0.00025	0.089
5	7.90	7.10	53.0	0.7	36.7	0.5	0.77	0.007	0.24	0.008	0.00038	0.093
6	7.90	7.00	42.8	0.7	29.7	0.5	0.71	0.009	0.20	0.009	0.00038	0.116
			**Avg $\tau^{1/2}$ (s)**	**40.6**	**Std $\tau^{1/2}$ (s)**	**7.7**			**Avg D**	**0.086**	**Std D**	**0.0174**

Supplementary Table 14 (continued)

[Charge] = 75 mM, [NaCl] = 50 mM

Trial	ROI (μm)	Bckgd (μm)	τ (s)	std τ (s)	$\tau^{1/2}$ (s)	std $\tau^{1/2}$ (s)	A	Std A	C	std C	χ^2	D (μm^2 s^{-1})
1	8.80	7.40	8.8	0.1	6.1	0.1	0.95	0.012	0.00	0.012	0.00031	0.695
2	9.20	8.20	12.5	0.1	8.6	0.1	0.90	0.007	0.06	0.007	0.00022	0.538
3	8.40	8.10	10.9	0.1	7.6	0.1	0.93	0.010	0.06	0.010	0.00032	0.512
4	9.20	8.00	14.1	0.1	9.8	0.1	1.00	0.006	0.02	0.007	0.00026	0.475
5	9.10	8.00	9.1	0.1	6.3	0.1	0.94	0.011	0.00	0.011	0.00027	0.725
6	8.30	7.10	11.5	0.1	7.9	0.1	1.00	0.011	0.03	0.011	0.00044	0.477
			**Avg $\tau^{1/2}$ (s)**	**7.7**	**Std $\tau^{1/2}$ (s)**	**1.4**			**Avg *D***	**0.570**	**Std *D***	**0.1111**

[Charge] = 100 mM, [NaCl] = 50 mM

	ROI (μm)	Bckgd (μm)	τ (s)	std τ (s)	$\tau^{1/2}$ (s)	std $\tau^{1/2}$ (s)	A	Std A	C	std C	χ^2	D (μm^2 s^{-1})
1	7.70	7.40	8.3	0.1	5.8	0.1	1.00	0.018	0.01	0.018	0.00037	0.567
2	7.50	9.10	6.8	0.2	4.7	0.1	1.00	0.031	0.00	0.031	0.00046	0.656
3	7.20	9.10	6.7	0.1	4.7	0.1	1.00	0.022	0.00	0.022	0.00048	0.613
4	7.50	7.00	6.8	0.2	4.7	0.1	0.98	0.024	0.00	0.024	0.00058	0.660
5	8.10	7.00	6.8	0.1	4.7	0.1	0.98	0.021	0.00	0.021	0.00044	0.770
6	8.10	6.80	6.7	0.1	4.7	0.1	1.00	0.021	0.04	0.021	0.00043	0.773
7	8.30	6.10	5.9	0.1	4.1	0.1	1.00	0.028	0.00	0.028	0.00053	0.928
8	8.30	6.40	6.2	0.1	4.3	0.1	1.00	0.027	0.00	0.027	0.00056	0.879
9	8.20	6.80	7.0	0.1	4.9	0.1	0.95	0.019	0.00	0.019	0.00042	0.760
10	8.70	7.10	7.1	0.1	4.9	0.1	1.00	0.017	0.05	0.018	0.00036	0.842
			**Avg $\tau^{1/2}$ (s)**	**4.7**	**Std $\tau^{1/2}$ (s)**	**0.4**			**Avg *D***	**0.745**	**Std *D***	**0.1187**

Supplementary Table 15. 7.5 and 10 mM [charge] mimics – partial droplet FRAP fitting parameters.

[Charge] = 7.5 mM, [NaCl] = 5 mM

Trial	ROI (μm)	Bckgd (μm)	τ (s)	std τ (s)	$\tau^{1/2}$ (s)	std $\tau^{1/2}$ (s)	A	Std A	C	std C	χ^2	D (μm^2 s^{-1})
1	1.0	1.0	4.06	0.47	2.81	0.33	0.85	0.072	0.20	0.08	0.019	0.020
2	1.0	1.0	5.71	0.68	3.96	0.47	0.74	0.050	0.29	0.06	0.018	0.014
3	1.0	1.0	4.65	0.60	3.23	0.42	0.67	0.058	0.28	0.06	0.016	0.017
4	1.0	1.0	4.94	0.58	3.42	0.40	0.76	0.057	0.28	0.06	0.018	0.016
5	1.0	1.0	3.60	0.41	2.49	0.29	0.81	0.072	0.12	0.07	0.015	0.022
6	1.0	1.0	3.13	0.22	2.17	0.15	0.74	0.009	0.20	0.00	0.015	0.025
7	1.0	1.0	3.36	0.51	2.33	0.35	0.75	0.090	0.21	0.09	0.021	0.024
8	1.0	1.0	3.26	0.46	2.26	0.32	0.81	0.095	0.19	0.10	0.022	0.024
9	1.0	1.0	3.70	0.50	2.56	0.35	0.75	0.077	0.19	0.08	0.019	0.021
10	1.0	1.0	5.65	0.74	3.92	0.51	0.77	0.058	0.36	0.06	0.023	0.014
			**Avg $\tau^{1/2}$ (s)**	**2.91**	**Std $\tau^{1/2}$ (s)**	**0.674**			**Avg D**	**0.020**	**Std D**	**0.0043**

[Charge] = 10 mM, [NaCl] = 5 mM

Trial	ROI (μm)	Bckgd (μm)	τ (s)	std τ (s)	$\tau^{1/2}$ (s)	std $\tau^{1/2}$ (s)	A	Std A	C	std C	χ^2	D (μm^2 s^{-1})
1	1.0	1.0	3.92	0.44	2.72	0.30	0.76	0.063	0.18	0.07	0.014	0.020
2	1.0	1.0	3.83	0.43	2.66	0.30	0.83	0.066	0.15	0.07	0.014	0.021
3	1.0	1.0	3.10	0.47	2.15	0.33	0.69	0.078	0.13	0.08	0.013	0.026
4	1.0	1.0	4.65	0.55	3.23	0.38	0.76	0.060	0.20	0.06	0.018	0.017
5	1.0	1.0	5.86	0.67	4.06	0.47	0.75	0.048	0.26	0.05	0.017	0.014
6	1.0	1.0	4.50	0.57	3.12	0.40	0.69	0.060	0.28	0.06	0.016	0.018
7	1.0	1.0	5.08	0.57	3.52	0.39	0.75	0.053	0.23	0.06	0.016	0.016
8	1.0	1.0	4.62	0.55	3.20	0.38	0.70	0.056	0.25	0.06	0.015	0.017
9	1.0	1.0	4.88	0.56	3.38	0.39	0.69	0.052	0.33	0.06	0.014	0.016
10	1.0	1.0	3.56	0.41	2.47	0.28	0.80	0.072	0.12	0.07	0.015	0.022
			**Avg $\tau^{1/2}$ (s)**	**3.05**	**Std $\tau^{1/2}$ (s)**	**0.560**			**Avg D**	**0.019**	**Std D**	**0.0036**

[Charge] = 37.5 mM, [NaCl] = 25 mM

Trial	ROI (μm)	Bckgd (μm)	τ (s)	std τ (s)	$\tau^{1/2}$ (s)	std $\tau^{1/2}$ (s)	A	Std A	C	std C	χ^2	D (μm^2 s^{-1})
1	1.0	1.0	2.72	0.29	1.89	0.20	1.00	0.017	0.10	0.00	0.056	0.029
2	1.0	1.0	2.83	0.38	1.96	0.26	0.98	0.115	0.04	0.12	0.023	0.028
3	1.0	1.0	2.98	0.41	2.07	0.29	0.86	0.101	0.16	0.10	0.021	0.027
4	1.0	1.0	2.52	0.32	1.75	0.22	0.93	0.110	0.01	0.11	0.016	0.032
5	1.0	1.0	2.96	0.38	2.05	0.26	0.94	0.103	0.05	0.11	0.021	0.027
6	1.0	1.0	2.69	0.40	1.86	0.28	0.89	0.118	0.12	0.12	0.022	0.030
7	1.0	1.0	2.41	0.17	1.67	0.12	0.90	0.009	0.10	0.00	0.018	0.033
8	1.0	1.0	2.82	0.39	1.96	0.27	0.82	0.099	0.19	0.10	0.017	0.028
9	1.0	1.0	2.46	0.16	1.71	0.11	1.00	0.010	0.00	0.00	0.021	0.032
10	1.0	1.0	2.22	0.34	1.54	0.24	0.91	0.138	0.04	0.14	0.019	0.036
			**Avg $\tau^{1/2}$ (s)**	**1.84**	**Std $\tau^{1/2}$ (s)**	**0.174**			**Avg D**	**0.030**	**Std D**	**0.0030**

[Charge] = 50 mM, [NaCl] = 25 mM

Trial	ROI (μm)	Bckgd (μm)	τ (s)	std τ (s)	$\tau^{1/2}$ (s)	std $\tau^{1/2}$ (s)	A	Std A	C	std C	χ^2	D (μm^2 s^{-1})
1	1.0	1.0	2.37	0.40	1.64	0.27	0.90	0.144	0.21	0.15	0.024	0.033
2	1.0	1.0	3.16	0.53	2.19	0.36	0.76	0.105	0.26	0.11	0.025	0.025
3	1.0	1.0	3.95	0.60	2.73	0.41	0.64	0.071	0.31	0.07	0.018	0.020
4	1.0	1.0	3.35	0.53	2.32	0.37	0.74	0.095	0.28	0.10	0.023	0.024
5	1.0	1.0	2.65	0.43	1.84	0.30	0.79	0.117	0.19	0.12	0.021	0.030
6	1.0	1.0	2.43	0.39	1.68	0.27	0.84	0.128	0.14	0.13	0.020	0.033
7	1.0	1.0	2.18	0.37	1.51	0.26	0.84	0.144	0.14	0.15	0.019	0.036
8	1.0	1.0	2.78	0.38	1.93	0.26	0.81	0.098	0.16	0.10	0.016	0.029
9	1.0	1.0	2.81	0.49	1.95	0.34	0.64	0.099	0.34	0.10	0.017	0.028
10	1.0	1.0	3.36	0.48	2.33	0.33	0.73	0.084	0.26	0.09	0.018	0.024
11	1.0	1.0	3.00	0.41	2.08	0.29	0.78	0.092	0.15	0.09	0.017	0.026
			**Avg $\tau^{1/2}$ (s)**	**2.02**	**Std $\tau^{1/2}$ (s)**	**0.360**			**Avg D**	**0.028**	**Std D**	**0.0049**

Supplementary Table 15 (continued)

[Charge] = 75 mM, [NaCl] = 50 mM

Trial	ROI (μm)	Bckgd (μm)	τ (s)	std τ (s)	$\tau^{1/2}$ (s)	std $\tau^{1/2}$ (s)	A	Std A	C	std C	χ^2	D (μm^2 s^{-1})
1	1.0	1.0	1.06	0.31	0.74	0.22	0.99	0.436	0.00	0.44	0.017	0.075
2	1.0	1.0	1.88	0.37	1.31	0.26	1.00	0.210	0.14	0.21	0.027	0.042
3	1.0	1.0	1.55	0.32	1.08	0.22	1.00	0.242	0.03	0.24	0.020	0.051
4	1.0	1.0	1.38	0.31	0.96	0.21	1.00	0.284	0.04	0.29	0.019	0.057
5	1.0	1.0	1.27	0.35	0.88	0.24	1.00	0.367	0.08	0.37	0.024	0.062
6	1.0	1.0	1.57	0.32	1.09	0.22	0.92	0.220	0.08	0.22	0.017	0.051
7	1.0	1.0	1.47	0.31	1.02	0.21	0.98	0.252	0.00	0.25	0.018	0.054
8	1.0	1.0	1.30	0.31	0.90	0.22	0.99	0.314	0.00	0.32	0.018	0.061
9	1.0	1.0	1.23	0.30	0.85	0.21	0.96	0.321	0.00	0.32	0.016	0.064
10	1.0	1.0	1.31	0.30	0.91	0.21	1.00	0.300	0.02	0.30	0.018	0.060
			**Avg $\tau^{1/2}$ (s)**	**0.97**	**Std $\tau^{1/2}$ (s)**	**0.158**			**Avg *D***	**0.058**	**Std *D***	**0.0089**

[Charge] = 100 mM, [NaCl] = 50 mM

Trial	ROI (μm)	Bckgd (μm)	τ (s)	std τ (s)	$\tau^{1/2}$ (s)	std $\tau^{1/2}$ (s)	A	Std A	C	std C	χ^2	D (μm^2 s^{-1})
1	1.0	1.0	1.35	0.32	0.94	0.22	1.00	0.301	0.08	0.30	0.020	0.059
2	1.0	1.0	1.16	0.30	0.81	0.21	1.00	0.363	0.03	0.36	0.017	0.068
3	1.0	1.0	0.93	0.34	0.64	0.24	1.00	0.590	0.02	0.59	0.017	0.085
4	1.0	1.0	1.07	0.30	0.74	0.21	0.89	0.370	0.00	0.37	0.012	0.074
5	1.0	1.0	1.23	0.31	0.85	0.21	1.00	0.336	0.04	0.34	0.018	0.064
6	1.0	1.0	0.94	0.13	0.65	0.09	0.82	0.008	0.20	0.00	0.018	0.085
7	1.0	1.0	1.35	0.66	0.94	0.46	1.00	0.964	0.03	0.97	0.020	0.059
8	1.0	1.0	1.54	0.58	1.06	0.40	1.00	0.683	0.01	0.69	0.019	0.052
9	1.0	1.0	1.62	0.53	1.12	0.37	1.00	0.570	0.05	0.57	0.017	0.049
10	1.0	1.0	1.88	0.49	1.30	0.34	1.00	0.406	0.00	0.41	0.017	0.042
11	1.0	1.0	1.46	0.62	1.01	0.43	1.00	0.803	0.05	0.81	0.020	0.054
12	1.0	1.0	2.10	0.50	1.45	0.35	1.00	0.347	0.01	0.35	0.019	0.038
13	1.0	1.0	2.02	0.53	1.40	0.37	1.00	0.389	0.06	0.39	0.021	0.039
			**Avg $\tau^{1/2}$ (s)**	**0.99**	**Std $\tau^{1/2}$ (s)**	**0.268**			**Avg *D***	**0.059**	**Std *D***	**0.016**

Supplementary References

1. Martin, N.; Li, M.; Mann, S. Selective Uptake and Refolding of Globular Proteins in Coacervate Microdroplets. *Langmuir* **2016,** 32, 5881-5889.
2. Fothergill, J.; Li, M.; Davis, S. A.; Cunningham, J. A.; Mann, S. Nanoparticle-Based Membrane Assembly and Silicification in Coacervate Microdroplets as a Route to Complex Colloidosomes. *Langmuir* **2014,** 30, 14591-14596.
3. Tian, L. F.; Martin, N.; Bassindale, P. G.; Patil, A. J.; Li, M.; Barnes, A.; Drinkwater, B. W.; Mann, S. Spontaneous assembly of chemically encoded two-dimensional coacervate droplet arrays by acoustic wave patterning. *Nat Commun* **2016,** 7.
4. Tang, T. Y. D.; Hak, C. R. C.; Thompson, A. J.; Kuimova, M. K.; Williams, D. S.; Perriman, A. W.; Mann, S. Fatty acid membrane assembly on coacervate microdroplets as a step towards a hybrid protocell model. *Nat Chem* **2014,** 6, 527-533.
5. Williams, D. S.; Patil, A. J.; Mann, S. Spontaneous Structuration in Coacervate-Based Protocells by Polyoxometalate-Mediated Membrane Assembly. *Small* **2014,** 10, 1830-1840.
6. Booth, R.; Qiao, Y.; Li, M.; Mann, S. Spatial Positioning and Chemical Coupling in Coacervate-in-Proteinosome Protocells. *Angew Chem Int Edit* **2019,** 58, 9120-9124.
7. Poudyal, R. R.; Guth-Metzler, R. M.; Veenis, A. J.; Frankel, E. A.; Keating, C. D.; Bevilacqua, P. C. Template-directed RNA polymerization and enhanced ribozyme catalysis inside membraneless compartments formed by coacervates. *Nat Commun* **2019,** 10.
8. Hiemenz, P. C.; Lodge, T. P., *Polymer Chemistry, Second Edition*. Taylor & Francis, 2007.
9. Rodriguez-Garcia, M.; Surman, A. J.; Cooper, G. J. T.; Suarez-Marina, I.; Hosni, Z.; Lee, M. P.; Cronin, L. Formation of oligopeptides in high yield under simple programmable conditions. *Nat Commun* **2015,** 6.
10. Campbell, T. D.; Febrian, R.; McCarthy, J. T.; Kleinschmidt, H. E.; Forsythe, J. G.; Bracher, P. J. Prebiotic condensation through wet-dry cycling regulated by deliquescence. *Nat Commun* **2019,** 10.
11. Da Silva, L.; Maurel, M. C.; Deamer, D. Salt-Promoted Synthesis of RNA-like Molecules in Simulated Hydrothermal Conditions. *J Mol Evol* **2015,** 80, 86-97.
12. Mamajanov, I.; MacDonald, P. J.; Ying, J. Y.; Duncanson, D. M.; Dowdy, G. R.; Walker, C. A.; Engelhart, A. E.; Fernandez, F. M.; Grover, M. A.; Hud, N. V.; Schork, F. J. Ester Formation and Hydrolysis during Wet-Dry Cycles: Generation of Far-from-Equilibrium Polymers in a Model Prebiotic Reaction. *Macromolecules* **2014,** 47, 1334-1343.
13. Forsythe, J. G.; Yu, S. S.; Mamajanov, I.; Grover, M. A.; Krishnamurthy, R.; Fernandez, F. M.; Hud, N. V. Ester-Mediated Amide Bond Formation Driven by Wet-Dry Cycles: A Possible Path to Polypeptides on the Prebiotic Earth. *Angew Chem Int Edit* **2015,** 54, 9871-9875.
14. Kitadai, N.; Maruyama, S. Origins of building blocks of life: A review. *Geosci Front* **2018,** 9, 1117-1153.
15. Aponte, J. C.; McLain, H. L.; Dworkin, J. P.; Elsila, J. E. Aliphatic amines in Antarctic CR2, CM2, and CM1/2 carbonaceous chondrites. *Geochim Cosmochim Ac* **2016,** 189, 296-311.
16. Mulkidjanian, A. Y.; Bychkov, A. Y.; Dibrova, D. V.; Galperin, M. Y.; Koonin, E. V. Origin of first cells at terrestrial, anoxic geothermal fields. *P Natl Acad Sci USA* **2012,** 109, E821-E830.
17. Frankel, E. A.; Bevilacqua, P. C.; Keating, C. D. Polyamine/Nucleotide Coacervates Provide Strong Compartmentalization of Mg2+, Nucleotides, and RNA. *Langmuir* **2016,** 32, 2041-2049.

18. Wilson, C.; Szostak, J. W. Ribozyme catalysis. *Current Opinion in Structural Biology* **1992,** 2, 749-756.
19. Doherty, E. A.; Doudna, J. A. Ribozyme structures and mechanisms. *Annu Rev Biochem* **2000,** 69, 597-615.
20. Fu, J.; Fares, H. M.; Schlenoff, J. B. Ion-Pairing Strength in Polyelectrolyte Complexes. *Macromolecules* **2017,** 50, 1066-1074.
21. Fares, H. M.; Ghoussoub, Y. E.; Delgado, J. D.; Fu, J.; Urban, V. S.; Schlenoff, J. B. Scattering Neutrons along the Polyelectrolyte Complex/Coacervate Continuum. *Macromolecules* **2018,** 51, 4945-4955.
22. Cakmak, F. P.; Grigas, A. T.; Keating, C. D. Lipid Vesicle-Coated Complex Coacervates. *Langmuir* **2019,** 35, 7830-7840.
23. Li, L.; Srivastava, S.; Andreev, M.; Marciel, A. B.; de Pablo, J. J.; Tirrell, M. V. Phase Behavior and Salt Partitioning in Polyelectrolyte Complex Coacervates. *Macromolecules* **2018,** 51, 2988-2995.
24. Ilavsky, J.; Jemian, P. R. Irena: tool suite for modeling and analysis of small-angle scattering. *J Appl Crystallogr* **2009,** 42, 347-353.
